# Targeted Metabolomics With Ultraperformance Liquid Chromatography–Mass Spectrometry (UPLC-MS) Highlights Metabolic Differences in Healthy and Atopic Staffordshire Bull Terriers Fed Two Different Diets, A Pilot Study

**DOI:** 10.3389/fvets.2020.554296

**Published:** 2020-10-27

**Authors:** Robin Moore, Johanna Anturaniemi, Vidya Velagapudi, Jatin Nandania, Stella Maria Barrouin-Melo, Anna Hielm-Björkman

**Affiliations:** ^1^Department of Equine and Small Animal Medicine, Faculty of Veterinary Medicine, University of Helsinki, Helsinki, Finland; ^2^Metabolomics Unit, Institute for Molecular Medicine Finland (FIMM), HiLIFE, University of Helsinki, Helsinki, Finland; ^3^Department of Veterinary Anatomy, Pathology and Clinics, School of Veterinary Medicine and Zootechny, Federal University of Bahia, Salvador, Brazil

**Keywords:** raw meat-based diet, targeted metabolomics, canine atopic dermatitis, canine health, kibble diet, diet intervention

## Abstract

**Background:** While anecdotal evidence has long claimed that a raw meat–based diet (RMBD) improves the metabolic health of canines, no rigorous scientific study has clarified this issue. Canine atopic dermatitis (CAD) has also been linked to metabolic health, but its relation to diet remains poorly understood. This study investigates whether dietary choice is linked to metabolic health in healthy and CAD-diagnosed canines via targeted serum and urine metabolomic analysis of polar, non-ionic metabolites, as well as whether the underlying CAD condition modulates the response to nutritional intake.

**Materials and Methods:** Serum metabolites of client-owned Staffordshire bull terriers, divided into CAD-diagnosed (*n* = 14) and healthy (*n* = 6) cohorts, were studied. Urine metabolites of a subset of the CAD-diagnosed canines (*n* = 8) were also studied. The canines were split into two cohorts based on diet. The first cohort were fed a commercially available high-fat, moderate-protein, low-carbohydrate RMBD (*n* = 11, CAD diagnosed *n* = 8, healthy *n* = 3). Those in the second cohort were fed a commercially available moderate-fat, moderate-protein, high-carbohydrate kibble diet (KD) (*n* = 9: CAD diagnosed *n* = 6, healthy *n* = 3). The diet intervention period lasted approximately 4.5 months (median 135 days). Statistical analyses of the serum profiles across all dogs (*n* = 20) and the urine profiles of the CAD-diagnosed subset (*n* = 8) were performed.

**Results and Discussion:** The KD cohort was found to have higher concentrations of methionine than the RMBD cohort, both in serum (all dogs, *p* < 0.0001) and in urine (CAD-only cohort, *p* < 0.0002), as well as cystathionine and 4-pyridoxic acid. Methionine plays important roles in homocysteine metabolism, and elevated levels have been implicated in various pathologies. The CAD (*n* = 14) cohort dogs showed starker metabolic changes in response to diet regarding these pathways compared to the healthy (*n* = 6) cohort. However, there was no significant change in CAD severity as a result of either diet. Likely due to the higher meat content of the RMBD, higher concentrations of several carnitines and creatine were found in the RMBD cohort. Citrulline was found in higher concentrations in the KD cohort. Our findings provide insight into the relationship between diet and the serum and urine metabolite profiles of canines. They also suggest that neither diet significantly affected CAD severity.

## Introduction

With the recent advancements in the field of metabolomics, an emerging approach to studying mammalian health, it has become easier to study and understand the relationship between an individual's metabolome and environmental factors (1). As the key concept of metabolomics is that the metabolic state of an organism represents the “overall physiological status of the organism” (2), the nascent field of canine nutritional metabolomics holds potential for both improving our understanding of canine disease risk factors and the underlying causes behind those risks (3). Here, we incorporate metabolomics as a novel approach to understanding the links between canine disease and diet. Study of the effects of nutritional intake on a canine's blood serum biochemistry can be complemented with the simultaneous analysis of the metabolomic profile of the urine. An excess of a polar metabolite's concentration in blood above the needs of an organism's normal function can be seen as an increase in the metabolite concentration in the urine as it exceeds the renal threshold for that compound (4). Through the use of combined media (blood serum and urine) in this study, we examine the extent to which the homeostasis of quantified blood metabolites is maintained and its relationship with food intake (4, 5).

The majority of domesticated dogs in the developed world eat a kibble diet (KD). According to the recently embraced NOVA food classification (6–8), kibble is an “ultraprocessed” product. Kibble is a mixture of ultraprocessed grains, such as wheat corn, and rice, mixed with ultraprocessed animal by-product meal and enriched with chemical additives, including synthetic vitamins, minerals, trace elements, preservatives, coloring agents, and palatability enhancers (9, 10). The raw meat–based diet (RMBD), in contrast, consists of raw animal parts. Complete and balanced commercial RMBDs also contain small amounts of raw vegetal matter as a source of fiber. The popularity of RMBDs is particularly high in Finland (11), but has also increased throughout the industrialized world (12). The possible health benefits of feeding dogs with RMBDs remain understudied in comparison to its popularity (13). In a recent review regarding the subject of raw feeding and its health effects (14), the authors concluded that there was insufficient evidence to evaluate the risks and benefits of RMBDs with regard to canine health. The NOVA classification of RMBDs is currently under debate. Although the raw ingredients themselves are minimally processed (8) (chopped, mixed, and frozen), minerals and vitamins are often added. The processing of the individual ingredients used to produce kibble may significantly alter their nutritional value and the overall health of the dog, although the reasons for this remain poorly understood (15, 16). The KD macronutrient profile differs remarkably from the RMBD profile. In terms of percent dry matter, a KD usually consists of a “protein:fat:carbohydrate” (PFC) macronutrient ratio 16–38%:6–18%:40–60%, whereas the PFC ratio of RMBD is typically %45:50%:0–10% (17).

Canine atopic dermatitis (CAD), part of the atopic complex, is a common systemic disease in canines and is considered a form of chronic inflammation or pruritus of the skin that manifests as an allergic response to an environmental factor (18). Clinical protocols for CAD diagnoses include the CADESI-4 scale and Favrot's criteria (19, 20). The development of CAD has been suggested to be genetically predisposed in canines, as well as further modulated by epigenetic factors (18). Phenotypically, the disease manifests itself differently in each individual (21), although there is a relatively consistent trait of elevated concentrations of the antibody immunoglobulin E across both atopy types and species (22, 23). Atopic dermatitis (AD) has been associated with several of the classic markers of metabolic syndrome (MetS) found both in humans (24) and in canines (25). This relationship, likely mediated via inflammatory markers, is not fully understood (26). The relationship between skin inflammation and oxidative stress markers in humans as a result of MetS has been studied (27), and several pathophysiological disease mechanisms that combine AD and MetS have been proposed (21, 28). Nutrition has been shown to have a vital role in determining the development of MetS through modulating metabolic pathways that have been attributed to the development of AD (22). CAD typically comprises both food-induced AD and non-food-induced AD (23). Although physiologically indistinguishable (19), they can be differentiated with the diet-restriction provocation trial (20). The link between metabolic health and CAD remains poorly studied. Most attention has focused on metabolic processes in the skin, especially in relation to fatty acids and lipids (29–32). It has long been known that the immune system of animals can be modulated by metabolites derived from nutrition (33). In canines, for example, vitamin D (34) and fatty acid supplementations (35–37) have been shown to have a protective effect against allergic pruritic responses.

Improving the length of pet health span remains a long-term goal in research of the health–nutrition axis. To achieve this, most research focuses on practical solutions, for example, improving diet to treat chronic disease in canines (38). Most research on dog diet and nutrition deals with improving food palatability (39, 40), modifying stool quality and nutrient absorption (41), all while meeting the daily caloric requirements. Little consideration of disease prevention has been reported in the literature (42). It has been well-established that a healthy diet in humans contributes to an increased health span and that an unhealthy diet increases the risk of many pathologies (43–45). In canines, studies to see whether certain diets help treat chronic diseases have mainly involved observing whether certain types of diets and functional foods appear to have a protective or therapeutic effect against chronic ailments (46–48).

This study is the metabolomics portion of a “nutriomics” research project, which has already been performed using a larger subset of samples from pet Staffordshire bull terriers by the DogRisk group (49, 50). The aim of our pilot study was to use targeted metabolomics to understand how nutrition relates to CAD and how nutrition and the CAD condition are related to canine blood and urine metabolite concentrations and canine metabolic health in general.

## Materials and Methods

### Design and Animals

**Figure 1:**
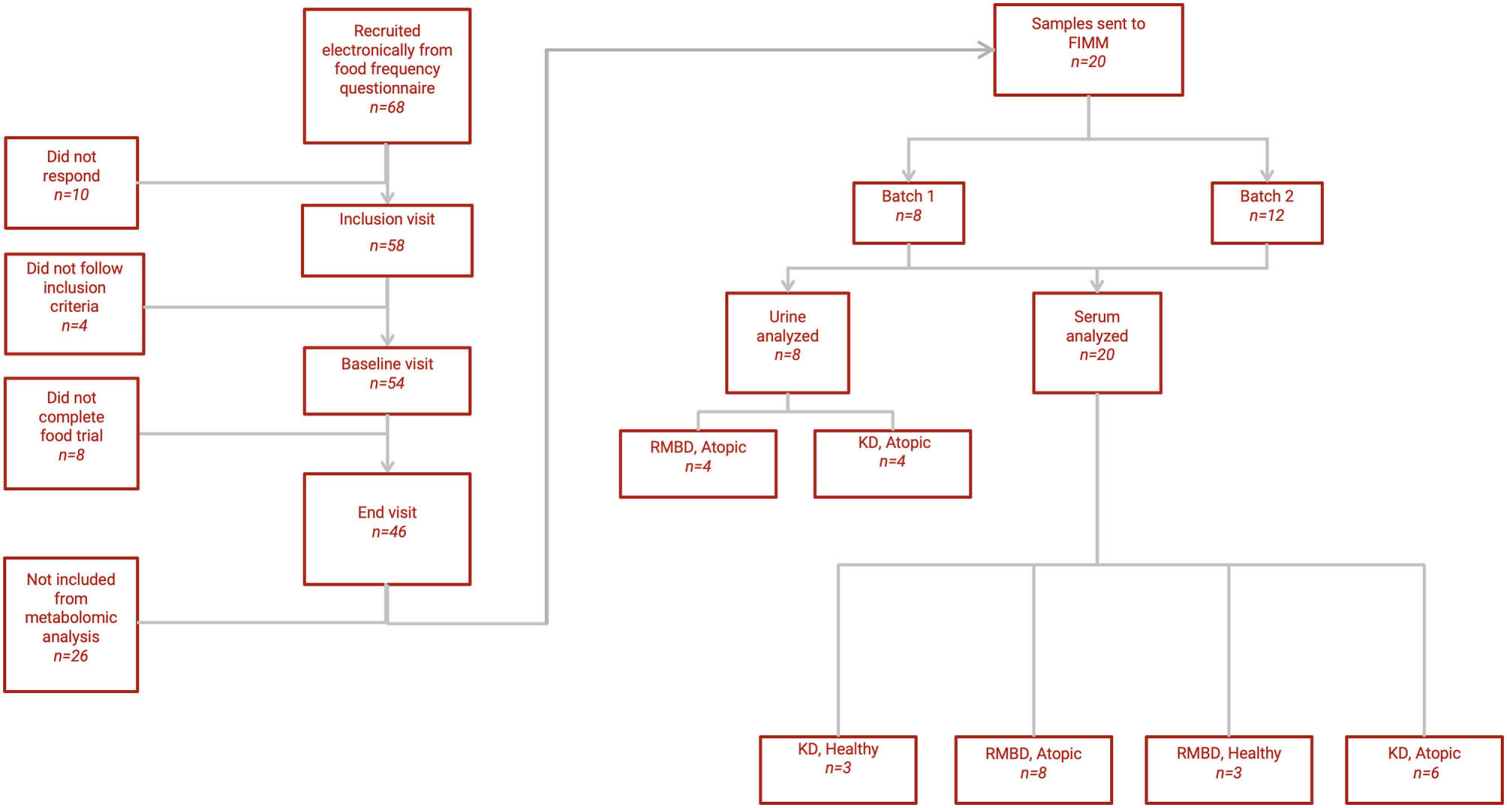
Flowchart of study. A flowchart depicting the selection process of the Staffordshire bull terriers used for the metabolomic analysis (*n* = 20) and how they resulted in the cohorts based on diet (KD, kibble diet; RMBD, raw meat–based diet) and health status (CAD, canine atopic dermatitis).

A flowchart of the diet intervention is shown in Figure 1. In this diet intervention study, initiated in 2013, client-owned pet Staffordshire bull terriers were first studied with nutrigenomic (49) and hematological (50) approaches. The family history of the dogs has been reported elsewhere (49). The diet intervention included inclusion, baseline, and end visits during the diet trial. No special inclusion diet was required prior to baseline, although the diet of each dog prior to their baseline visit was determined using a food frequency questionnaire. Of the original cohort of Staffordshire bull terriers that underwent the whole study and fulfilled all criteria of the diet trial (*n* = 46), only a subset (*n* = 20) was selected for serum metabolomic analysis due to high running costs. The subset (*n* = 20) was stratified based on owner-reported diets prior to baseline, as well as their diet during the study. All dogs analyzed for this study were fed solely kibble (KD) or raw food (RMBD) over a diet intervention period of 3 to 5 months (median = 135 days), i.e., forming a KD cohort (*n* = 9) and an RMBD cohort (*n* = 11). The dogs included in the analysis (*n* = 20) were also split into cohorts based on whether they were CAD-diagnosed (*n* = 14) or healthy (*n* = 6). For analysis that considered diet and health condition, the dogs were divided into four cohorts, healthy-KD (*n* = 3), CAD-KD (*n* = 6), healthy-RMBD (*n* = 3), CAD-RMBD (*n* = 8). Urine metabolomic analysis of samples collected at the end of the diet intervention was performed for a subset (*n* = 8) of only CAD-diagnosed individuals, also due to high costs of analysis. The baseline samples were collected in during September and October, and the end samples were all collected between February and April. The winter months were chosen for the diet intervention due to the seasonality of the disease, as CAD symptoms have been reported to be exacerbated as a result of pollen and blooming plant exposure (51, 52). Because of unrelated circumstances (pregnancy of the study coordinator), the trial ended later than planned. Seasonality possibly affected the disease phenotype, as the end visit was delayed in some cases to spring, when plants already started blooming in Finland.

The canines were evaluated before and after the diet intervention by a dermatologist, who used Favrot's criteria (19), the Canine Atopic Dermatitis Extent and Severity Index (CADESI-4) scale (20), and biochemical and hematological tests. The threshold for whether a canine suffered from CAD required a fulfillment of 5 of 8 of Favrot's criteria. The severity of the CAD was diagnosed using the CADESI-4 scale, which categorizes CAD severity as follows: 0–10 = in remission, 11–33 = mild CAD, 34–59= moderate CAD, ≥60 = severe CAD. All CAD-diagnosed canines in this study hence suffered from mild CAD. Owner-reported data regarding CAD severity as a visual analog scale to evaluate the level of pruritus at 2-week intervals from baseline to end was also collected. The owner-reported pruritus conflicted with the dermatologist's CAD severity evaluation in some cases. However, for clarity, only the diagnosis reported by the dermatologist was used in this study.

The diets used in the study were a commercial KD and two commercial RMBDs. The RMBDs used in this study had an average PFC macronutrient ratio of 26:74:0 percent metabolizable energy (% ME). The KD diet used in this study had a PFC macronutrient ratio of 23:36:41% ME (Table 1).

**Table 1:** Percent metabolizable energy (%ME) of the kibble (Hill's Science Plan) and two raw meat–based diets (MUSH BARF Vaisto, pork–chicken–lamb, beef–turkey–salmon).

**Macronutrient**	**Hill's Science Plan™ canine, adult sensitive skin with chicken (%ME)**	**MUSH Vaisto (pork–chicken–lamb) (%ME)**	**MUSH Vaisto (beef–turkey–salmon) (%ME)**	**MUSH diets combined average (%ME)**
Protein	23.28	23.84	28.09	25.96
Fat	35.76	76.16	71.91	74.04
Carbohydrate	40.95	0.00	0.00	0.00

%ME, % metabolizable energy.

*The values are calculated using the modified Atwater factors as suggested by the National Research Council (53)*.

The commercial dry diet used in this study was Hill's Science Plan™ Canine Adult Sensitive Skin With Chicken (detailed composition shown in Supplementary Table 1A). The two commercial raw meat diets used in this study were MUSH Vaisto® Pork–Chicken–Lamb and MUSH Vaisto® Beef–Turkey–Salmon (detailed compositions shown in Supplementary Table 1B). For the RMBDs, owners were free to either choose one or combine both diets. According to manufacturer claims, both the KD and the two RMBD options were “complete diets.” Owners were asked to feed their dogs 99.9% with the trial food using amounts recommended by the manufacturer, adjusting amounts if their dog's body weight would start to fall or rise. Owners reported the adherence to diet using a food diary. Water was allowed *ad libitum*.

### Samples

The metabolomic analysis of blood and urine samples were performed in two batches, i.e., batches 1 and 2. Both batches are described in Table 2. For batch 1, blood serum samples, collected at baseline and end, and urine samples collected only at end, from atopic dogs (*n* = 8) were used. For batch 2, only blood serum samples collected at baseline and end from a cohort of both atopic and healthy dogs were used (atopic *n* = 6, healthy *n* = 6). For analysis of serum, batches 1 and 2 were combined (atopic *n* = 14, healthy *n* = 6) for several of the analyses described below.

**Table 2:** Overview of the experimental setup of diet intervention, including division of Staffordshire bull terriers into diet cohorts (diet overview in Table 1), gender, health status, disease phenotype, diet intervention length, and age.

**Batch**	**1**		**2**		**1 and 2**	
**Diet cohort**	**RMBD**	**KD**	**RMBD**	**KD**	**RMBD**	**KD**
Dogs (total) (*n*)	4	4	7	5	11	9
Gender (male/female)	4/0	2/2	3/4	3/2	7/4	5/4
Sterilized (yes/no)	2/2	3/1	2/5	1/4	4/7	4/5
Blood serum analyzed	Yes	Yes	Yes	Yes	Yes	Yes
Urine analyzed	Yes	Yes	No	No	No	No
Atopy (total) (*n*)	4	4	4	2	8	6
NFIAD/FIAD	3/1	3/1	4/0	2/0	7/1	5/1
Healthy (*n*)	0	0	3	3	3	3
Mean diet intervention length (days) (SD)	126 (35.3)	141 (26.6)	137 (27.0)	136 (29.7)	133 (29.0)	139 (26.7)
Mean CADESI score at BL (SD)	CAD: 13.5 (9.0)	CAD: 19.0 (10.8)	CAD: 12.5 (8.7) Healthy: 3.3 (1.2)	CAD: 18.5 (16.3) Healthy: 2.7 (1.2)	CAD: 13 (8.3) Healthy: 3.3 (1.2)	CAD: 18.8 (11.1) Healthy: 2.7 (1.2)
Age at BL (months; mean, SD)	44.7 (34.9)	56.2 (31.7)	60.8 (35.9)	75.2 (46.1)	54.9 (34.7)	66.8 (39.3)

*RMBD, raw meat–based diet; KD, kibble diet; NFIAD, non–food-induced atopic dermatitis; FIAD, food-induced atopic dermatitis; SD, standard deviation; BL, baseline*.

Blood samples were collected from the jugular vein using Vacuette® 3 mL EDTA, 3 mL lithium heparin, and 6 mL plain serum tubes by a closed method (Vacutainer® Safety-Lok™ blood collection sets, Becton Dickinson, Meylan, France). Serum samples were allowed to clot at room temperature for 30 min before centrifugation (2,100 × g for 15 min). Urine samples were collected into factory-clean specimen jars and frozen after collection in 5 mL tubes. All samples were fasting samples collected in the morning. After collection they were stored at −80°C.

The targeted metabolomic analyses of the dogs' serum samples at baseline and end of the diet intervention (all dogs *n* = 20, healthy *n* = 6, atopic *n* = 14) were performed at the Finnish Institute of Molecular Medicine (FIMM) using targeted liquid chromatography (LC) mass spectrometry (MS). As targeted metabolomics of canine samples had not been performed before the first batch (batch 1, *n* = 8) was sent to FIMM to test the method. As the results were interpretable, more samples (batch 2, *n* = 12) were sent. Common polar, non-ionic metabolites (*n* = 102) were targeted with nanomolar accuracy (±0.005 μM) using the BioCrates p180 kit as standards for isotopic quantification, including amino acids, bile acids, nucleobases, nucleosides, choline metabolites, carbohydrates, and enzyme cofactors. A full list of the targeted metabolites used in the standard mixture is included in Supplementary File 20. A labeled internal standard mixture (10 μL) was added to 100 μL of serum or urine samples, which were all run in triplicate to ensure reliability. Metabolites were extracted by adding four parts (1:4, sample: extraction solvent) of the 100% acetonitrile + 1% formic acid solvent. The collected extracts were dispensed into Ostro™ 96-well plates (Waters Corporation, Milford, USA) and filtered by applying vacuum at a delta pressure of 300 to 400 mbar for 2.5 min using a robotic vacuum station. The filtrate was transferred to a 96-well collection plate, which was placed under the Ostro™ plate. The collection plate was sealed with the well cap mat and placed in the auto-sampler of the LC system for injection. Samples were analyzed using high-throughput targeted quantitative metabolic profiling using the ACQUITY ultraperformance LC–tandem MS (UPLC-MS/MS) instrument (Waters), with a 1.7-μm BEH amide HILIC column for chromatography.

### Data Preprocessing

Sample preparation for UPLC-MS/MS, as well as raw spectral data processing, was carried out on site by FIMM personnel. Subsequent concentration data were provided for each metabolite, along with comments regarding their reliability. The raw spectral data were acquired with MassLynx 4.1, and TargetLynx software. Detailed information regarding the raw spectrum metabolomics analysis, including chemicals and reagents, metabolite extraction protocol, and serum sample preparation, can be found elsewhere (54). All metabolomics instrumentation used for analysis was owned by and located in the FIMM metabolomics unit in Biomedicum (Metabolomics Unit, Finnish Institute for Molecular Medicine FIMM, Helsinki, Finland).

Based on LC-MS raw data processing, for batch 1, 80 of the original 102 targeted metabolites in serum samples (Supplementary Table 2A), and 80 of the original 102 metabolites in urine samples, were used in the statistical analysis (Supplementary Table 2B). The raw data from batch 2 were considerably better, and only one of the 102 metabolites, spermidine, had to be omitted from analysis. For the combined batch serum analysis, 79 of the original 102 metabolites were used for the statistical analysis (Supplementary Table 2C).

Original metabolite values in the serum and urine datasets were reported in μmol/L. Urine metabolite values were normalized to their respective creatinine concentrations. Urine metabolite values used in data analysis were adjusted to metabolite (μmol)/creatinine (mmol). Creatinine-adjusted urine metabolite values were used in the analysis that combines serum and urine datasets. Only usable metabolite concentration values found in both datasets were used. In summary, 72 of the original 102 metabolite values were used in the analysis that combines serum and urine metabolite values.

### Statistical Analysis

Statistical analysis was performed with the R package MetaboAnalystR (55). Source code for the statistical analysis workflow was documented as R-generated analysis reports (Supplementary Files 1–19). Targeted metabolites that were unreliably quantified or contained >50% missing values were removed with Excel prior to data processing with R. The integrity of all serum samples and urine samples were checked with R prior to data analysis. As metabolite concentrations fluctuate greatly, the raw concentration values in both serum and urine were log transformed using a generalized logarithm function, allowing the concentrations to assume a more normal distribution for subsequent analysis. To improve the sample size and hence statistical power for downstream analysis, batch correction for the end-of-diet time points of batches 1 and 2 serum data was performed using the ComBat empirical Bayes method developed by Johnson et al. (56) in order to combine the two cohorts as there was significant variation due to batch effect. Combined-batch analysis of serum concentrations from batches 1 and 2 used values generated with the K-nearest neighbor algorithm prior to their combination to estimate any remaining missing values. The similarity between batches 1 and 2 end values was analyzed with principal component analysis. A two-dimensional (2-D) principal component analysis plot of both pre-correction and post-correction is attached in Supplementary Figure 1. Each metabolite included in the combined-batch analysis was tested to see whether there was a significant difference between batches after batch correction using a *t*-test. No significant differences were observed due to batch after the batch correction was performed. In all of the metabolite datasets used in this study, the K-nearest neighbor algorithm was used to compute missing metabolite values for metabolites that were missing <50% of the values within each cohort.

For the results of statistical analysis, the cutoff for significance was set at false discovery rate (FDR) <0.05 (also referred to as the FDR-adjusted *p*-value or *q*-value in some tables). In all statistical analyses, *p*-values are reported. As a general rule for metabolomics analysis, the reporting of FDR values are recommended to ensure that results are statistically significant as the number of parameters tested is far greater than the number of samples (57). In essence, the FDR “controls the expected proportion of falsely rejected hypotheses” (58).

#### Univariate Analysis of Baseline and End of Diet Intervention

Univariate analysis of baseline serum values from batches 1 and 2, as well as the combined batch dataset with respect to diet cohorts and health status cohorts, was performed to confirm whether there were any significant metabolite concentration differences between either cohort at the baseline of the diet intervention. Analyses of diet and health were first performed separately. For both the baseline and end of diet intervention, a general linear model (GLM) and parametric *t*-tests were used to observe statistically significant fold changes between the RMBD and KD cohorts in batch 1 serum and urine samples, in batch 2 serum samples, and in the combined batch serum samples, i.e., analysis of all dogs in the study. Univariate analysis reports were created for each test between diet cohorts and health status both at the baseline and end of the diet intervention and can be found in Supplementary File 21.

#### Univariate Analysis of CADESI-4 Score, Weight, and Age With Diet

The change in CADESI-4 scores between diet cohorts was determined by testing the change (end timepoint minus baseline) to see whether diet correlated with change in phenotype. The same was done for weight and age. Changes in CADESI-4 scores were also compared within dietary cohorts between gender, as well as neutering status.

#### Analysis Between Sample Media and Dietary Cohorts at End of Diet Intervention

A two-way analysis of variance (ANOVA) was performed between sample media (blood or urine) and dietary cohorts (KD or RMBD). Hierarchical clustering was then combined with the results from the two-way ANOVA to generate heatmap visualizations of the significantly different metabolites between diet cohorts and sample type in the serum and urine data. The differences in variance between cohorts are also reported as *F*-values.

Fold-change comparisons combined with *t*-tests were used to identify significant differences between serum and urine metabolite concentrations. The GLM was then used to perform correlation analysis between samples and identify which significant metabolites correlate with diet. To visualize how the samples within cohorts contributed to significant metabolite differences observed with the GLM, heatmap visualizations of significant metabolites (FDR < 0.05) within individual batches, as well as combined batch results from Fisher least significant difference (LSD) test were created.

To further explore the results seen from *t*-tests and the ANOVA, a supervised multivariate regression-based analysis, partial least squares–discriminant analysis (PLS-DA), was used to test the significance between sample media and diet cohorts. This was performed to determine the extent to which the linear combination of the metabolite values for a given sample can predict the diet cohort of the dog. For each component, each metabolite was assigned a variable importance in projection (VIP) score. The VIP score signifies the relative contribution a given metabolite has to discriminating the cohorts that are compared in the model and is dependent on the percentage variation explained by the component vectors used in the model.

To observe the risk of overfitting when using PLS-DA, cross-validation using the leave-one-out approach (LOOCV) was used to determine the accuracy, R2 and Q2 values of each respective component, where Q2 values have been computed to resemble the scale used for R2 and accuracy scores (0< *x* <1). Loading plots for the components 1 and 2 (the two components that explain the most variation between cohorts) were visualized to show the relative contributions metabolites had to the creation of their respective component vector.

#### Analysis Between Diet and Atopy at End of Diet Intervention

Analysis of diet and health combined for batch 2 and combined batch datasets to test for interaction was also performed with a two-way ANOVA. As all dogs in batch 1 were diagnosed with atopy, no analysis with regard to health status was performed. For the combined batch dataset, the results from the end of the diet intervention were studied with a two-way ANOVA between diet and atopy and their interactions. Results were visualized with a heatmap. To further explore the results seen from *t*-tests and the ANOVA, PLS-DA was used to identify the extent to which the diet and atopy cohorts differed.

## Results

### Univariate Analysis of Baseline and End of Diet Intervention

By controlling for baseline bias, mildly significant concentration differences of arginine, histidine, and threonine between the two diet cohorts (*p* < 0.05, FDR > 0.05) were found (Supplementary Table 4). No significant metabolite concentration differences between atopic and healthy individuals were observed either at baseline or at the end of the diet intervention.

For all dogs' serum samples in the study (*n* = 20), the metabolites that significantly differ (FDR ≤ 0.05) between diet cohorts at the end of the diet intervention are presented in Table 3. A more comprehensive table of all dogs at the end of the diet intervention, significant differences between the diet cohorts of the batches separately, and only urine metabolites from the individuals of batch 1 (*n* = 8), as well as serum metabolites from only atopic dogs (*n* = 14), are included in Supplementary Tables 5–9).

**Table 3:** Comparison of significantly different metabolite concentrations in all dog's serum samples between kibble diet (KD, *n* = 9) and raw meat–based diet (RMBD, *n* = 11) at end of diet intervention.

**Metabolite**	**Mean (SD) of KD cohort**	**Mean (SD) of RMBD cohort**	***p***	***q* (FDR)**	**Fold change**	**In KD cohort**
Methionine	6.686 (0.294)	5.697 (0.305)	<0.0001	0	1.17	Up
4-Pyridoxic acid	−8.830 (0.460)	−11.025 (0.804)	<0.0001	0	−1.25	Up
Citrulline	5.659 (0.204)	4.654 (0.507)	<0.0001	0.0011	1.22	Up
Cytosine	−4.146 (0.790)	−5.964 (0.930)	0.0002	0.0026	−1.44	Up
Proline	7.965 (0.406)	7.099 (0.403)	0.0002	0.0026	1.12	Up
Cystathionine	3.154 (1.292)	0.152 (1.004)	0.0002	0.0026	20.78	Up
Taurochenodeoxycholic acid	−0.898 (0.762)	−3.255 (1.357)	0.0002	0.0026	−3.62	Up
Hexanoylcarnitine	−7.033 (0.484)	−5.937 (0.760)	0.0015	0.0148	1.18	Down
Decanoylcarnitine	−6.414 (0.485)	−5.443 (0.661)	0.0018	0.0156	1.18	Down
Glycine	8.629 (0.299)	8.049 (0.407)	0.0023	0.018	1.07	Up
Creatine	4.155 (0.616)	5.176 (0.753)	0.0043	0.0297	−1.25	Down
Kynurenine	0.849 (0.513)	0.242 (0.319)	0.0045	0.0297	3.51	Up
Dimethylglycine	2.369 (0.511)	1.606 (0.575)	0.0062	0.0374	1.48	Up
Trimethylamine-N-oxide	−3.100 (11.157)	1.534 (0.830)	0.0074	0.042	0.49	Down

SD, standard deviation; KD, kibble diet; RMBD, raw meat–based diet; FDR < 0.05, false discovery rate <0.05.

*Mean serum concentrations are presented as the natural log of the original metabolite concentration*.

At the end of the diet intervention, hexanoylcarnitine (FDR = 0.015, *p* = 0.0015), decanoylcarnitine (FDR = 0.016, *p* = 0.0018), octanoylcarnitine (FDR = 0.052, *p* = 0.01), acetylcarnitine (FDR = 0.086, *p* = 0.021), creatine (FDR = 0.03, *p* = 0.005), and creatinine (FDR = 0.15, *p* = 0.041) concentrations were higher in serum of the RMBD cohort than in the KD cohort (all dogs, *n* = 20). Higher serum concentrations of urea-cycle metabolites citrulline (FDR = 0.001, *p* < 0.0001) and proline (FDR = 0.002, *p* = 0.0002) and the nucleobase cytosine (FDR = 0.0026, *p* = 0.0002) were observed in all of the dogs of the KD cohort. Higher concentrations of the primary bile acid taurochenodeoxycholic acid (FDR = 0.0026, *p* = 0.0002) and taurocholic acid were found in the KD cohort relative to the RMBD cohort (1.87-fold higher concentration, FDR = 0.112, *p* = 0.028). Serum methionine concentrations were higher in the KD-fed dogs (FDR <0.0001, *p* < 0.0001), as well as cystathionine (FDR = 0.0026, *p* = 0.0002), dimethylglycine (FDR = 0.037, *p* = 0.0062), and 4-pyridoxic acid (FDR <0.0001, *p* < 0.0001). There were higher urine concentrations of betaine, the precursor to dimethylglycine, in the RMBD-fed cohort (FDR = 0.0022, *p* = 0.0008), as well as a trend in serum of all dogs (FDR = 0.086, *p* = 0.02). Notably, dogs from batch 1 in the KD cohort also had significantly higher urine concentrations of methionine (FDR <0.02, *p* < 0.0002) and 4-pyridoxic acid (FDR <0.04, *p* < 0.002) (Supplementary Table 6). There were no metabolites that significantly differed between diet cohorts of the healthy individuals (KD *n* = 3, RMBD *n* = 3), although several metabolite concentrations differed with *p* < 0.05 (FDR >0.05, *p* < 0.05) (Supplementary Table 10).

### Two-Way ANOVA Between Sample Media and Diet at End of the Diet Intervention

**Figure 2:**
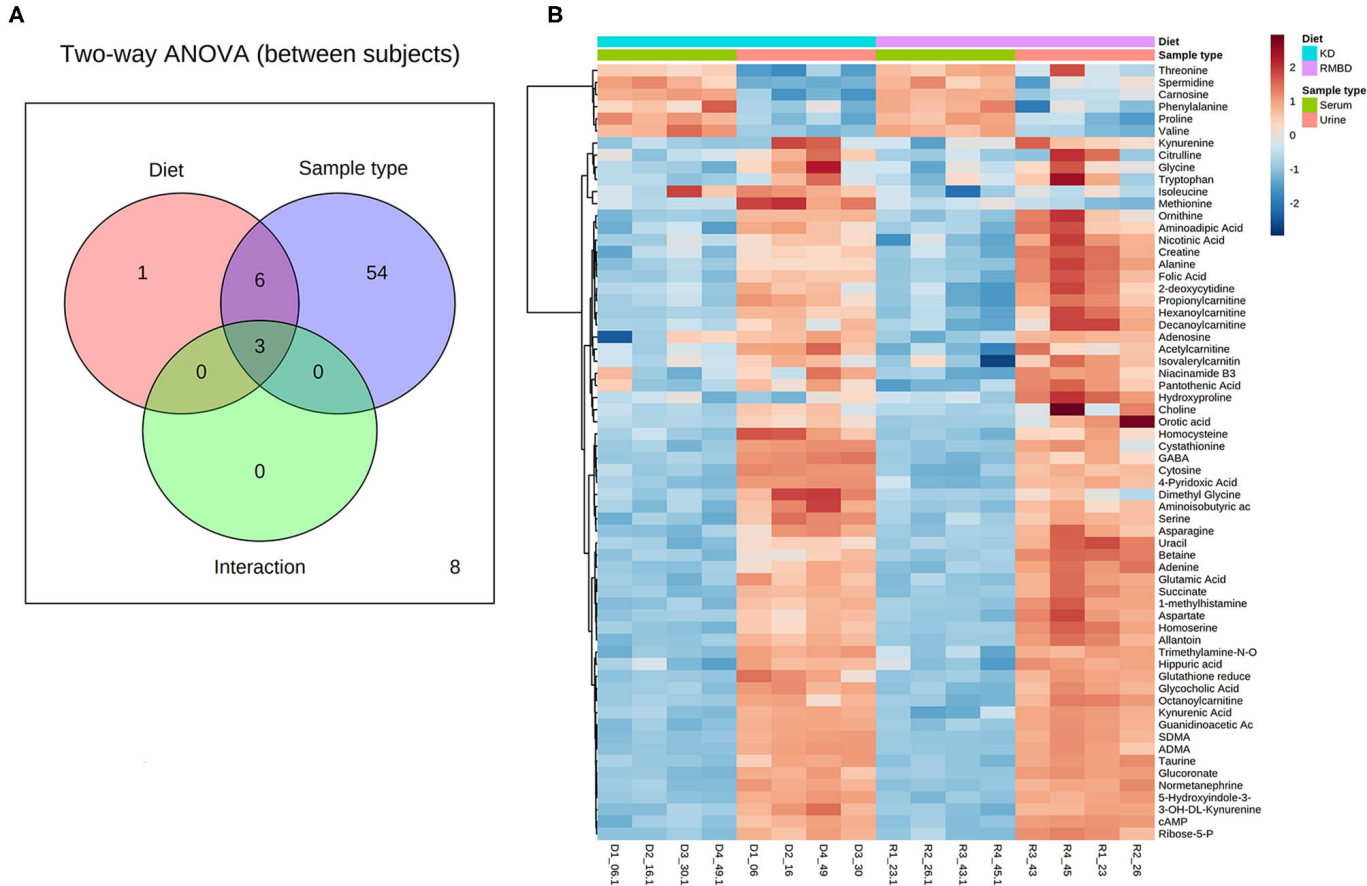
Batch 1 (*n* = 8) comparison of serum and urine profiles between diet cohorts. **(A)** An overview of sample media and diet interaction at the end of diet intervention where metabolite values differ significantly (FDR < 0.05) between diet cohorts (red) and sample type (blue), as well as interaction between the two (dark green and purple). **(B)** A heatmap illustrating significant features from the two-way ANOVA. Values relative to the combined cohort average are represented as a color spectrum and have been scaled to −2 (blue) through 2 (red) (KD, kibble diet; RMBD, raw meat–based diet).

**Figure 3:**
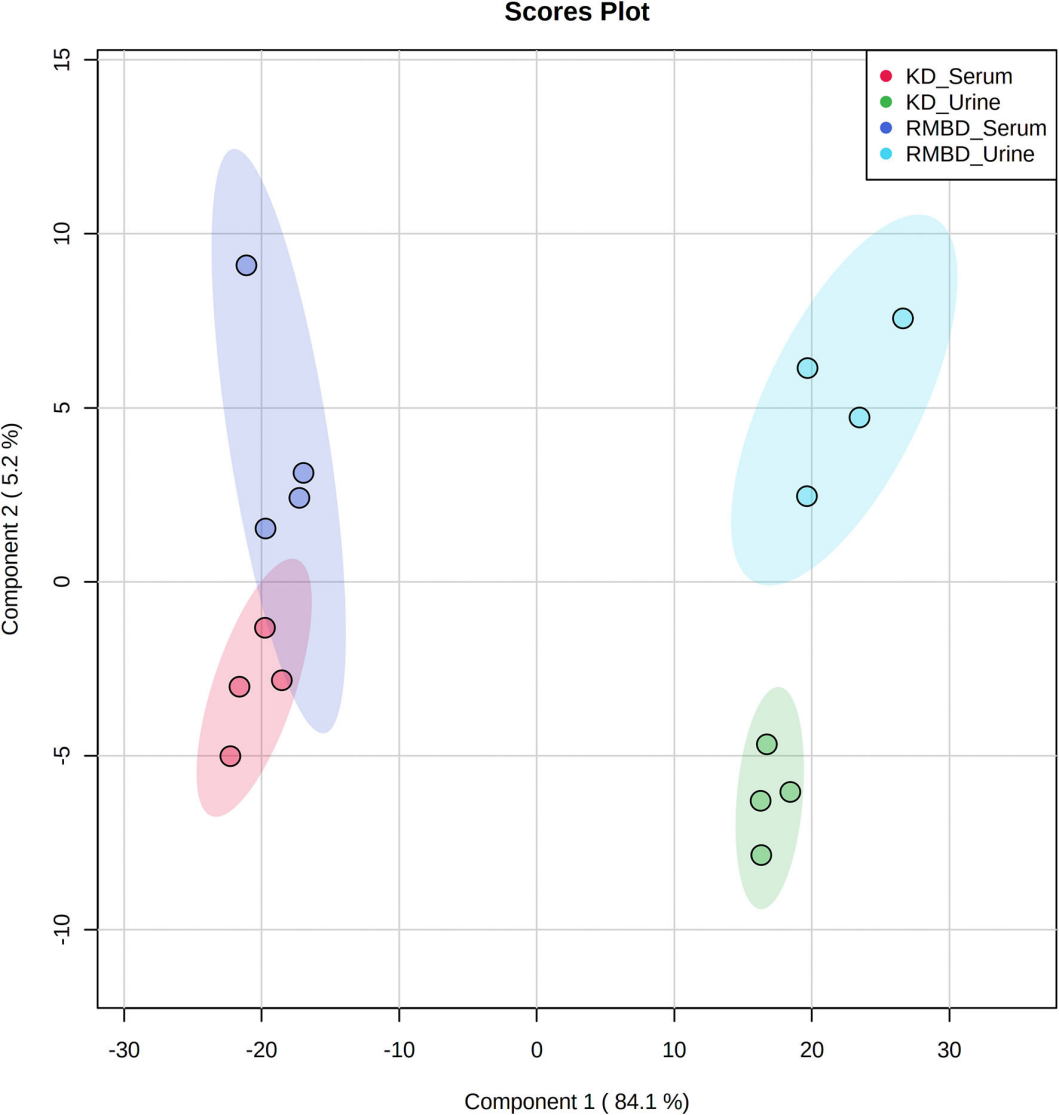
PLS-DA shows how the serum and urine profiles of batch 1 (*n* = 8) can separate diet cohorts. PLS-DA of batch 1 dogs (*n* = 8) at the end of the diet intervention. Plot shows how serum, urine, and the KD (*n* = 4) and RMBD (*n* = 4) cohort metabolites differed at the end of diet intervention, shown with 95% confidence intervals (shaded regions) (KD, kibble diet; RMBD, raw meat–based diet).

A two-way ANOVA was used to see whether any significant difference in serum metabolite concentrations between the diet cohorts could be seen in urine metabolite concentrations (Figure 2A). Of the 63 metabolites that differed significantly between serum and urine, 10 also differed between diet cohorts with interaction detected in five of the metabolites (Supplementary Table 10). The significantly different metabolites between diet cohorts and sample type (serum and urine) from the two-way ANOVA were visualized with a heatmap (Figure 2B). To further explore how urine and serum samples differed between the diet cohorts of batch 1, a PLS-DA was performed. The parameters of the model, calculated with the LOOCV approach, are shown in Supplementary Table 14A. Components 1 and 2 were plotted against each other (Figure 3) with shaded circles representing the 95% confidence interval area for the respective diet cohorts. In the 2-D PLS-DA plot presented in Figure 3, the extent to how much within-cohort variation exists for diet cohorts and urine and serum samples was visualized. When the first two components of the PLS-DA were plotted against each other, the urine and serum samples were separable with the first component, and the RMBD and KD diet cohorts were separable with the second component. However, likely because of the low sample size, the predictability of the model calculated with R2 and its predictability when testing the model (Q2) were 0.108, and as such can be considered quite weak. However, although the Q2 is small, the model describes the extent to which the sample media accounts for most of the variance. There was a minor overlap of confidence intervals between diet cohorts observed in serum samples when separated with component 2.

### Univariate Analysis of CADESI-4 Score, Weight, and Age With Diet

According to the evaluation of CAD severity at the end of the diet intervention, neither the KD nor the RMBD significantly changed the CADESI-4 score outcome of the CAD-diagnosed dogs. The difference between diet cohorts was insignificant, with a weak worsening trend in the KD cohort (*p* = 0.104). There was a general trend in worsening of CADESI-4 scores found in both diet cohorts (for the KD: *n* = 9, μ = 18.3, σ = 13.8; for the RMBD: *n* = 11, μ = 6.9, σ = 6.5). The change in CADESI-4 scores did not result in a progression from mild to moderate CAD symptoms in any of the CAD-diagnosed canines however. In the serum samples of dogs from all dogs (*n* = 20), no significant weight and age differences between the KD and RMBD cohorts at the end of the diet intervention were detected. Results from the univariate analysis of CADESI-4, weight, and age across diet and disease cohorts are presented in Supplementary Tables 3A–E.

### Analysis Between of Diet and Atopy

**Figure 4:**
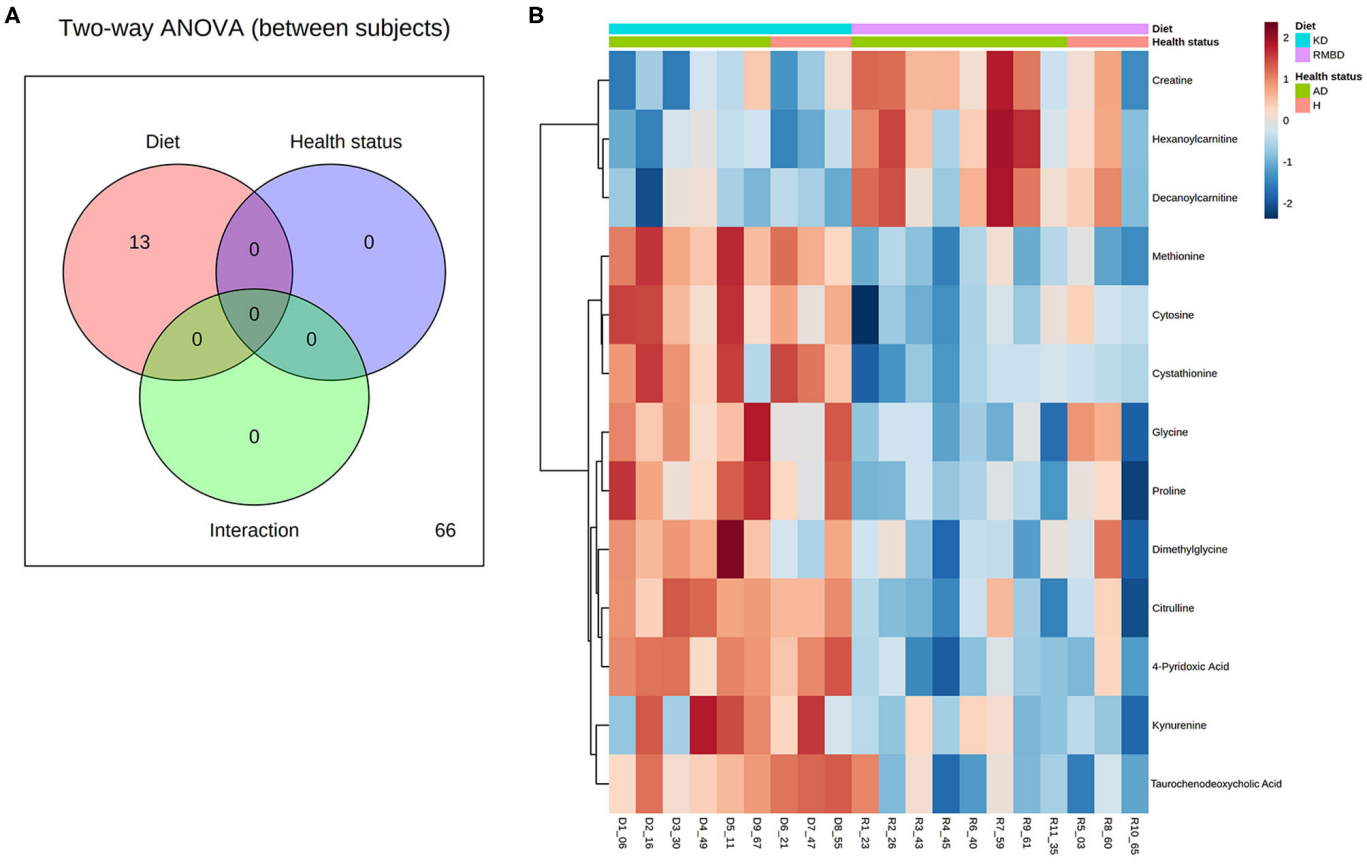
ANOVA of serum from all dogs at the end of diet intervention shows significant metabolite differences between diet cohorts, but not between health status cohorts. **(A)** An overview of how metabolite values differ significantly (FDR < 0.05) between diet cohorts (red) and health status cohorts (blue), as well as any significant interaction between them (green) for all dogs (*n* = 20) at end of diet intervention. **(B)** A heatmap illustrating significant metabolite concentration differences in the two-way ANOVA for CAD-diagnosed (*n* = 14) and healthy individuals (*n* = 6) (green and orange) and between the kibble diet (KD) (*n* = 9) and raw meat–based diet (RMBD) (*n* = 11) cohorts at the end of diet intervention.

**Figure 5:**
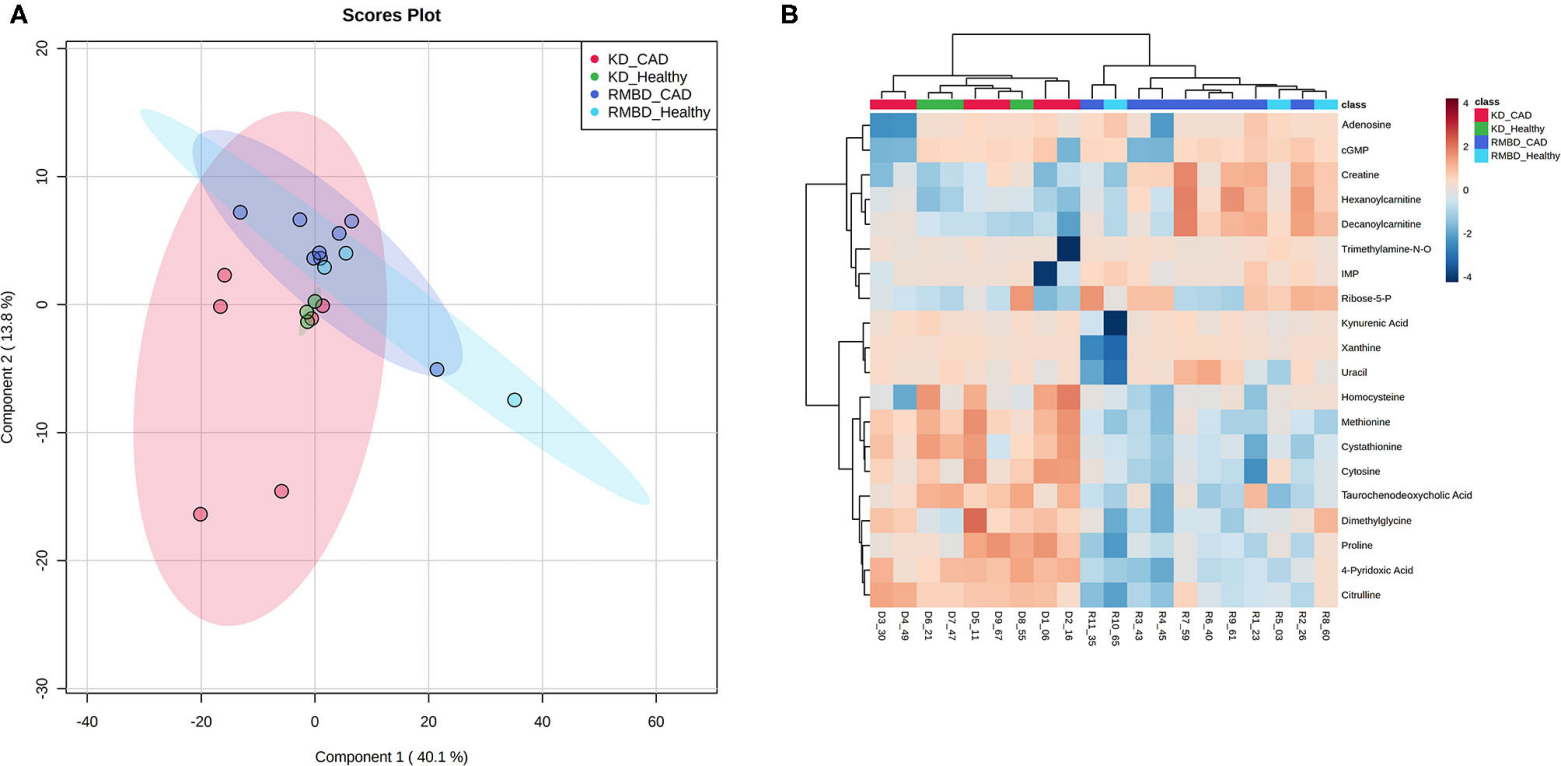
PLS-DA analysis of the diet cohorts and health status cohorts. **(A)** PLS-DA (partial least squares–discriminant analysis) plot of the first two components, displayed with 95% confidence intervals for each diet group (shaded regions of same color). **(B)** A PLS-DA VIP score heatmap visualization of the most important features (*n* = 20) across components (KD, kibble diet; RMBD, raw meat–based diet; CAD, canine atopic dermatitis).

In all the atopic dogs, no significant differences in CADESI-4 scores between diet cohorts were found at the diet intervention baseline, where the dogs' diets were mixed, or at the end of the diet intervention. The outcome of serum concentrations of all dogs (*n* = 20) at the end of the diet intervention were visualized as a two-way ANOVA between diet and atopy and their interactions (Figure 4A). Here, the RMBD and KD cohorts were classified as either healthy (healthy-RMBD, *n* = 3, healthy-KD, *n* = 3) or atopic (RMBD, *n* = 8, KD, *n* = 6). Metabolite values that differed significantly between either diet or health status cohorts, or their interaction, are presented in Supplementary Table 12. The significantly different metabolites between diet cohorts from the two-way ANOVA of the atopic and healthy canines were visualized with a heatmap (Figure 4B). To further address the separation of cohorts based on diet and health status, PLS-DA analysis was performed to see how the metabolite profiles differed between diet and health status cohorts (Figure 5A). The parameters of the model were calculated using the LOOCV approach and are shown in Supplementary Table 14B. Likely due to the low sample size, as well as the similarity between the CAD-diagnosed and healthy individuals serum metabolite concentrations, the predictability of the model calculated with R2 and its predictability when testing the model (Q2) were 0.277, which is relatively weak. Nevertheless, the model gives an indication toward how the healthy individuals in both diet groups were more closely clustered among themselves than the atopic individuals of either diet cohort. The top 20 VIP scores were visualized as a heatmap that looks at the top 20 metabolites across all components (Figure 5B), with which the diet cohorts could be separated, but that the health status cohorts (CAD-diagnosed and healthy) could not. Many of the metabolites found to be significantly different with the two-way ANOVA described above and the univariate analysis at end of the diet intervention (Table 3) were also found to have high VIP scores.

**Figure 6:**
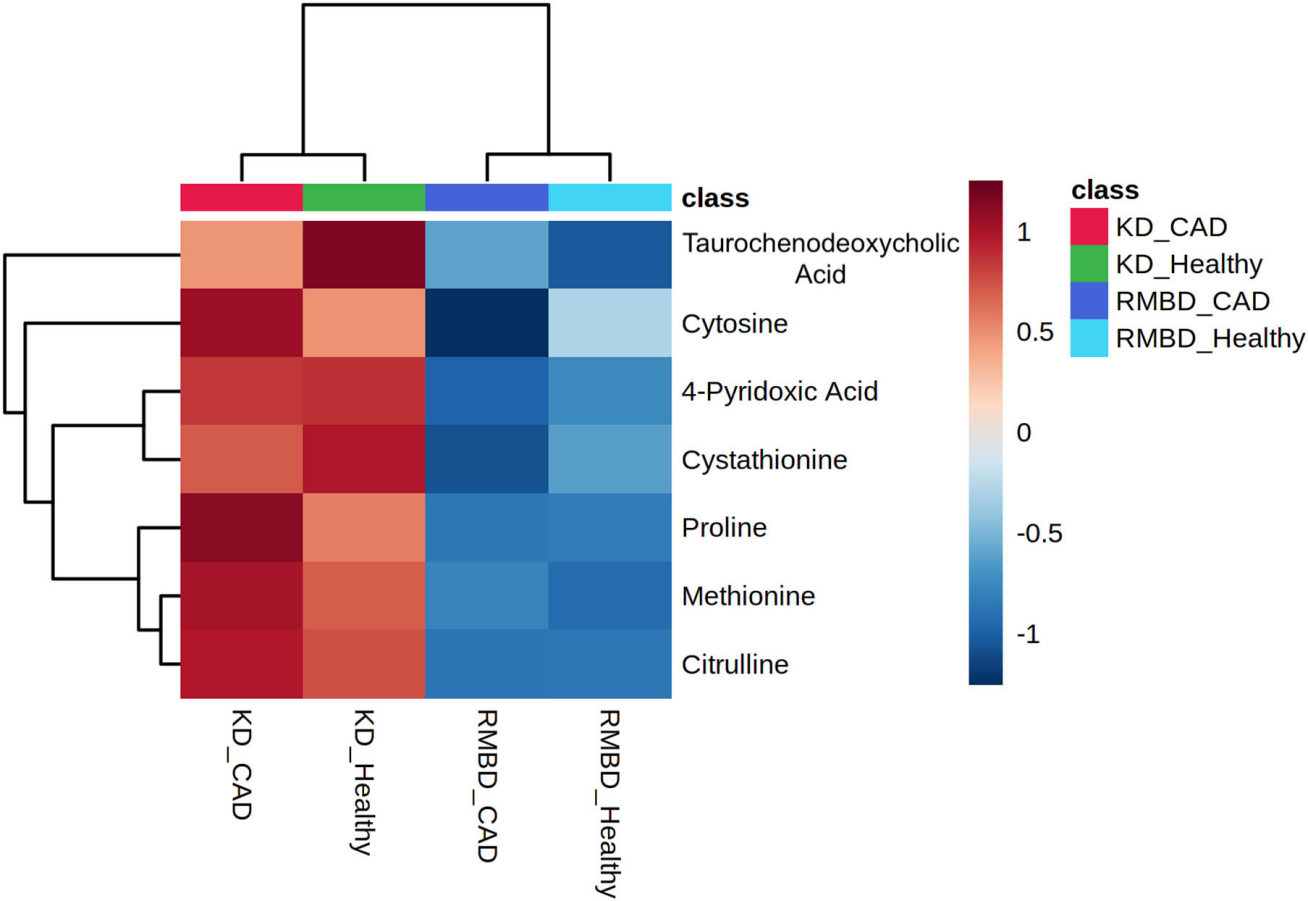
Fisher least significant difference (LSD) test to determine the significant differences between diet cohorts of healthy and atopic individuals. Significant differences between metabolite concentrations calculated with Fisher LSD test (KD, kibble diet; RMBD, raw meat–based diet; CAD, canine atopic dermatitis).

As a follow-up to the two-way ANOVA, an unprotected Fisher LSD test was used to compare how the metabolite concentrations at the end of the diet intervention differed between the four cohorts, i.e., the healthy and atopic dogs of both diet cohorts. The significant differences (FDR < 0.05) between these cohorts are presented in Figure 6 as group averages. The tabulated results are included in Supplementary Table 13.

## Discussion

### Diet Cohorts Readily Distinguished by Distinct Serum and Urine Metabolite Profiles

The two diets included in this study were remarkably different in terms of the types of raw ingredients used, their macronutrient and micronutrient composition, and their manufacturing methods. This suggests that the feeding of a particular diet could have a profound impact on metabolism, which in turn could have an effect on the dog's overall health and well-being. To our best knowledge, no data are available about the comparative study of blood and urine metabolomics in response to raw meat–based and KDs. Most of the metabolomics-based studies performed before 2015 are referred to in a review paper by Allaway (59). To date, only the study by Schmidt et al. (60) compares the differences between an RMBD and a KD using metabolic profiling. However, that study considers the fecal metabolome. The first study to evaluate health outcomes as a result of feeding commercial RMBDs was published in 2012 (61). The authors concluded that no undesirable changes occurred to either blood biogenic amine concentrations or skin and coat conditions in dogs fed the RMBDs in their study. Here, the major differences in metabolite concentrations observed between the diet cohorts could indicate impact on blood biochemistry, overall health, and the CAD condition are discussed in light of literature found regarding these topics.

There were higher concentrations of several of the carnitines, creatine, and creatinine in the serum of the RMBD cohort than in the KD cohort (all dogs, *n* = 20) (Table 3). This finding is likely reflected in the markedly higher meat content of the RMBD diet. Meat is the main dietary source of carnitines (62) and creatine (63). It is likely that the elevated creatinine concentrations in the RMBD cohort because creatine is the direct precursor of creatinine (64). Furthermore, carnitines play crucial roles in long-chain fatty acid transport for mitochondrial oxidation, which is to be expected of canines eating a fat-rich diet. Higher serum carnitine concentrations have been associated with antiaging effects in canines (65). The authors note that higher carnitine concentrations are associated with younger dogs, but they make no claims as to age-related health benefits (65).

The urea-cycle metabolites citrulline and proline were found in significantly lower serum concentrations in the RMBD cohort than in the KD cohort (Table 3). These metabolites are involved in urea production and ammonia recycling (66, 67). Citrulline is the direct precursor for arginine synthesis (68). Meat protein contains high amounts of both arginine (69) and creatine (63), where arginine, and subsequently citrulline, is required for creatine synthesis (70). As citrulline is used to accept the amino groups of excess amino acids from dietary protein (71), the higher protein content in the RMBD may explain this observation; i.e., less citrulline would be required by the KD-fed dogs, which possibly explains the higher concentrations observed in the KD cohort. Proline is found in especially high concentrations in collagen (72), an unexpected finding considering the likely higher collagen content in the RMBD.

The significantly higher serum concentrations of the nucleobase cytosine observed in all of the dogs of the KD cohort (Table 3), as well as urine concentrations in the batch 1 KD cohort (Supplementary Table 6) at the end of the diet intervention, are notable. To the best of our knowledge, no studies have investigated the relationships of diet between cytosine concentrations in blood and urine.

In blood serum of all dogs in the KD cohort, higher concentrations of the primary bile acid taurochenodeoxycholic acid could be seen after the diet intervention than in the RMBD cohort. Elevated concentrations of the downstream product of taurochenodeoxycholic acid, deoxycholic acid, has been implicated in colon tumorigenesis in both mice and humans (73, 74). Colon cancer is exceptionally high in canines (75), although the links to bile acid concentrations remain poorly understood. Although insignificant, taurocholic acid was also found in higher serum concentrations in the KD cohort relative to the RMBD cohort. It has been established that the composition of the microbiota throughout the canine gut is largely defined by the nutritional profile of dietary intake (76, 77). The microbiota composition modulates the amount and composition of nutrients that are able to pass through the gut endothelium, hence affecting blood serum biochemistry (78). Most studies on this topic have focused solely on fecal samples (79). Bile acid concentrations have been suggested to be sensitive to changes in gut microbiota composition. It has been reported that fecal bile acid concentrations increase in canines when fed an animal-based, high-fat, low-fiber diet (80). Elevated primary bile acid concentrations in blood have been shown to be a sign of elevated inflammation (81), especially with regard to the liver (82). No reference values regarding what levels lead to increased inflammation have been reported for canines (80).

Because of their toxicity, bile acid concentrations are tightly regulated in mice (83) and furthermore are usually increased as a response to increased fat digestion (84) as they function essentially as emulsifiers to improve fat absorption through the endothelium. Given the far greater amounts of fat present in the RMBD, this finding comes as a surprise. However, it should be noted that there were also large amounts of carbohydrate present in the high-fat, low-fiber diet in the study performed by O'Keefe et al. (84). As the RMBD has little to no carbohydrate, the energy metabolism of the canines was likely markedly different from the humans participating in the diet interventions of the O'Keefe et al. (84) study. The RMBD-fed dogs were possibly even ketogenic, i.e., causing a switch over to increased β-oxidation of fatty acids as a primary means for ATP production (85). It has been shown that even in the presence of high fat content, glucose is the preferred energy substrate in mammals (86). Canines fed a high-fat diet, in particular, one rich in medium-chain triglycerides (MCTs), even in the presence of high carbohydrate, have been reported to be ketogenic (87, 88). However, results from both studies performed by Packer et al. (87) and Law et al. (88) are questionable, as the authors neglected to measure or report ketone body values in the dogs and thereby establish whether the diets were ketogenic (89). Furthermore, ketone body production has been shown to rely on low levels of carbohydrate (90). Although MCTs are readily used for energy, even in the presence of carbohydrate (89), it does not necessarily switch the dog to a state of endogenous ketosis, i.e., where fat is the preferred metabolic energy source—the underlying assumption of a ketogenic diet (90). Ketogenic diets may affect serum bile acid concentrations in mice (91). In mammals, a switch over to ketogenic metabolism has major implications for altering glycolytic energy metabolism (92) and an increase in NADPH production, which is produced via the pentose phosphate pathway (93). In the RMBD cohort of batch 1 (*n* = 4) (Supplementary Table 7), a significantly higher level of ribose-5-phosphate was observed, indicating an upregulation of the pentose phosphate pathway (92) and subsequently a downregulation of glycolysis and upregulation of ketone body production. However, higher concentrations ribose-5-phosphate were not observed in the RMBD cohort of batch 2 (*n* = 7) or when observing all the dogs in the RMBD cohort (*n* = 11). The discrepancy between batches in itself merits further investigation. The finding in batch 1 may indicate that the RMBD was ketogenic, although to date no studies to our knowledge have considered the ketogenic properties of RMBDs, an area that merits further investigation.

**Figure 7:**
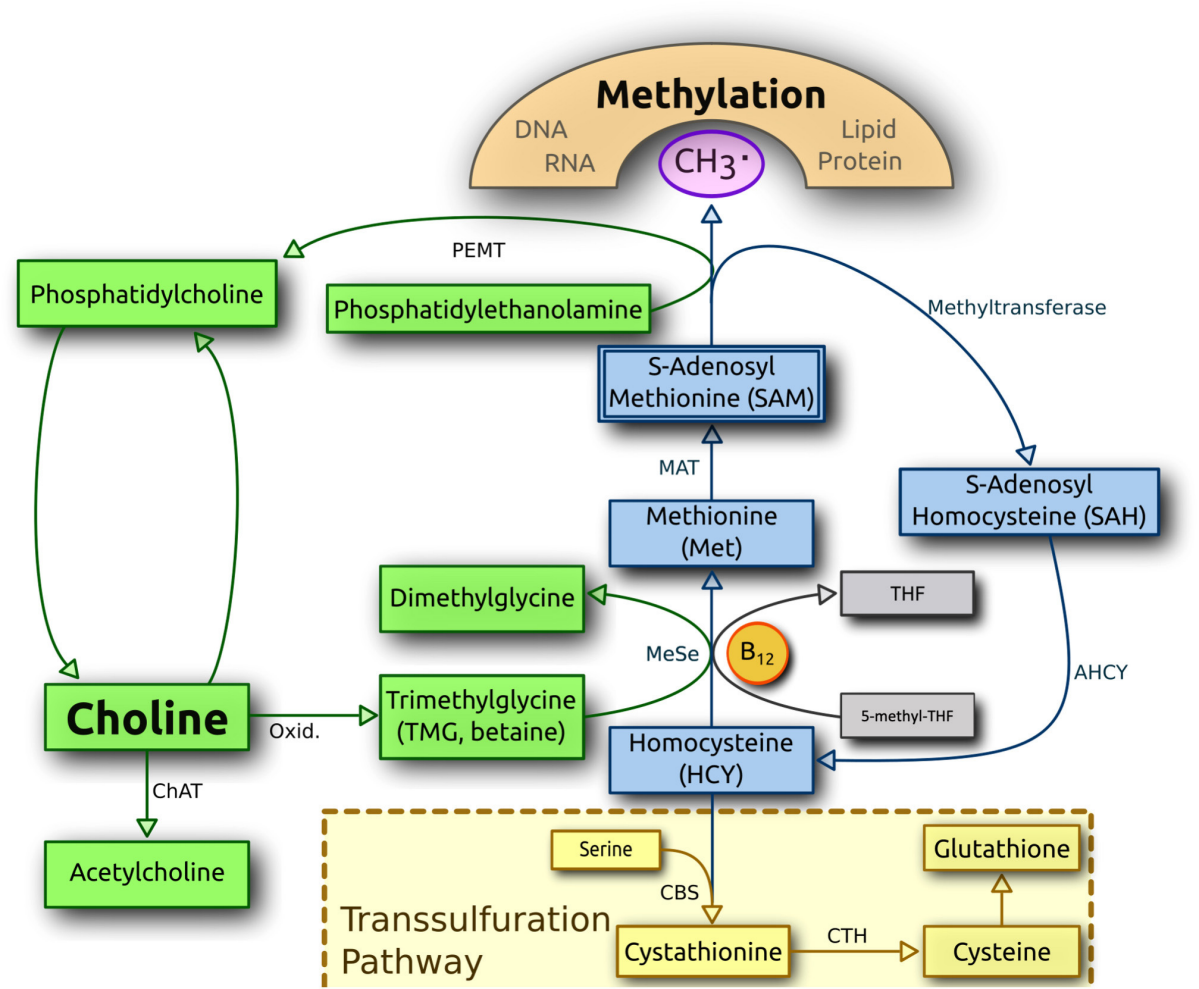
An overview of homocysteine metabolism and the transsulfuration pathway. CBS, cystathione-beta synthase; MeSe, methionine synthase; THF, tetrahydrofolate (Figure uploaded by radio89 and labeled for reuse. https://commons.wikimedia.org/wiki/File:Choline_metabolism-en.svg. Image modified to present all terms in English).

At the end of the diet intervention, all canines in the KD cohort had higher serum concentrations of the sulfur-containing amino acid methionine than the RMBD cohort (Table 3). The batch 1 KD cohort also had significantly higher urine methionine concentrations than the RMBD cohort (Supplementary Table 6). The serum of all canines in the KD cohort had higher levels of cystathionine. Both play important roles in homocysteine metabolism via the remethylation pathway, via the transsulfuration pathway, and via one-carbon pathway (94). The amino acid homocysteine is remethylated to methionine in a process dependent on vitamin B_12_ (B_12_) or is converted to cysteine via cystathionine in a vitamin B_6_-dependent process (94). A schematic representation of the methionine and transsulfuration pathways is represented in Figure 7. Serum methionine concentrations have been implicated in the outcomes of many long-term health studies in a vast selection of organisms (95). It has been shown that lower consumption and subsequent blood concentrations of this essential amino acid are associated with longevity across species (95, 96), as well as improved blood glucose tolerance in rats, lower levels of oxidative stress in mice (97), and a lower risk of developing cancers in both species (98, 99). The amount of food that dogs are fed may also affect dog health; however, this consideration falls beyond the scope of the present study. Elevated serum methionine concentration serves as an indicator of overfeeding as has been shown in mice (100). As there was considerably more meat-based protein present in the RMBD, it could be expected to be reflected as higher blood serum and urine concentrations of methionine in the RMBD cohort. However, the KD manufacturer apparently adds an unspecified amount of dl-methionine to the kibble (Supplementary Table 1A), which may in part explain this observation. Another explanation may be that canines in the KD cohort are actively eliminating or recycling greater concentrations of homocysteine than dogs fed the RMBD. In only the atopic dogs of the KD (*n* = 6) cohort, there is a trend of higher homocysteine concentrations vs. the atopic dogs of the RMBD (*n* = 8) cohort (Supplementary File 21, sheet 23). Concurrently, there is also a trend of higher urine homocysteine concentrations of CAD-diagnosed KD-fed dogs from batch 1 (*p* = 0.05714, FDR = 0.1934) (Supplementary File 21, sheet 24). Although insignificant, there is a trend of higher homocysteine concentrations in both urine and in the batch 1 KD cohort (Supplementary File 21, sheets 19 and 24). In a previously reported study regarding the hematology of the canines during the diet intervention (50), it was determined that the canines in the KD-fed cohort had elevated concentrations of blood serum B_12_ values. The significantly higher concentrations of methionine in the blood sera and urine of the batch 1 KD cohort (Table 3, Supplementary Table 6) and concurrently higher B_12_ serum concentrations (50) may be partially due to increased methionine synthase activity (94) as homocysteine is converted to methionine via this pathway (Figure 7). The higher concentrations of B_12_ comports with a higher methionine/homocysteine ratio as methylated B_12_ is converted into B_12_, i.e., as its methyl group is donated to homocysteine, turning it into methionine. In the data reported by Anturaniemi et al. (50), serum folate concentrations were also significantly higher in KD-fed dogs, which also plays a role in homocysteine clearance (94). In the present study, however, concentrations of folic acid, the acid form of folate, were not significantly different between diet cohorts for either urine or serum. There is a correlation between the amount of B_12_ in the food with serum B_12_ in dogs (101), indicating that B_12_ concentrations in dogs are tightly regulated, i.e., conserved in dogs fed a diet low B_12_. Furthermore, 4-pyridoxic acid, a downstream product of pyridine (B_6_), was also found in significantly higher concentrations in both the serum and urine of the KD cohorts. As B_6_ is the cofactor for cystathione beta-synthase, which converts homocysteine to cystathionine via the transsulfuration pathway (102) (Figure 7), this may indicate that this pathway is significantly upregulated in the KD diet. Cystathionine, the first metabolite produced as a result of homocysteine clearance via the transsulfuration pathway (94), was found in far higher concentrations in all dogs in the KD cohort, with a high fold-change difference compared to the RMBD cohort (Table 3). Finally, higher serum concentrations of dimethylglycine were observed in all dogs in the KD cohort (Table 3), and a trend of higher serum concentration of betaine was found in all dogs in the RMBD cohort (Supplementary Table 5), as well as in the urine of the batch 1 RMBD cohort (Supplementary Table 6) compared to the KD cohort. Playing important roles in one-carbon metabolism, and subsequently often discussed in the context of DNA methylation, betaine is converted to dimethylglycine as its methyl group is added to homocysteine, producing methionine (103) (Figure 7).

Elevated homocysteine levels are often discussed as risk factors for various canine pathologies, including cardiovascular disease (104), increased inflammation (105), and certain renal pathologies (106). In humans, elevated levels of plasma homocysteine have been associated with irritable bowel syndrome and cancer (107). Elevated homocysteine levels and subsequent clearance have long been known to be a risk marker for MetS in humans (108). To our knowledge, no studies have observed any direct correlation between atopy and elevated homocysteine or methionine blood serum concentrations. However, a higher prevalence of AD in offspring was observed in the offspring of women with elevated circulating levels of vitamins B_12_ and folate, and hence upregulation of the homocysteine pathway may be related (109). Homocysteine is highly toxic for dogs (110), and blood homocysteine concentrations are kept low, lying within a narrow concentration range (111). Studies on mice have shown that homocysteine concentrations are kept low even in the case where serum concentrations of methionine (100), as well as cystathionine (106), are significantly increased. We find a similar phenomenon in the present study. It should be noted that the blood homocysteine concentrations in the canines of both diet cohorts were no higher than those reported for healthy canines elsewhere (112, 113). The significantly higher blood serum and urine concentrations may indicate that more methionine was added to the diet than biologically necessary (4, 114). This may also be true of other metabolites found in significantly higher concentrations in both the serum and urine samples of batch 1 (Supplementary Table 11A, Supplementary Figure 2A), including 4-pyridoxic acid, which as discussed above is likely related to the significantly higher cystathionine concentrations observed in the KD cohort.

The atopic complex is still not fully understood in canines (115), nor its relationship to MetS. Previous studies in mice (116) and humans (117) have provided contradictory evidence, indicating that AD both may (116) or may not (117) be linked to MetS in mammals (26). Whether underlying lifestyle choices predispose risk for both MetS and AD, or whether the development of MetS increases the risk of developing AD or vice versa, is not fully understood (26). According to the evaluation of CAD severity at the end of the diet intervention, neither the KD nor the RMBD significantly changed the CADESI-4 score outcome of the CAD-diagnosed canines, although there was a trend of greater CADESI-4 worsening in the KD cohort (*p* = 0.219) (Supplementary Table 3C). There was a general trend in worsening of CADESI-4 scores found in both diet cohorts (for the RMBD = 6.9, σ = 6.5, for the KD, μ = 18.3, σ = 13.8) (Supplementary Tables 3D,E, respectively). In order to avoid interference from the seasonality of the disease, the diet trial was originally planned to take place during the late fall and winter months, when plant allergens known to exacerbate symptoms were not present. As discussed above, the trial had to be pushed forward, such that it ended when many plants had begun to bloom in Finland. It is likely that this delay caused the worsening of symptoms in both diet cohorts. There were disagreements between the owner-reported CAD diagnosis, which used the visual analog scale, and the dermatologist's diagnosis, which used the CADESI-4 scale. A metabolomics approach can potentially address and classify differing phenotypes of CAD, by combining “omics” with clinical and epidemiological data. However, in the present study, when considering the targeted metabolomic analysis that compared the atopic and healthy individuals, there were no significantly different metabolite concentrations at either the baseline or the end of the diet trial (Figure 4A). This suggests that diagnosing CAD by studying the blood serum with the targeted metabolites used in this study is also challenging.

A couple of studies looking at macronutrient preference among dogs served several food choices of varying macronutrient compositions *ad libitum* have indicated that several breeds of dogs are well-attuned to what they prefer and what their bodies require (17, 118). In the first study, the authors observed that several breeds of dogs adjusted to a preferred PFC macronutrient composition of 30%:63%:7% ME over a 7-day period (118), and another study observed that Harrier hound dogs adjusted to a PFC macronutrient ratio of 44%:52%:4% ME (17). The adequacy of diets for domesticated dogs, especially with regard to macronutrient composition, has been studied by comparing their diet with the diet of wolf (*Canis lupus*) populations (119). A meta-analysis of 41 studies that observed the wolf diet in Europe and North America concluded that the average wolf diet has a PFC of 54%:45%:1% ME (119). With the lack of carbohydrate and relatively high protein content, it resembles the RMBD used in our study (Table 1). This macronutrient ratio also resembles the ratio that the dogs in the two *ad libitum* studies mentioned above preferred (17). The ratio these breeds tend toward comports with current nutritional guidelines for dogs (120), which classify proteins and fats as essential and carbohydrates as non-essential. It remains unclear whether increased starch digestibility offers any advantage to dogs with regard to their health span or whether the artificial selection for improved tolerance toward a starch-rich diet may outweigh the predisposition for other illnesses. Both of these topics deserve further study.

### Strengths and Limitations of the Study

To our best knowledge this pilot study was the first ever to apply a serum and urine metabolomics-based approach to study how feeding canines a high-fat, moderate-protein, very low-carbohydrate RMBD affects serum and urine metabolite concentrations, as well as compare the outcome with the serum and urine metabolite profiles of dogs fed a moderate-fat, moderate-protein, high-carbohydrate KD. This targeted metabolomics approach offers quantitative and reliable data of blood serum and urine metabolite concentrations. Both urine and serum were analyzed simultaneously, giving insight into the relationships between the serum and urine media and diet. All dogs were pedigreed Staffordshire bull terriers. Their health status was diagnosed by a dermatologist using Favrot's criteria and the CADESI-4 scale to produce validated clinical scores.

As the present study focuses specifically on nutrition, there were no controls for quantitative markers for sleep, physical activity, or overall stress. Because of the high cost of analysis, the number of dogs that were used for the study was kept to a minimum of three dogs per cohort (KD-healthy, RMBD-healthy). Targeted metabolomic analysis of the serum samples collected from the dogs was performed in two batches. The ACQUITY UPLC/MS-MS instrument used for metabolomic analysis was serviced in between the analysis of the two batches, resulting in significantly different metabolite values between batches. Of the 102 metabolites targeted, a considerable amount had to be removed from the first batch analysis. Targeted analysis of the serum samples of the second batch went considerably better. Even so, many of the metabolites were unable to be used in the combined-batch analysis. Given the vast variety of metabolites circulating in both serum and urine media, it is clear in retrospect that numerous metabolites not studied were worthy of analysis. As discussed in *Design and Animals*, the postponed end of the diet intervention possibly allowed the introduction of undesired seasonal effects on CAD severity due to plant allergens. The study used more CAD-diagnosed than healthy dogs. Several dogs considered healthy prior to their official diagnosis by the dermatologist had to be reclassified as CAD sufferers. There were no metabolites that significantly differed between diet cohorts of the healthy individuals at the end of the diet intervention (KD-healthy *n* = 3, RMBD-healthy *n* = 3). This is likely due to the small sample size. The far fewer significant differences in metabolites between diet cohorts of batch 2 (Supplementary Table 8) may indicate that the underlying health status (CAD or healthy) had an impact on the results and may explain why the response to diet in the fully atopic cohort (batch 1) showed starker differences than for batch 2. Alternatively, this result may be an artifact due solely to the smaller sample size of batch 1.

## Conclusions

Three key differences were observed with regard to the effects of diet on the canine metabolite profiles studied. First, there were markedly higher levels of carnitines and related compounds in canines fed the RMBD. Additionally higher levels of nitrogen excretion were indicated, also a result of the diet's high meat content. Second, the KD-fed cohort showed elevated bile acid concentrations that have a condition implicated, for example, in colon tumorigenesis in mice and humans. In addition to reflecting the macronutrient profile, it may also implicate a change in the gut microbiota composition. Further study is needed to confirm this. Third, there were higher concentrations of sulfur-containing compounds such as methionine and cystathionine, as well as compounds related to their metabolism, in the serum and urine of KD-fed dogs. Higher serum concentrations of these compounds are associated with increased inflammation in mammals. Furthermore, lower serum methionine concentrations as seen in the RMBD cohort have long been established as a marker associated with long life span and are generally considered beneficial for metabolic health. The latter two differences suggest that the KD may be less beneficial to the metabolic health of canines as metabolite concentrations that have been previously implicated in various pathologies were found in higher concentrations than in the RMBD-fed dogs. Given the limitations of the present study, however, such speculation requires further study to establish causality. Given the challenge of identifying CAD at the serum metabolite level, addressing and classifying differing phenotypes of CAD may be beyond the scope of a targeted metabolomics approach. Future studies will likely require both a larger set of metabolites to be targeted and larger sample cohorts. In summary, this experiment sought to clarify how nutrition may relate to CAD, as well as determine whether the impact of different diets could be seen on the metabolite level. While these topics are still novel for canine studies, the use of diet as a form of health maintenance, a notion that has gained popularity in recent years, will eventually be substantiated or rejected with quantitative clinical data.

## Epilogue

A growing movement based on lay publications and anecdotal evidence regarding canine nutrition asserts that a diet consisting of raw meats lowers the risk of disease in canines (13, 15, 16). Diet as a form of disease prevention has become popular in human research but has yet to be adopted by the mainstream veterinary science community. The health maintenance chapter in the authoritative compendium *Small Animal Clinical Nutrition* (121) begins with the quote: “To ward off disease or recover health, people as a rule find it easier to depend on healers than to attempt the more difficult task of living wisely.” Many dog owners want to take preventive measures to ward off disease in their pets, yet must place trust in veterinarians who may be unfamiliar with preventive measures to improve health span. To overcome the anecdotal nature of the discussion, scientific evidence can help in understanding the role of diet in promoting dog health.

## Data Availability Statement

Being funded by commercial sources has not altered our adherence to Frontiers policies on sharing data and materials. While the data is still used for graduation work, it will be disclosed later. However, for research purposes the data can be obtained upon request from the authors: anna.hielm-bjorkman@helsinki.fi.

## Ethics Statement

Owners provided informed written consent for inclusion of their dogs in the study. The protocol was also approved by the Animal Experiment Board in Finland (ELLA) (permit number: ESAVI/3244/04.10.07/2013).

## Author Contributions

AH-B and JA contributed conception and design of the study. RM and AH-B organized the database. VV and JN performed the metabolomic analysis. RM did the statistical analysis and wrote the first draft of the manuscript. RM, AH-B, JA, VV, and SB-M wrote sections of the manuscript. All authors contributed to manuscript revision and approval of the submitted version.

## Conflict of Interest

The authors declare that the research was conducted in the absence of any commercial or financial relationships that could be construed as a potential conflict of interest.

## Abbreviations

KD: kibble diet
RMBD: raw meat–based diet
%ME: percent metabolizable energy
PFC: Protein: fat: carbohydrate
CAD: canine atopic dermatitis
AD: Atopic dermatitis
MetS: Metabolic syndrome
CADESI(-4): Canine Atopic Dermatitis Extent and Severity.
