# Supplementary material for: Targeted Metabolomics With Ultraperformance Liquid Chromatography–Mass Spectrometry (UPLC-MS) Highlights Metabolic Differences in Healthy and Atopic Staffordshire Bull Terriers Fed Two Different Diets, A Pilot Study
Source: Front Vet Sci. 2020 Oct 27;7:554296. doi: 10.3389/fvets.2020.554296 (PMC7653775; doi:10.3389/fvets.2020.554296)
Supplement: Supplementary file 4 [file Data_Sheet_1.ZIP › Moore et al_Supplementary files_Analysis reports/Supplementary file 18- Analysis_Report(Batch 1 Serum + Urine Meta-analysis.pdf]

Metabolomic Data Analysis with MetaboAnalyst 4.0

Name: guest2402318957156762091

April 21, 2020

1 Background

The combination of multiple independent metabolomics studies investigating the same condition in similar populations, which is often termed horizontal integration, or metabolomic meta-analysis. The aim of metabolomic meta-analysis is to leverage the collective power of multiple studies to overcome potential noise, bias, and small effect sizes to improve the precision in identifying true patterns within data. Specifically, biomarker identification remains a large area of research in metabolomics, and their validation is challenging due to inconsistencies in identified biomarkers amongst similar experiments. Performing meta-analysis across similar studies will thereby increase the sample size and the power to identify robust and precise biomarkers of disease. The aim of the Meta-Analysis module for the integration of individual metabolomic studies to identify consistent and robust biomarkers of disease. This module supports three methods for performing meta-analysis: 1) Combining p-values, 2) Vote counting, and 3) Direct merging of data into a mega-dataset.

2 Meta-Analysis Overview

The Meta-Analysis module consists of six steps: 1) uploading the individual datasets; 2) data processing of each individual dataset, however it is suggested that the data-processing steps are consistent amongst the studies; 3) differential expression analysis of individual datasets; 4) data integrity check prior to meta-analysis ; 5) selection of the statistical method for meta-analysis, and 6) visualization of results as a Venn diagram to view all possible combinations of shared features between the datasets.

3 Data Input

The Meta-Analysis module accepts individual datasets which must be prepared by users prior to being uploaded. In general, the datasets must have been collected under comparable experimental conditions/share the same hypothesis or have the same mechanistic underpinnings. At the moment, the module only supports two-group comparisons (ex: control vs disease). Further, the module accepts either a compound concentration table, spectral binned data, or a peak intensity table. The format of the data must be specified, identifying whether the samples are in rows or columns, or may either be .csv or .txt files.

3.0.1 Sanity Check

Before data analysis, a sanity check is performed to make sure that all of the necessary information has been collected. The class labels must be present and must contain only two classes. If the samples are paired, the class label must be from -n/2 to -1 for one group, and 1 to n/2 for the second group (n is the sample number and must be an even number). Class labels with the same absolute value are assumed to be pairs. Compound concentration or peak intensity values must all be non-negative numbers. By

default, all missing values, zeros and negative values will be replaced by the half of the minimum positive value found within the data (see next section).

3.0.2 Normalization of Individual Data

Before differential expression analysis, datasets may be normalized using Log2 transformation. Additionally, users may choose to auto-scale their data. Log2 transformation. No data autoscaling was performed.

3.0.3 Differential Expression Analysis of Individual Data

Before meta-analysis, differential expression analysis using linear models (Limma) may be performed for exploratory analysis. Here, users must specify the p-value (FDR) cut-off and the fold-change (FC) cutoff.P value cutoff: 0.05 Fold-Change cutoff: 0 Number of significant features: 7 Number of non-significant features: 73

3.1 Data Integrity Check

Before meta-analysis, one final data integrity check is performed to ensure meta-data are consistent between datasets and that there are at least more than 25 percent common features between the collective datasets.

4 Meta-Analysis Output

After the data has passed the final integrity check, users have the option to select one of three methods to perform meta-analysis: 1) Combining p-values, 2) vote counting, or 3) directly merging the datasets into a mega-dataset.

4.1 Combining P-Values

Calculating and combining p-values for the meta-analysis of microarray studies has been a long standing method and now we apply it to metabolomics studies. It includes two popular approaches, the Fisher's method and the Stouffer's method, which have similar levels of performance and are generally interpreted as larger scores reflecting greater differential abundance. The main difference between the two methods are weights (which are based on sample size), which are used in the Stouffer's method but not used in the Fisher's method. It should be noted that larger sample sizes do not warrant larger weights, as study quality can be variable. Further, users should use the Stouffer's method only when all studies are of similar quality.

4.2 Vote Counting

Vote counting is considered the most simple yet most intuitive method for meta-analysis. Here, significant features are selected based on a selected criteria (i.e. an adjusted p-value <0.05 and the same direction of FC) for each dataset. The votes are then calculated for each feature by counting the total of number of times a feature is significant across all included datasets. However, this method is statistically inefficient and should be considered the last resort in situations where other methods to perform meta-analysis cannot be applied.

4.3 Direct Merging

The final method of meta-analysis is the direct merging of individual data into a mega-dataset, which results in an analysis of that mega-dataset as if the individual data were derived from the same experiment. This method thereby ignores any inherent bias and heterogeneity between the different data. Because of this, there exists several confounders such as different experimental protocols, technical platforms, and raw data processing procedures that can mask true underlying differences. It is therefore highly suggested that this approach be used only when individual data are very similar (i.e. from the same lab, same platform, without batch effects).

P-value combination was the selected method to perform meta-analysis. The method of p-value combination used is: fisher The p-value significance threshold is: 0.05

Table 1: Predicted top-ranking features from meta-analysis

	V1	V2	CombinedTstat	CombinedPval
Methionine	0.00	0.01	-38.17	0.00
Cytosine	0.01	0.01	-33.92	0.00
Creatine	0.01	0.01	32.92	0.00
4-Pyridoxic Acid	0.01	0.01	-30.42	0.00
Betaine	0.01	0.05	25.68	0.00
Cystathionine	0.27	0.01	-25.04	0.00
Ribose-5-P	0.31	0.01	22.74	0.00
Alanine	0.01	0.85	19.48	0.00
Citrulline	0.77	0.01	-19.44	0.00
Hydroxyproline	0.01	0.91	17.88	0.01
Uracil	0.01	0.89	15.93	0.02
GABA	0.01	0.94	-15.64	0.02
Homocysteine	0.09	0.41	-14.81	0.02
Hexanoylcarnitine	0.19	0.15	14.92	0.02
1-methylhistamine	0.09	0.62	13.78	0.03
Folic Acid	0.03	0.95	13.25	0.04
Threonine	0.08	0.64	13.48	0.04
Inosine	0.20	0.26	13.29	0.04
Adenine	0.03	0.73	12.23	0.04
Proline	0.91	0.06	-12.31	0.04
Lysine	0.19	0.37	12.53	0.04
Nicotinic Acid	0.10	0.64	12.46	0.04
Octanoylcarnitine	0.21	0.31	12.61	0.04
Decanoylcarnitine	0.20	0.37	12.32	0.04
Carnosine	0.08	0.82	12.10	0.04

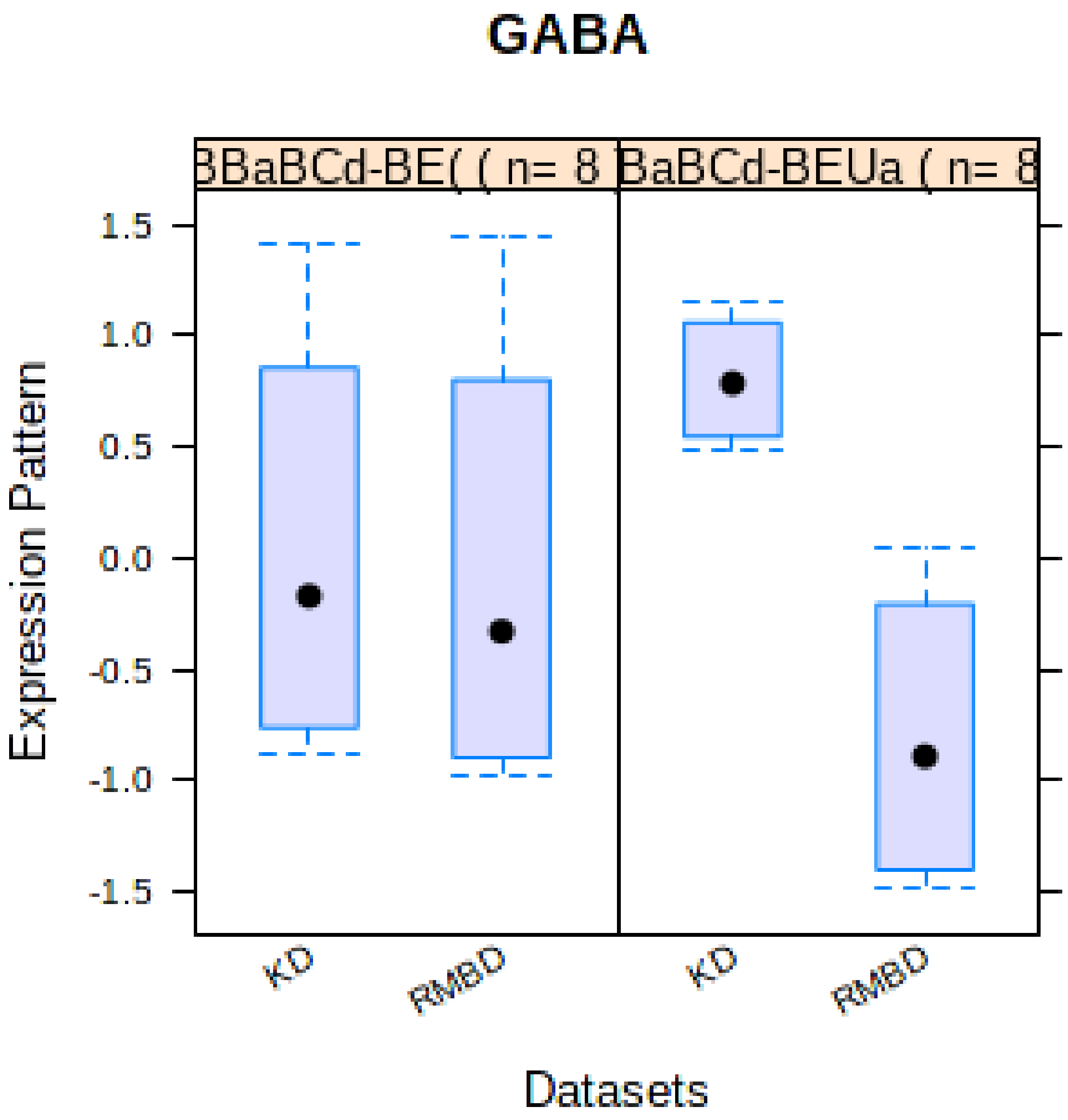

Figure 1: Box plot of the expression pattern of the selected feature between the two experimental groups across all studies. The expression pattern is on the y-axis, and the group labels are on the x-axis. The median expression for the feature is indicated with a black dot in the centre of the boxplot.
Selected feature: GABA

5 Appendix: R Command History

```
 [1] "mSet<-InitDataObjects(\"conc\", \"metadata\", FALSE)"
 [2] "mSet<-ReadIndData(mSet, \"B1, B2, and B1&B2 Combined datasets_Feb2019 - B1 End Urine_creatinine
 [3] "mSet<-SanityCheckIndData(mSet, \"B1, B2, and B1&B2 Combined datasets_Feb2019 - B1 End Urine_cre
 [4] "mSet<-ReadIndData(mSet, \"B1, B2, and B1&B2 Combined datasets_Feb2019 - B1 END (serum)(KNN).csv
 [5] "mSet<-SanityCheckIndData(mSet, \"B1, B2, and B1&B2 Combined datasets_Feb2019 - B1 END (serum)(K
 [6] "mSet<-PerformIndNormalization(mSet, \"B1, B2, and B1&B2 Combined datasets_Feb2019 - B1 End Urin
 [7] "mSet<-PerformIndNormalization(mSet, \"B1, B2, and B1&B2 Combined datasets_Feb2019 - B1 END (ser
 [8] "mSet<-PerformLimmaDE(mSet, \"B1, B2, and B1&B2 Combined datasets_Feb2019 - B1 End Urine_creatin
 [9] "mSet<-PerformLimmaDE(mSet, \"B1, B2, and B1&B2 Combined datasets_Feb2019 - B1 END (serum)(KNN)
[10] "mSet<-CheckMetaDataConsistency(mSet, F);"
[11] "mSet<-PerformPvalCombination(mSet, \"fisher\", 0.05)"
[12] "mSet<-GetMetaResultMatrix(mSet, \"fc\")"
[13] "mSet<-GetMetaResultMatrix(mSet, \"pval\")"
[14] "mSet<-PlotSelectedFeature(mSet, \"Methionine\")"
[15] "mSet<-PlotSelectedFeature(mSet, \"Cytosine\")"
[16] "mSet<-PlotSelectedFeature(mSet, \"Creatine\")"
[17] "mSet<-PlotSelectedFeature(mSet, \"Alanine\")"
[18] "mSet<-PlotSelectedFeature(mSet, \"Betaine\")"
[19] "mSet<-PlotSelectedFeature(mSet, \"4-Pyridoxic Acid\")"
[20] "mSet<-PlotSelectedFeature(mSet, \"Creatine\")"
[21] "mSet<-PlotSelectedFeature(mSet, \"Cytosine\")"
[22] "mSet<-PlotSelectedFeature(mSet, \"Methionine\")"
[23] "mSet<-PlotSelectedFeature(mSet, \"Cystathionine\")"
[24] "mSet<-PlotSelectedFeature(mSet, \"Ribose-5-P\")"
[25] "mSet<-PlotSelectedFeature(mSet, \"Hydroxyproline\")"
[26] "mSet<-PlotSelectedFeature(mSet, \"Uracil\")"
[27] "mSet<-PlotSelectedFeature(mSet, \"Adenine\")"
[28] "mSet<-PlotSelectedFeature(mSet, \"Folic Acid\")"
[29] "mSet<-PlotSelectedFeature(mSet, \"Citrulline\")"
[30] "mSet<-PlotSelectedFeature(mSet, \"Ribose-5-P\")"
[31] "mSet<-PlotSelectedFeature(mSet, \"Citrulline\")"
[32] "mSet<-PlotSelectedFeature(mSet, \"Alanine\")"
[33] "mSet<-SaveTransformedData(mSet)"
[34] "mSet<-PreparePDFReport(mSet, \"guest2402318957156762091\")\n"
[35] "mSet<-GetMetaResultMatrix(mSet, \"fc\")"
[36] "mSet<-GetMetaResultMatrix(mSet, \"pval\")"
[37] "mSet<-PlotSelectedFeature(mSet, \"GABA\")"
[38] "mSet<-SaveTransformedData(mSet)"
[39] "mSet<-PreparePDFReport(mSet, \"guest2402318957156762091\")\n"
```

The report was generated on Tue Apr 21 07:25:55 2020 with R version 3.6.3 (2020-02-29).
