# Supplementary material for: Targeted Metabolomics With Ultraperformance Liquid Chromatography–Mass Spectrometry (UPLC-MS) Highlights Metabolic Differences in Healthy and Atopic Staffordshire Bull Terriers Fed Two Different Diets, A Pilot Study
Source: Front Vet Sci. 2020 Oct 27;7:554296. doi: 10.3389/fvets.2020.554296 (PMC7653775; doi:10.3389/fvets.2020.554296)
Supplement: Supplementary file 4 [file Data_Sheet_1.ZIP › Moore et al_Supplementary files_Analysis reports/Supplementary file 11- Analysis_Report(B1 serum and urine separate factors).pdf]

Metabolomic Data Analysis with MetaboAnalyst 4.0

Name: guest18175290510364713201

April 19, 2020

1 Data Upload and Integrity Checking

1.1 Upload your data

For two-factor and time-series data, MetaboAnalyst accepts data uploaded in comma separated values (.csv) format. Samples can be in rows or columns. The two factor labels must follow immediately after the sample names. For time-series data, the time points group must be named as **Time**. In addition, the samples collected from the same subject at different time points should be consecutive and ordered by the time points. Users need to specify the data types when uploading their data in order for MetaboAnalyst to select the correct algorithm to process them. Table 1 summarizes the result of the data checking steps.

`Samples are in columns and features in rows. The uploaded file is in comma separated values (.csv) format. The uploaded data file contains 16 (samples) by 72 (compounds) data matrix.`

Table 1: Summary of data processing results

	Features (positive)	Missing/Zero	Features (processed)
D1_06	72	0	72
D2_16	72	0	72
D4_49	72	0	72
D3_30	72	0	72
D1_06.1	72	0	72
D2_16.1	72	0	72
D3_30.1	72	0	72
D4_49.1	72	0	72
R3_43	72	0	72
R4_45	72	0	72
R1_23	72	0	72
R2_26	72	0	72
R1_23.1	72	0	72
R2_26.1	72	0	72
R3_43.1	72	0	72
R4_45.1	72	0	72

1.2 Data Integrity Check

Before data analysis, a data integrity check is performed to make sure that all the necessary information has been collected. Compound concentration or peak intensity values should all be non-negative numbers. By default, all missing values, zeros and negative values will be replaced by the half of the minimum positive value found within the data (detection limits).

`Samples are in columns and features in rows. The uploaded file is in comma separated values (.csv) format. The uploaded data file contains 16 (samples) by 72 (compounds) data matrix. The data is not time-series data. 2 groups were detected in samples for factor Diet 2 groups were detected in samples for factor Sample type Only English letters, numbers, underscore, hyphen and forward slash (/) are allowed. Other special characters or punctuations (if any) will be stripped off. All data values are numeric. A total of 0 (0%) missing values were detected. By default, these values will be replaced by a small value.`

1.3 Data Normalization

The data is stored as a table with one sample per row and one variable (bin/peak/metabolite) per column. The normalization procedures implemented below are grouped into four categories. Sample specific normalization allows users to manually adjust concentrations based on biological inputs (i.e. volume, mass); row-wise normalization allows general-purpose adjustment for differences among samples; data transformation and scaling are two different approaches to make features more comparable. You can use one or combine both to achieve better results.

The normalization consists of the following options:

1. Row-wise procedures:
   - Sample specific normalization (i.e. normalize by dry weight, volume)
   - Normalization by the sum
   - Normalization by the sample median
   - Normalization by a reference sample (probabilistic quotient normalization)[1]
   - Normalization by a pooled or average sample from a particular group
   - Normalization by a reference feature (i.e. creatinine, internal control)
   - Quantile normalization
2. Data transformation :
   - Generalized log transformation (glog 2)
   - Cube root transformation
3. Data scaling:
   - Mean centering (mean-centered only)
   - Auto scaling (mean-centered and divided by standard deviation of each variable)
   - Pareto scaling (mean-centered and divided by the square root of standard deviation of each variable)
   - Range scaling (mean-centered and divided by the value range of each variable)

Figure 1 shows the effects before and after normalization.

[1] Dieterle F, Ross A, Schlotterbeck G, Senn H. *Probabilistic quotient normalization as robust method to account for dilution of complex biological mixtures. Application in 1H NMR metabonomics*, 2006, Anal Chem 78 (13);4281 - 4290

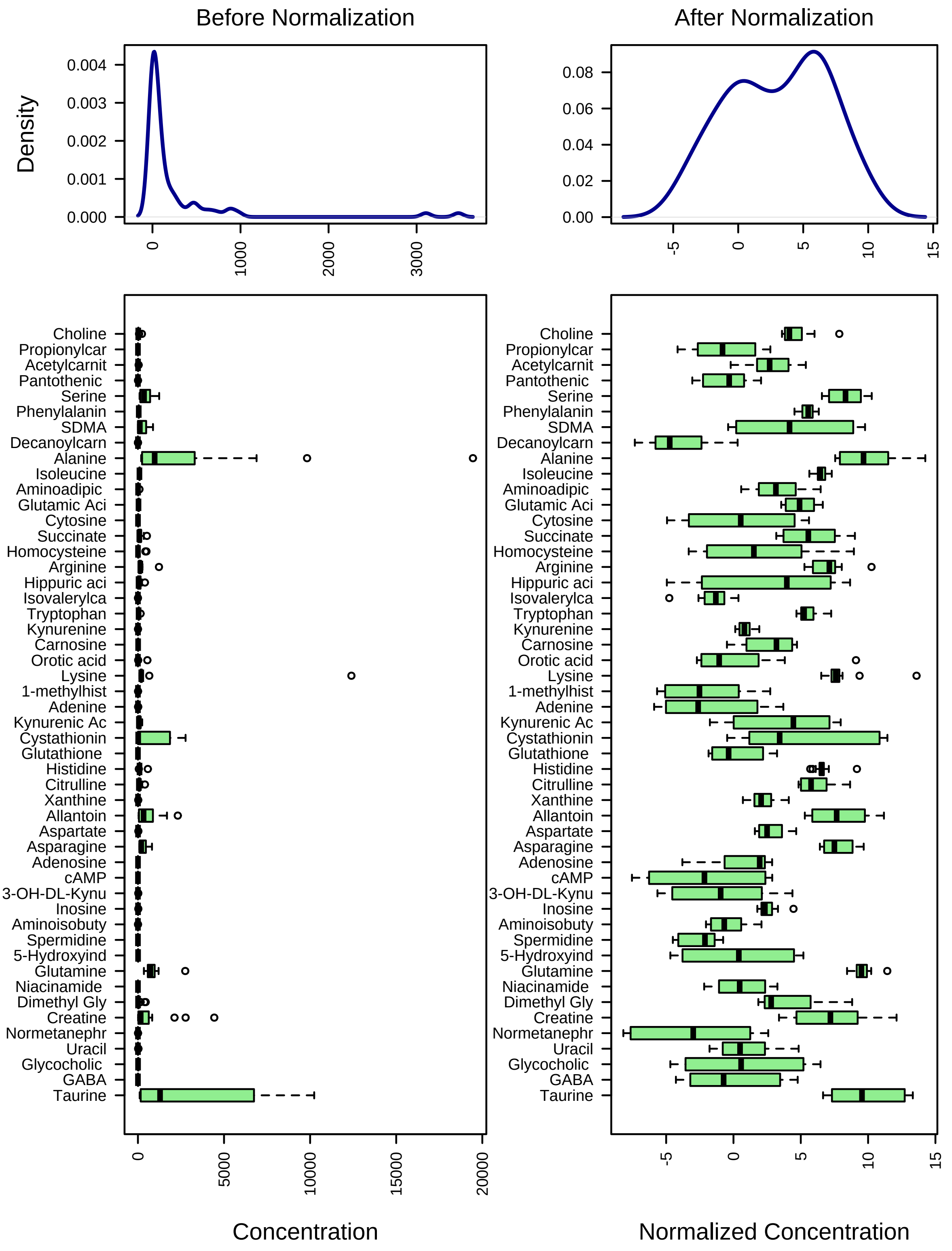

Figure 1: Box plots and kernel density plots before and after normalization. The boxplots show at most 50 features due to space limit. The density plots are based on all samples. Selected methods : Row-wise normalization: N/A; Data transformation: Log Normalization; Data scaling: N/A.

2 Statistical and Machine Learning Data Analysis

For two-factor and time-series data, MetaboAnalyst offers several carefully selected methods for general two-factor and time-series data analysis. They include:

- Data overview:
  - Interactive Principal Component Analysis (iPCA)
  - Two-way Heatmap clustering and visualization
- Univariate method:
  - Two-way between/within-subjects ANOVA
- Multivariate approaches
  - ANOVA-Simultaneous Component Analysis (ASCA)
  - Multivariate Empirical Bayes Analysis (MEBA)

`Please note: MEBA is only applicable to time-series data analyais.`

2.1 Two-way Heatmap Visualization

The heatmap provides direct visualization of all data points in the form of colors squares. The color spectrum intuitively indicates the higher or lower values. Users can choose different clustering algorithms or distance measures to cluster the variables. The samples are ordered by the two factors with default the first factor used for primary ordering. Users can choose to switch the order.

Figure 2 shows the clustering result in the form of a heatmap.

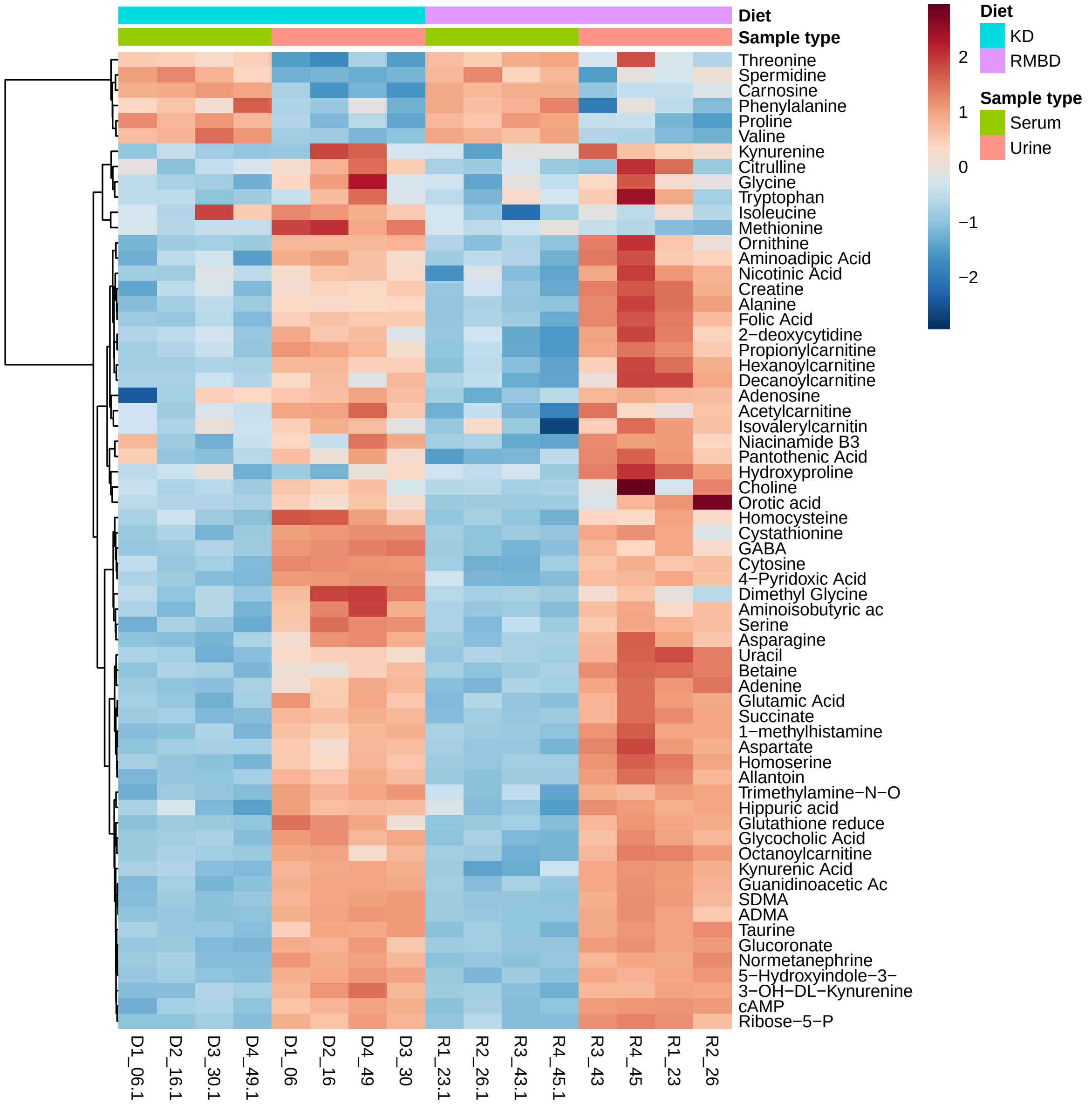

Figure 2: Clustering result shown as heatmap (distance measure using `euclidean`, and clustering algorithm using `ward.D`).

2.2 Univariate Analysis

Univariate analysis methods are the most common methods used for exploratory data analysis. For two-factor data, the basic approach is two-way ANOVA. There are two options - between-subjects ANOVA and within-subjects ANOVA. When samples are all from independent subjects (i.e. general two-way ANOVA), the between-subjects option should be selected. However, time series data contains samples measured from the same subjects from different time points. Therefore within-subjects ANOVA should be used.

Figure 3 shows the important features identified by ANOVA analysis. Table 2 shows the details of these features;

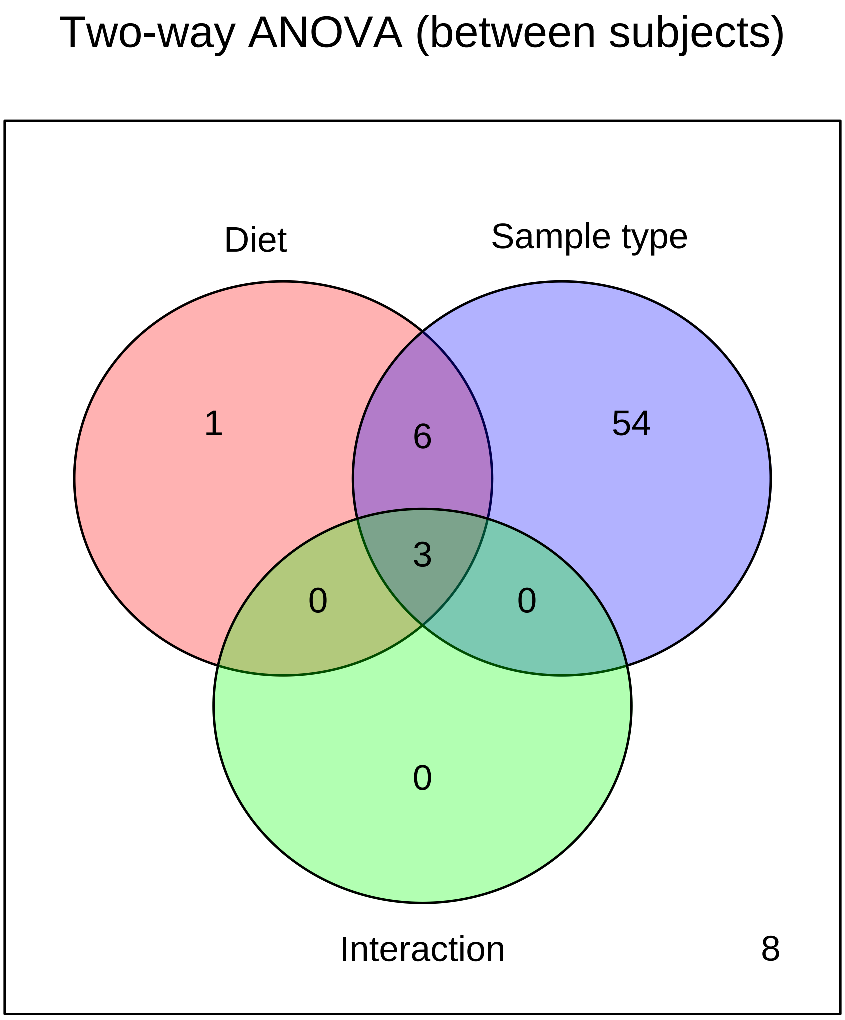

Figure 3: Plot of important features selected by two-way ANOVA.

Table 2: Top 50 features identified by Significant features identified by advanced ANOVA

	Compounds	Diet(F.val)	Diet(raw.p)	Diet(adj.p)	Sample type(F.val)	Sample type(raw.p)	Sample type(adj.p)
1	Methionine	44.818	2.213e-05	0.0015934	19.16	0.00090078	0.001201
2	Betaine	28.626	0.00017352	0.0062466	266.6	1.4679e-09	6.2172e-09
3	GABA	20.873	0.00064499	0.010867	372.36	2.1175e-10	1.386e-09
4	Uracil	20.825	0.00065088	0.010867	171.5	1.8162e-08	4.4057e-08
5	Homoserine	19.269	0.00088121	0.010867	359.23	2.6101e-10	1.4456e-09
6	Alanine	19.133	0.00090557	0.010867	225.31	3.8559e-09	1.3881e-08
7	Hydroxyproline	14.724	0.0023643	0.024318	16.792	0.0014783	0.0019007
8	Isoleucine	13.312	0.0033352	0.030016	3.5614	0.083556	0.094001
9	Cytosine	12.115	0.0045408	0.034762	365.69	2.3529e-10	1.4117e-09
10	Dimethyl Glycine	11.885	0.0048281	0.034762	56.335	7.1791e-06	1.2021e-05
11	Threonine	9.5123	0.0094638	0.061945	15.953	0.0017806	0.0022104
12	1-methylhistamine	8.0007	0.015217	0.091299	283.74	1.0247e-09	4.6113e-09
13	Homocysteine	7.4509	0.018279	0.10124	88.932	6.7237e-07	1.2413e-06
14	Glucoronate	7.0661	0.02086	0.10452	778.8	2.7724e-12	6.2552e-11
15	Adenine	6.943	0.021776	0.10452	220.97	4.3096e-09	1.4776e-08
16	Allantoin	6.3685	0.026732	0.1203	404.68	1.3032e-10	1.0425e-09
17	Carnosine	6.0543	0.030008	0.12709	180.1	1.378e-08	3.6746e-08
18	Creatine	5.7027	0.034258	0.13616	79.691	1.2031e-06	2.1128e-06
19	Acetylcarnitine	5.5782	0.035932	0.13616	46.709	1.812e-05	2.718e-05
20	Folic Acid	5.4206	0.038193	0.1375	171.17	1.8357e-08	4.4057e-08
21	Succinate	5.1131	0.043116	0.14313	390.26	1.6106e-10	1.1596e-09
22	Aspartate	5.0775	0.043734	0.14313	174.42	1.6511e-08	4.2458e-08
23	Spermidine	2.6845	0.12726	0.32972	54.014	8.8609e-06	1.45e-05
24	4-Pyridoxic Acid	2.6005	0.1328	0.32972	249.87	2.1311e-09	8.5244e-09
25	Aminoisobutyric acid	2.1961	0.16414	0.38485	103.27	3.0083e-07	6.1886e-07
26	Ribose-5-P	2.0955	0.17335	0.39005	333.19	4.0424e-10	1.9404e-09
27	cAMP	1.9986	0.18286	0.39272	485.8	4.4731e-11	4.6009e-10
28	Guanidinoacetic Acid	1.9733	0.18545	0.39272	749.61	3.4751e-12	6.2552e-11
29	Cystathionine	1.545	0.23762	0.47523	125.13	1.0532e-07	2.2979e-07
30	Glutamic Acid	1.1215	0.31045	0.59365	207.32	6.1991e-09	1.8597e-08
31	Glycocholic Acid	1.1077	0.31332	0.59365	343.03	3.4134e-10	1.7555e-09
32	3-OH-DL-Kynurenine	1.0039	0.33615	0.62058	244.56	2.4108e-09	9.1355e-09
33	Orotic acid	0.77618	0.39561	0.68913	22.6	0.00046893	0.00064929
34	Aminoadipic Acid	0.72608	0.41084	0.68913	48.502	1.5079e-05	2.31e-05
35	Hexanoylcarnitine	0.72377	0.41156	0.68913	90.702	6.0518e-07	1.1777e-06
36	Asparagine	0.66842	0.42954	0.69571	108.8	2.2667e-07	4.8e-07
37	Decanoylcarnitine	0.63349	0.44154	0.69571	35.719	6.4447e-05	9.2804e-05
38	Ornithine	0.62515	0.44448	0.69571	61.499	4.6083e-06	7.8999e-06
39	Tryptophan	0.57523	0.46283	0.69692	7.1629	0.020172	0.023426
40	Choline	0.57053	0.46462	0.69692	11.238	0.0057555	0.0069066
41	Octanoylcarnitine	0.45777	0.5115	0.75159	208.49	6.004e-09	1.8597e-08
42	Citrulline	0.42935	0.52467	0.75552	6.9926	0.021401	0.024458
43	Nicotinic Acid	0.34954	0.56535	0.78088	50.946	1.1842e-05	1.8948e-05
44	Taurine	0.34567	0.56747	0.78088	425.23	9.7567e-11	8.781e-10
45	Kynurenine	0.33256	0.57481	0.78088	9.5331	0.0094048	0.011101
46	Trimethylamine-N-Oxide	0.29185	0.59892	0.78615	190.98	9.8852e-09	2.8469e-08
47	Valine	0.28926	0.60053	0.78615	207.4	6.1868e-09	1.8597e-08
48	Hippuric acid	0.21064	0.65447	0.83176	82.732	9.8722e-07	1.777e-06
49	2-deoxycytidine	0.19029	0.67042	0.83176	49.365	1.3831e-05	2.1649e-05
50	5-Hydroxyindole-3-acetic acid	0.18193	0.67728	0.83176	1090.6	3.7674e-13	2.7125e-11

2.3 ANOVA - Simultaneous Component Analysis (ASCA)

ASCA is a multivariate extension of univariate ANOVA approach. It is designed to identify the major patterns associated with each factor. This implementation supports ASCA model for two factors with one interaction effect. The algorithm first partitions the overall data variance (X) into individual variances induced by each factor (A and B), as well as by the interactions (AB). The formula is shown below with (E) indicates the residual Errors:

$$\mathbf{X = A + B + AB + E}$$

The SCA part applies PCA to A, B, AB to summarize major variations in each partition. Users then detect the major pattern by visualizing the PCA scores plot. MetaboAnalyst also provides model validation to test the significance of the effects associated with main effects. It is based on the Manly's unrestricted permutation of observation then calculate the permuted variation associated with each factor Finally, the permuted values are compared with the original variations The significant variables are identified based on the leverage and the Squared Prediction Errors (SPE) associated with each variables Variables with low SPE and higher leverage are modeled well after the major patterns.

Figure 4 shows the scree plots for each effect model. Figure 5 shows the major patterns associated with factor A. Figure 6 shows the major patterns associated with factor B. Figure 7 shows the major patterns associated with interaction. Figure 8 shows the results of model validations through permutations. Figure 9 shows the important features associated with factor A. Figure 10 shows the important features associated with factor B. Figure 11 shows the features that are important in the interaction.

Table 3 shows features well-modelled by Diet. Table 4 shows features well-modelled by Interaction model. The other details are available as .csv documents in your downloaded zip file.

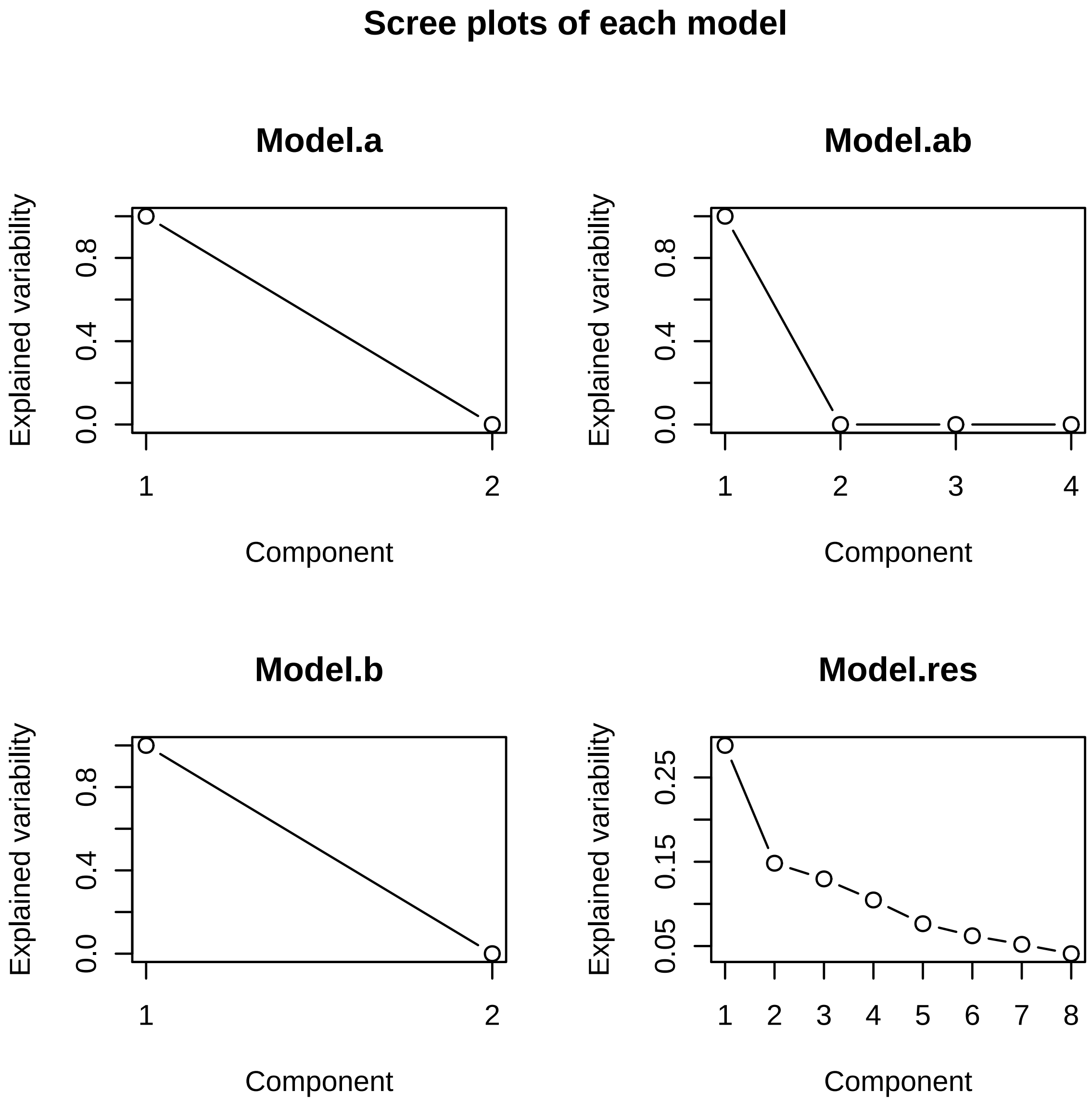

Figure 4: Scree plots for each sub model.

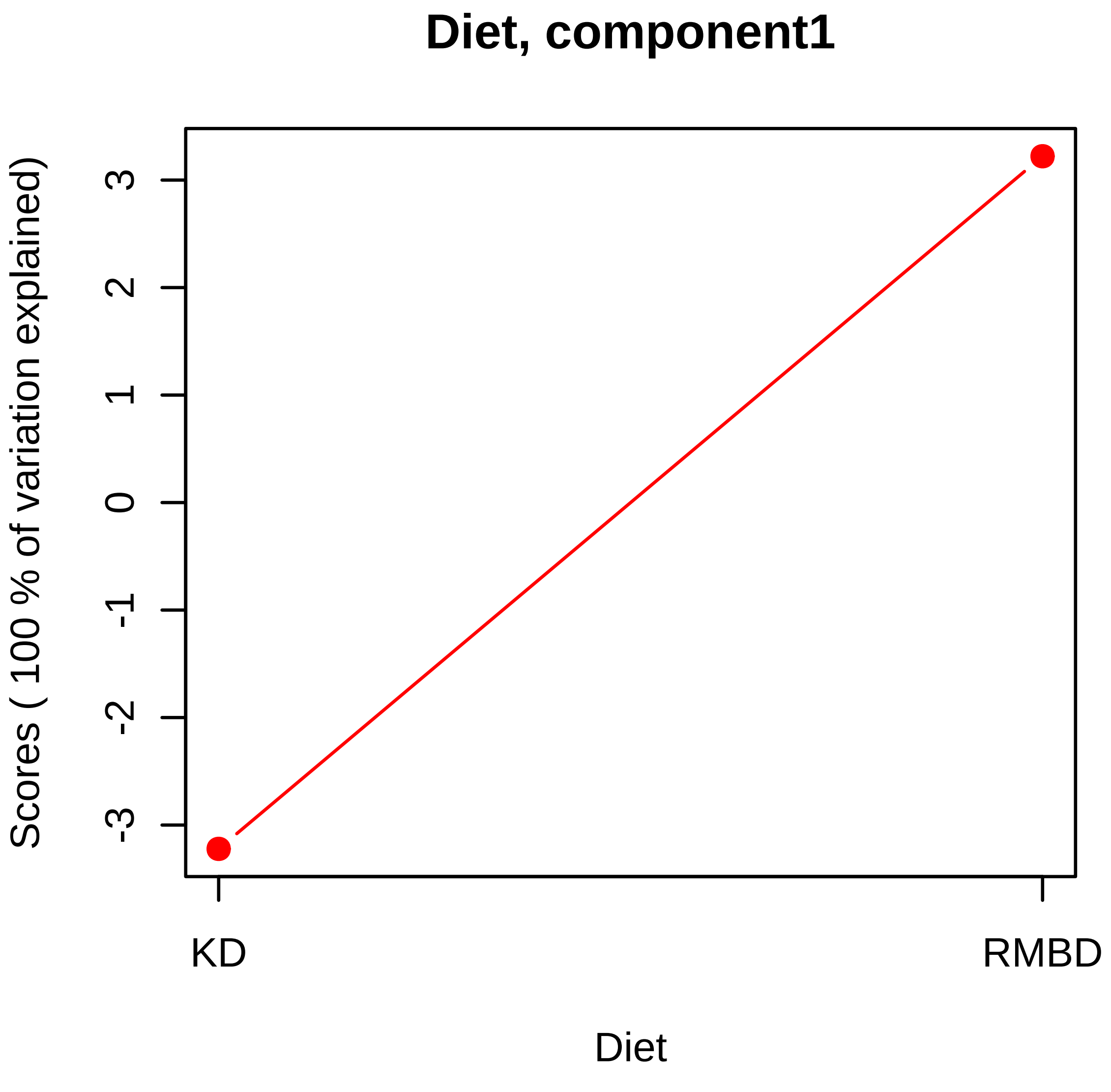

Figure 5: Major patterns associated with Diet

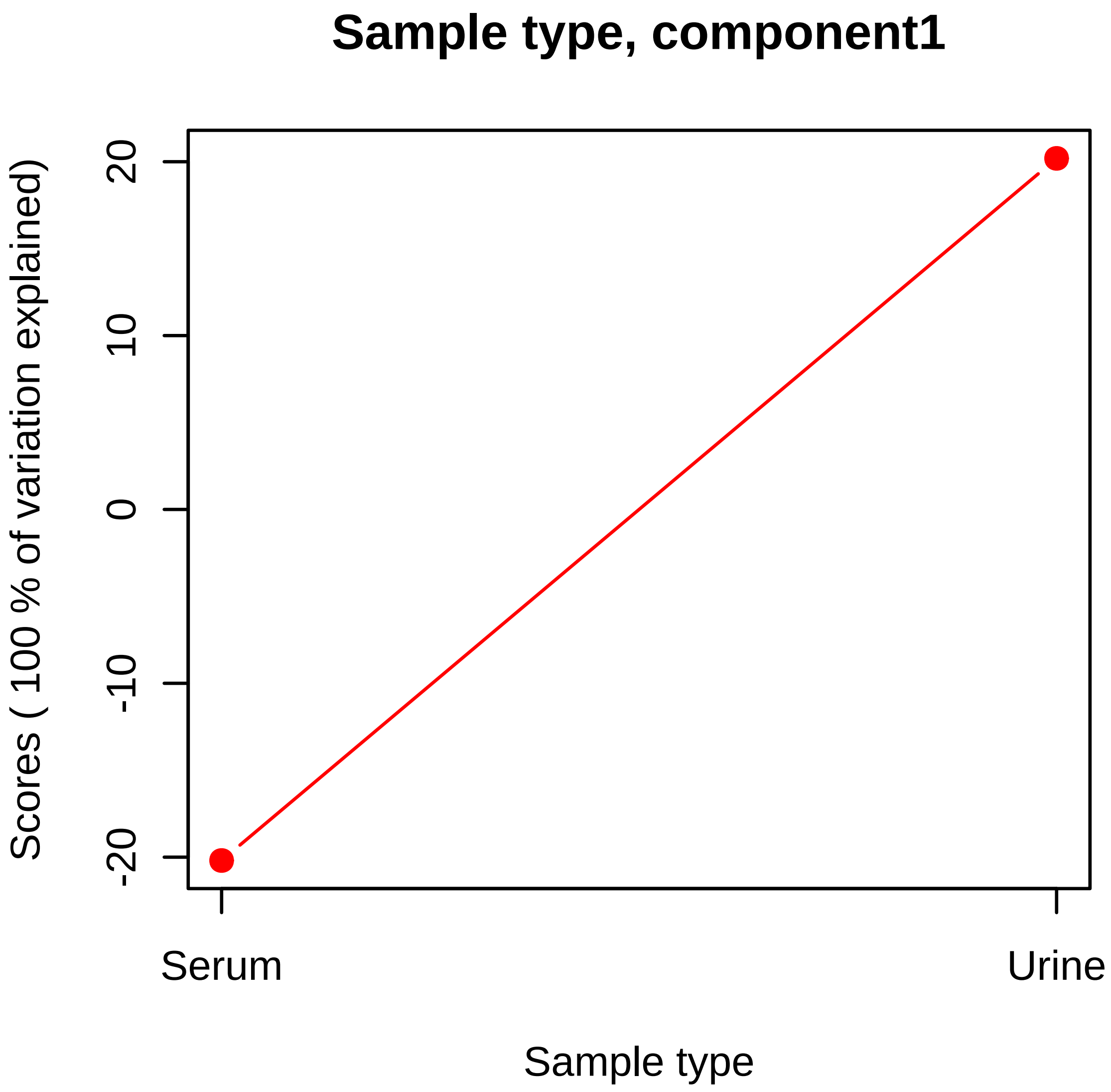

Figure 6: Major patterns associated with Sample type

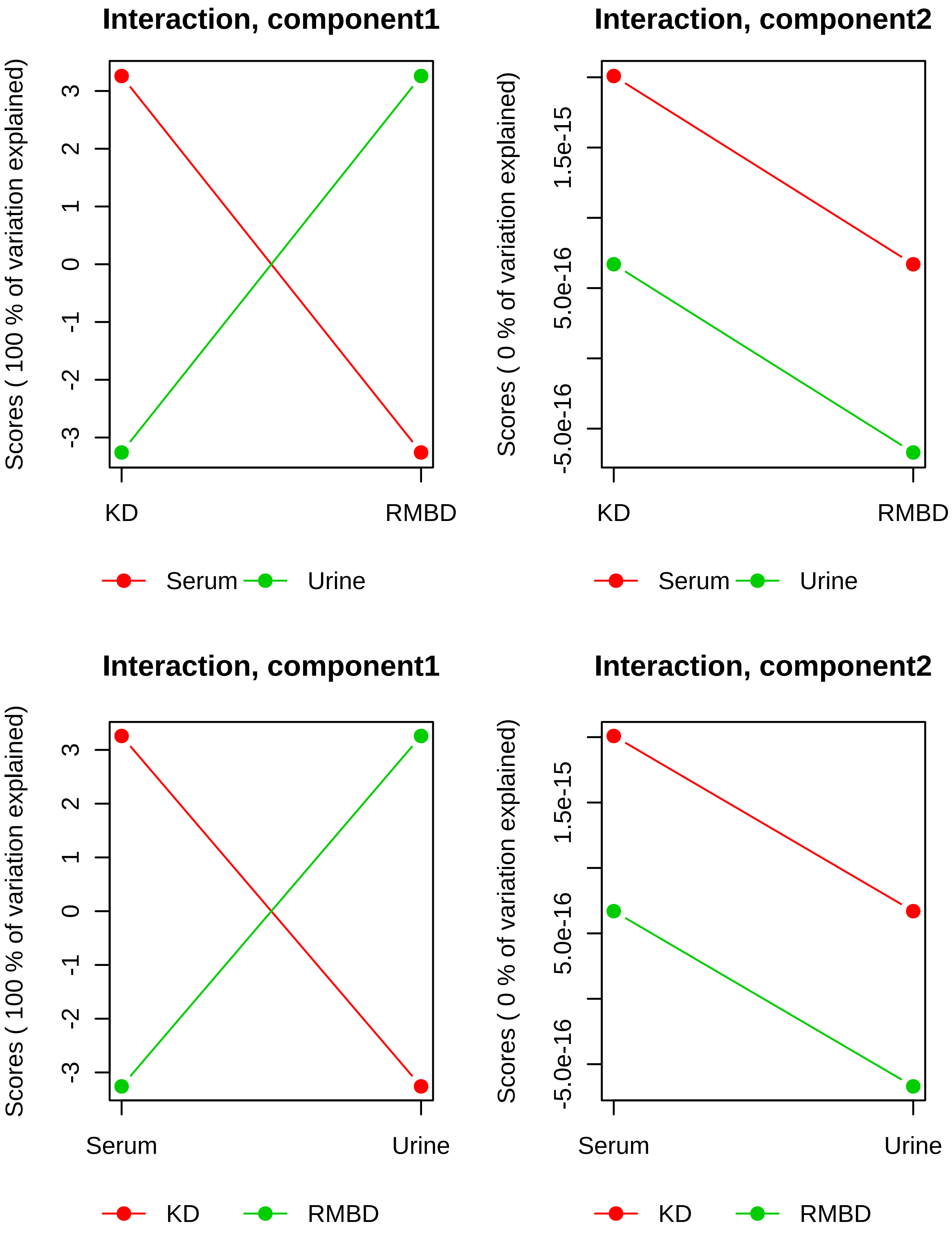

Figure 7: Major patterns associated with the Interaction between the two factors.

Diet

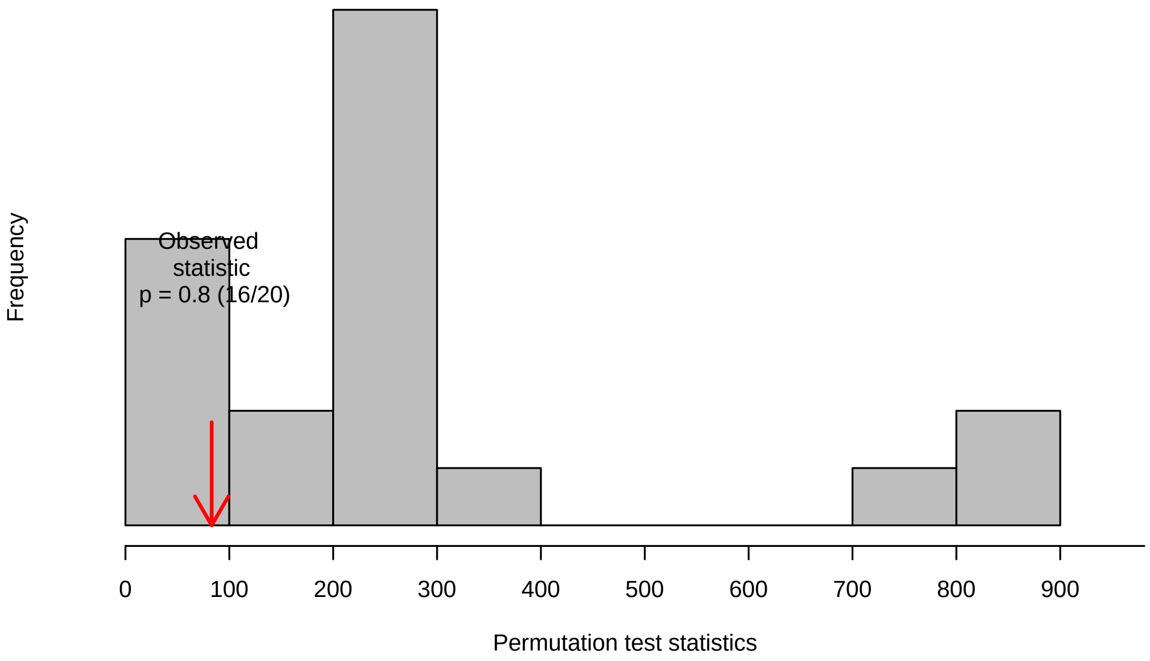

Sample type

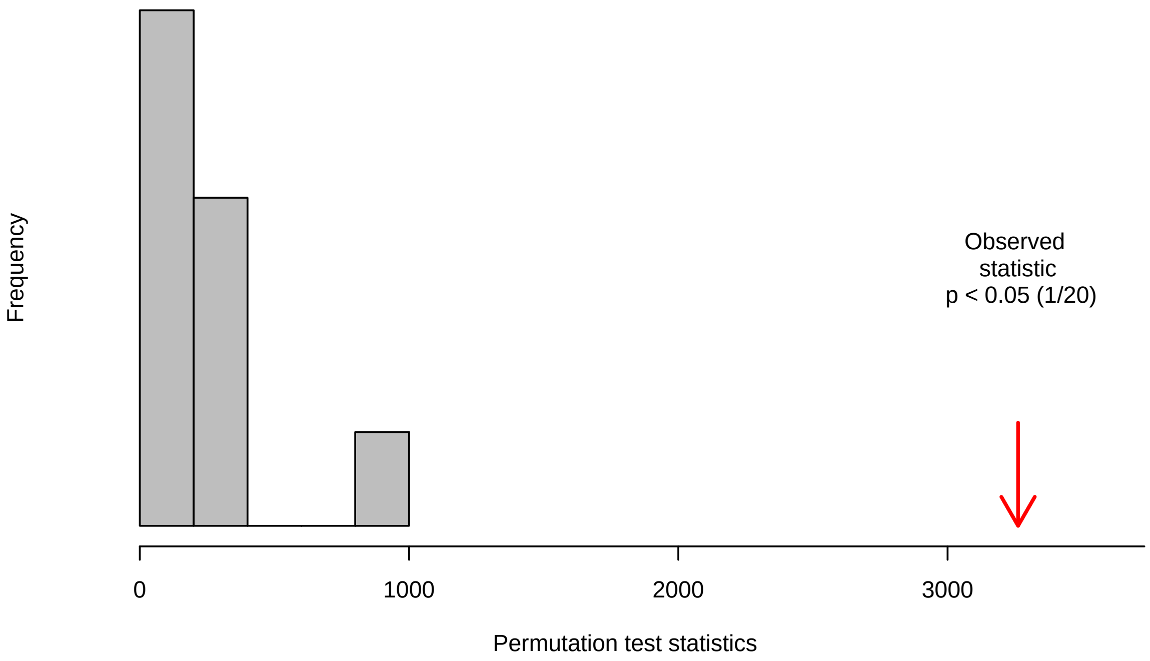

Interaction

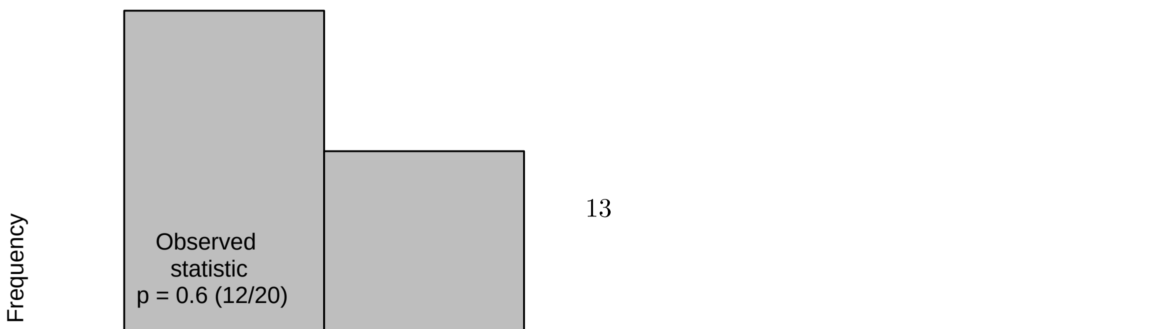

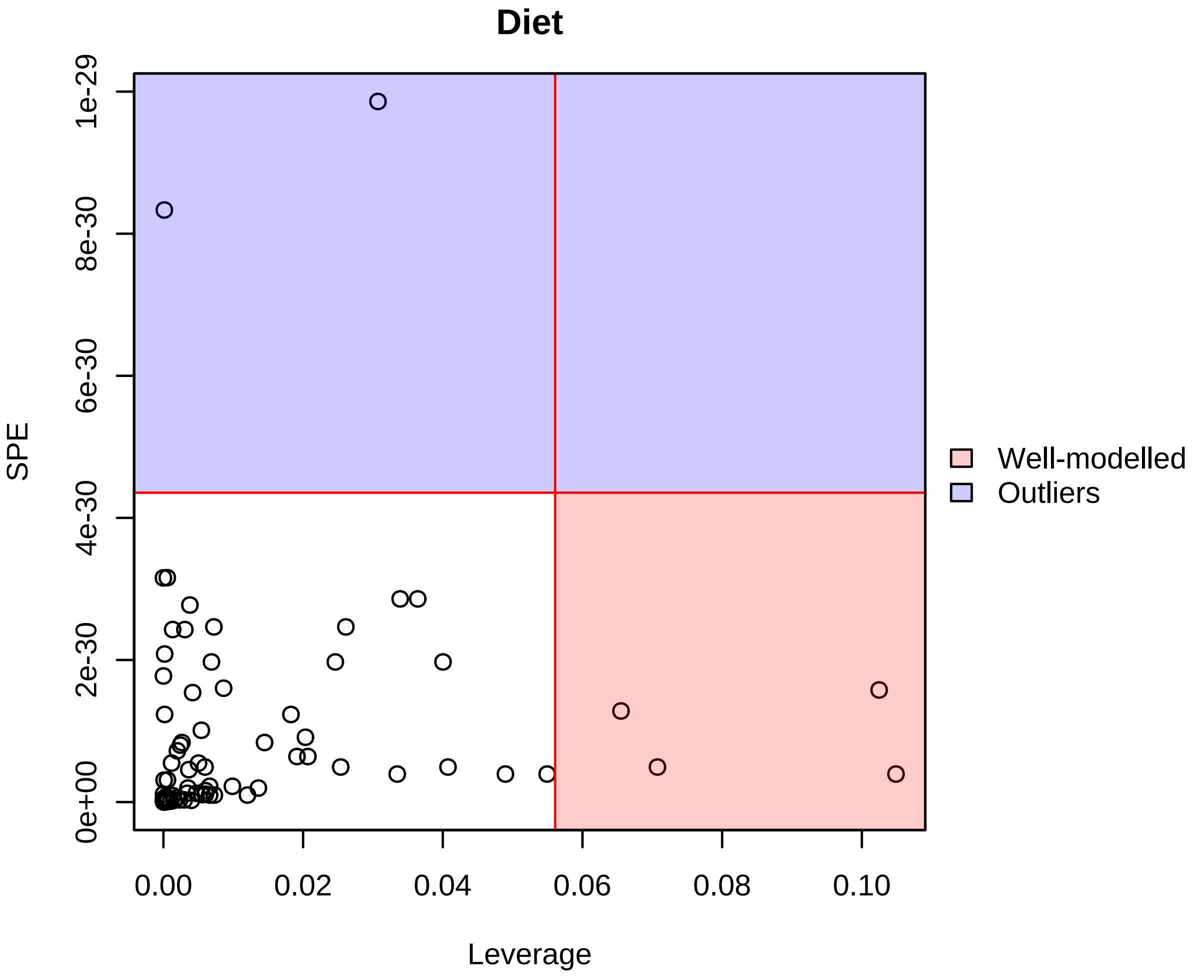

Figure 9: Important variables associated with Diet

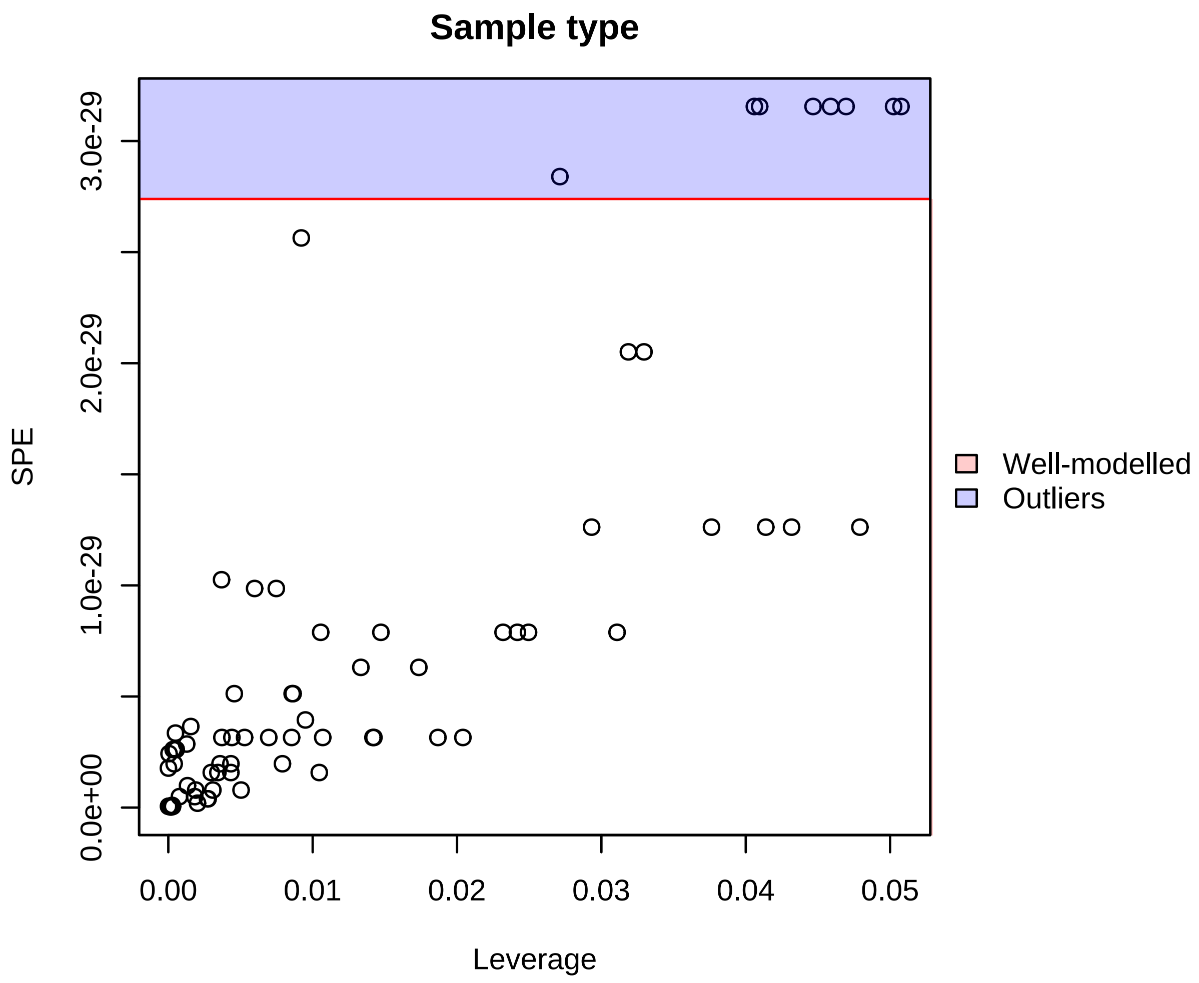

Figure 10: Important variables associated with Sample type

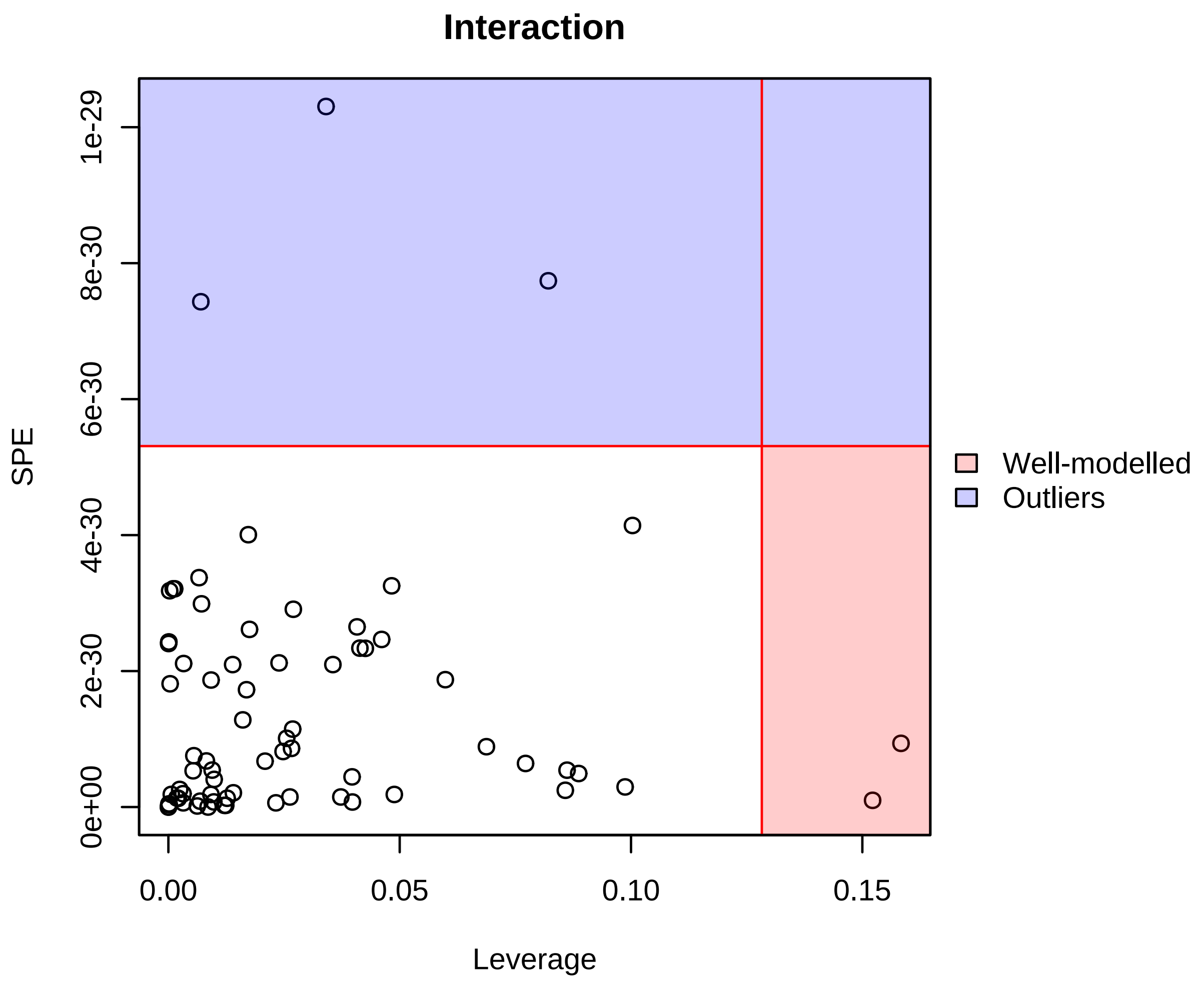

Figure 11: Variables important in interaction between the two factors

Table 3: Important features identified by ASCA. The table shows features that are well modelled by main effect Diet.

	Compounds	Leverage	SPE
1	Homocysteine	0.104889070891959	3.94430452610506e-31
2	Hydroxyproline	0.10247528728229	1.57772181044202e-30
3	Methionine	0.0707361769770091	4.93038065763132e-31
4	Dimethyl Glycine	0.0655155896067098	1.28189897098414e-30

Table 4: Important features identified by ASCA. The table shows features that are well modelled by interaction effect between Diet and Sample type.

	Compounds	Leverage	SPE
1	Hydroxyproline	0.158355243866284	9.36772324949952e-31
2	Methionine	0.152217545393707	9.86076131526265e-32

3 Appendix: R Command History

```
 [1] "mSet<-InitDataObjects(\"conc\", \"ts\", FALSE)"
 [2] "mSet<-SetDesignType(mSet, \"g2\")"
 [3] "mSet<-Read.TextData(mSet, \"Replacing_with_your_file_path\", \"colts\", \"disc\");"
 [4] "mSet<-SanityCheckData(mSet)"
 [5] "mSet<-ReplaceMin(mSet);"
 [6] "mSet<-PreparePrenormData(mSet)"
 [7] "mSet<-Normalization(mSet, \"NULL\", \"LogNorm\", \"NULL\", ratio=FALSE, ratioNum=20)"
 [8] "mSet<-PlotNormSummary(mSet, \"norm_0_\", \"png\", 72, width=NA)"
 [9] "mSet<-PlotSampleNormSummary(mSet, \"snorm_0_\", \"png\", 72, width=NA)"
[10] "mSet<-ANOVA2.Anal(mSet, 0.05, \"fdr\", \"g2\", 1, 1)"
[11] "mSet<-PlotANOVA2(mSet, \"aov2_0_\", \"png\", 72, width=NA)"
[12] "mSet<-PlotANOVA2(mSet, \"aov2_0_\", \"png\", 300, width=NA)"
[13] "mSet<-PlotHeatMap2(mSet, \"heatmap2_0_\", \"png\", 72, width=NA, \"euclidean\",\"ward.D\",\"bw
[14] "mSet<-PlotHeatMap2(mSet, \"heatmap2_1_\", \"png\", 72, width=NA, \"euclidean\",\"ward.D\",\"bw
[15] "mSet<-PlotHeatMap2(mSet, \"heatmap2_1_\", \"png\", 300, width=NA, \"euclidean\",\"ward.D\",\"b
[16] "mSet<-performMB(mSet, 10)"
[17] "mSet<-Perform.ASCA(mSet, 1, 1, 2, 2)"
[18] "mSet<-PlotModelScree(mSet, \"asca_scree_0_\", \"png\", 72, width=NA)"
[19] "mSet<-PlotASCAModel(mSet, \"asca_fa_0_\", \"png\", 72, width=NA, \"a\",FALSE)"
[20] "mSet<-PlotASCAModel(mSet, \"asca_fb_0_\", \"png\", 72, width=NA, \"b\",FALSE)"
[21] "mSet<-PlotInteraction(mSet, \"asca_fab_0_\", \"png\", 72,FALSE, width=NA)"
[22] "mSet<-Perform.ASCA.permute(mSet, 20)"
[23] "mSet<-PlotASCA.Permutation(mSet, \"asca_perm_0_\", \"png\", 72, width=NA)"
[24] "mSet<-CalculateImpVarCutoff(mSet, 0.05, 0.9)"
[25] "mSet<-PlotAscaImpVar(mSet, \"asca_impa_0_\", \"png\", 72, width=NA, \"a\")"
[26] "mSet<-PlotAscaImpVar(mSet, \"asca_impb_0_\", \"png\", 72, width=NA, \"b\")"
[27] "mSet<-PlotAscaImpVar(mSet, \"asca_impab_0_\", \"png\", 72, width=NA, \"ab\")"
[28] "mSet<-SaveTransformedData(mSet)"
[29] "mSet<-PreparePDFReport(mSet, \"guest18175290510364713201\")\n"
```

The report was generated on Sun Apr 19 06:58:23 2020 with R version 3.6.3 (2020-02-29).
