# Supplementary material for: Targeted Metabolomics With Ultraperformance Liquid Chromatography–Mass Spectrometry (UPLC-MS) Highlights Metabolic Differences in Healthy and Atopic Staffordshire Bull Terriers Fed Two Different Diets, A Pilot Study
Source: Front Vet Sci. 2020 Oct 27;7:554296. doi: 10.3389/fvets.2020.554296 (PMC7653775; doi:10.3389/fvets.2020.554296)
Supplement: Supplementary file 4 [file Data_Sheet_1.ZIP › Moore et al_Supplementary files_Analysis reports/Supplementary file 3- Analysis_Report(B1 & B2 end_Diet&Healthstatus).pdf]

Metabolomic Data Analysis with MetaboAnalyst 4.0

Name: guest17738328336337633612

April 20, 2020

1 Data Upload and Integrity Checking

1.1 Upload your data

For two-factor and time-series data, MetaboAnalyst accepts data uploaded in comma separated values (.csv) format. Samples can be in rows or columns. The two factor labels must follow immediately after the sample names. For time-series data, the time points group must be named as **Time**. In addition, the samples collected from the same subject at different time points should be consecutive and ordered by the time points. Users need to specify the data types when uploading their data in order for MetaboAnalyst to select the correct algorithm to process them. Table 1 summarizes the result of the data checking steps.

`Samples are in columns and features in rows. The uploaded file is in comma separated values (.csv) format. The uploaded data file contains 20 (samples) by 79 (compounds) data matrix.`

Table 1: Summary of data processing results

	Features (positive)	Missing/Zero	Features (processed)
D1_06	46	33	79
D2_16	46	33	79
D3_30	47	32	79
D4_49	45	34	79
D5_11	49	30	79
D6_21	47	32	79
D7_47	44	35	79
D8_55	49	30	79
D9_67	46	33	79
R1_23	48	31	79
R2_26	48	31	79
R3_43	47	32	79
R4_45	47	32	79
R5_03	46	33	79
R6_40	46	33	79
R7_59	47	32	79
R8_60	48	31	79
R9_61	46	33	79
R10_65	42	37	79
R11_35	44	35	79

1.2 Data Integrity Check

Before data analysis, a data integrity check is performed to make sure that all the necessary information has been collected. Compound concentration or peak intensity values should all be non-negative numbers. By default, all missing values, zeros and negative values will be replaced by the half of the minimum positive value found within the data (detection limits).

`Samples are in columns and features in rows. The uploaded file is in comma separated values (.csv) format. The uploaded data file contains 20 (samples) by 79 (compounds) data matrix. The data is not time-series data. 2 groups were detected in samples for factor Diet 2 groups were detected in samples for factor Health status Only English letters, numbers, underscore, hyphen and forward slash (/) are allowed. Other special characters or punctuations (if any) will be stripped off. All data values are numeric. A total of 0 (0%) missing values were detected. By default, these values will be replaced by a small value.`

1.3 Data Normalization

The data is stored as a table with one sample per row and one variable (bin/peak/metabolite) per column. The normalization procedures implemented below are grouped into four categories. Sample specific normalization allows users to manually adjust concentrations based on biological inputs (i.e. volume, mass); row-wise normalization allows general-purpose adjustment for differences among samples; data transformation and scaling are two different approaches to make features more comparable. You can use one or combine both to achieve better results.

The normalization consists of the following options:

1. Row-wise procedures:
   - Sample specific normalization (i.e. normalize by dry weight, volume)
   - Normalization by the sum
   - Normalization by the sample median
   - Normalization by a reference sample (probabilistic quotient normalization)[1]
   - Normalization by a pooled or average sample from a particular group
   - Normalization by a reference feature (i.e. creatinine, internal control)
   - Quantile normalization
2. Data transformation :
   - Generalized log transformation (glog 2)
   - Cube root transformation
3. Data scaling:
   - Mean centering (mean-centered only)
   - Auto scaling (mean-centered and divided by standard deviation of each variable)
   - Pareto scaling (mean-centered and divided by the square root of standard deviation of each variable)
   - Range scaling (mean-centered and divided by the value range of each variable)

Figure 1 shows the effects before and after normalization.

[1]Dieterle F, Ross A, Schlotterbeck G, Senn H. *Probabilistic quotient normalization as robust method to account for dilution of complex biological mixtures. Application in 1H NMR metabonomics*, 2006, Anal Chem 78 (13);4281 - 4290

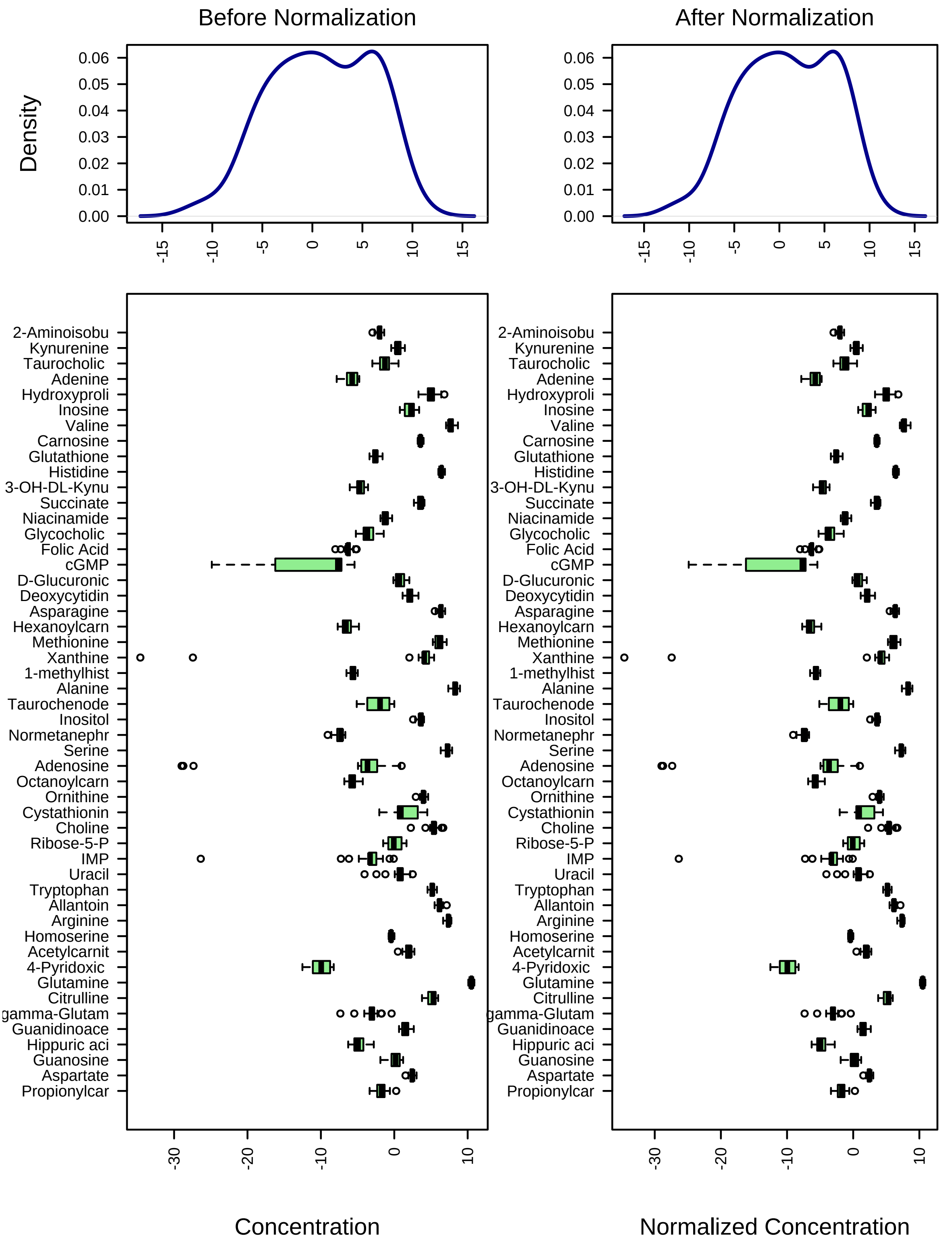

Figure 1: Box plots and kernel density plots before and after normalization. The boxplots show at most 50 features due to space limit. The density plots are based on all samples. Selected methods : Row-wise normalization: N/A; Data transformation: N/A; Data scaling: N/A.

2 Statistical and Machine Learning Data Analysis

For two-factor and time-series data, MetaboAnalyst offers several carefully selected methods for general two-factor and time-series data analysis. They include:

- Data overview:
  - Interactive Principal Component Analysis (iPCA)
  - Two-way Heatmap clustering and visualization
- Univariate method:
  - Two-way between/within-subjects ANOVA
- Multivariate approaches
  - ANOVA-Simultaneous Component Analysis (ASCA)
  - Multivariate Empirical Bayes Analysis (MEBA)

`Please note: MEBA is only applicable to time-series data analyais.`

2.1 Two-way Heatmap Visualization

The heatmap provides direct visualization of all data points in the form of colors squares. The color spectrum intuitively indicates the higher or lower values. Users can choose different clustering algorithms or distance measures to cluster the variables. The samples are ordered by the two factors with default the first factor used for primary ordering. Users can choose to switch the order.

Figure 2 shows the clustering result in the form of a heatmap.

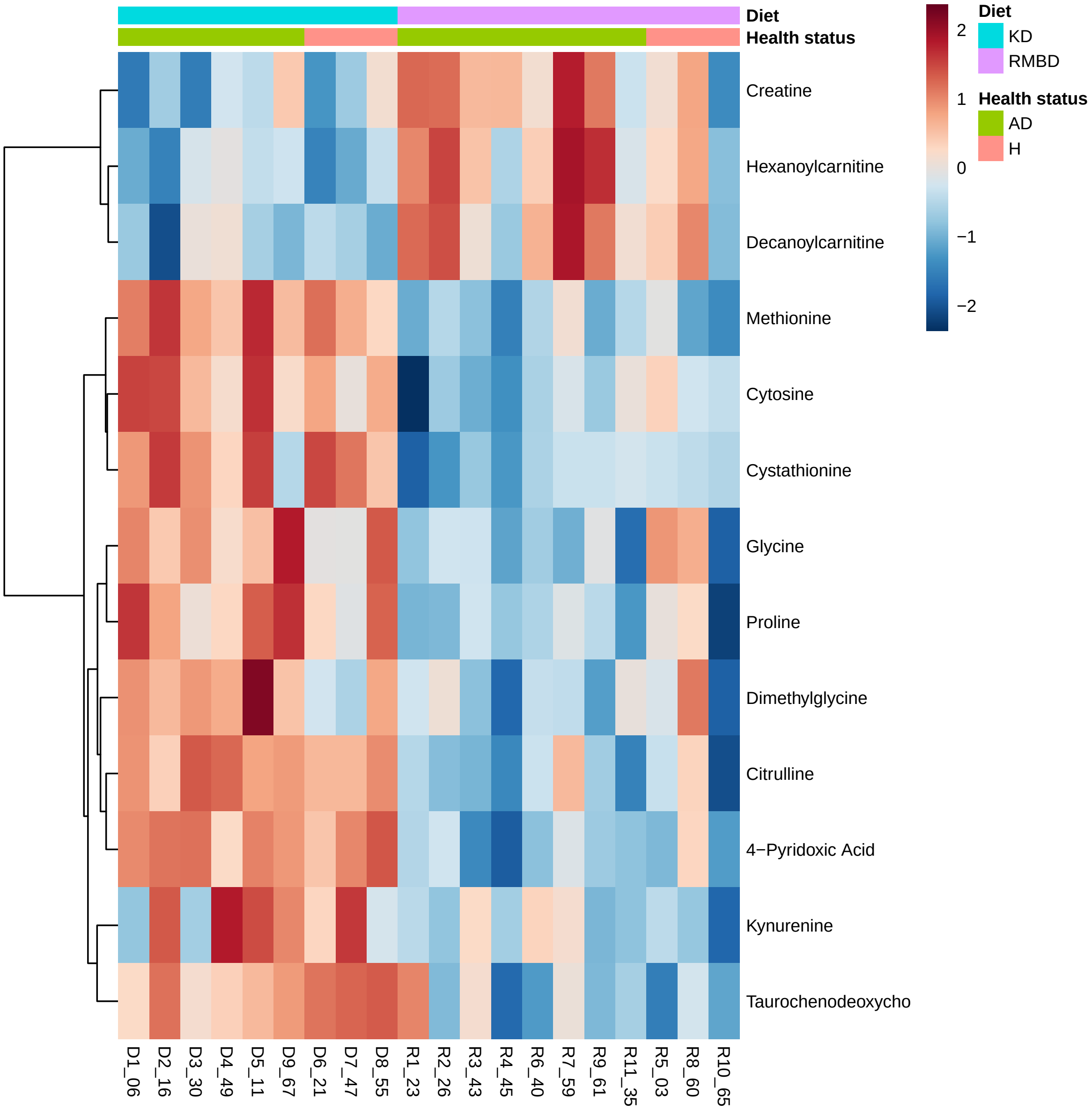

Figure 2: Clustering result shown as heatmap (distance measure using `euclidean`, and clustering algorithm using `ward.D`).

2.2 Univariate Analysis

Univariate analysis methods are the most common methods used for exploratory data analysis. For two-factor data, the basic approach is two-way ANOVA. There are two options - between-subjects ANOVA and within-subjects ANOVA. When samples are all from independent subjects (i.e. general two-way ANOVA), the between-subjects option should be selected. However, time series data contains samples measured from the same subjects from different time points. Therefore within-subjects ANOVA should be used.

Figure 3 shows the important features identified by ANOVA analysis. Table 2 shows the details of these features;

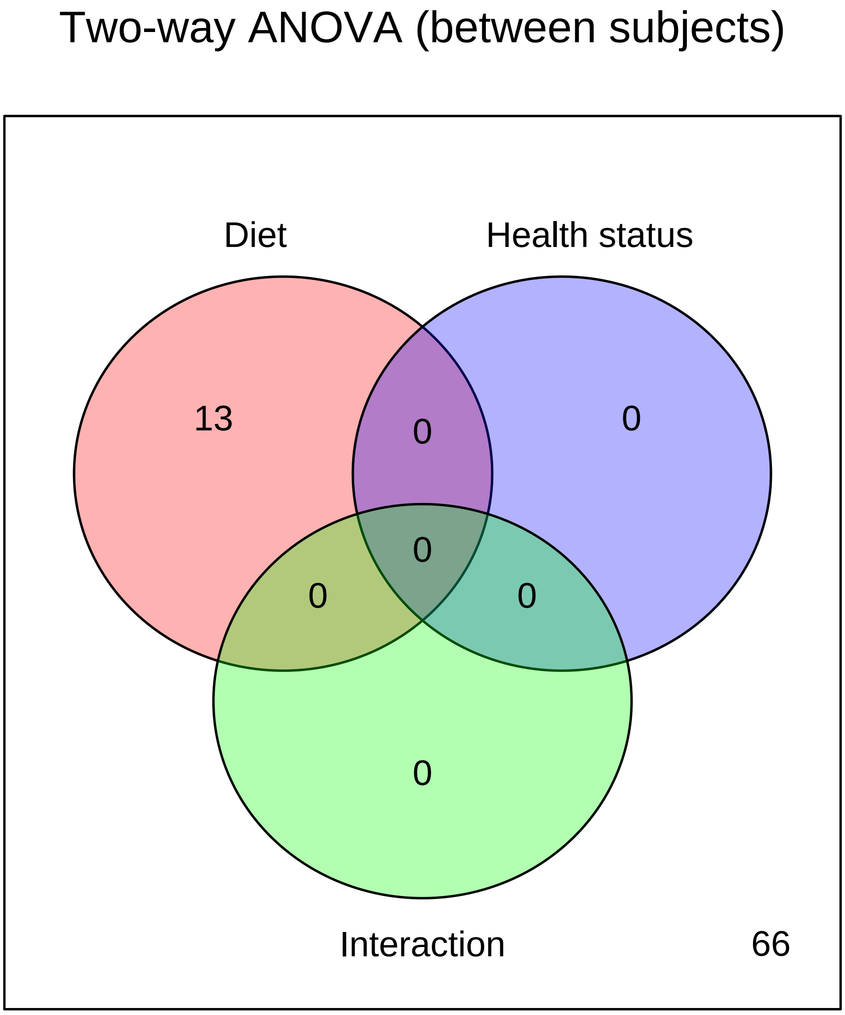

Figure 3: Plot of important features selected by two-way ANOVA.

Table 2: Important features identified by Significant features identified by advanced ANOVA

	Compounds	Diet(F.val)	Diet(raw.p)	Diet(adj.p)	Health status(F.val)	Health status(raw.p)	Health status(adj.p
1	Methionine	50.155	2.5959e-06	0.00013647	0.71064	0.41166	0.81318
2	4-Pyridoxic Acid	47.872	3.4549e-06	0.00013647	0.22242	0.64357	0.86173
3	Cystathionine	32.844	3.0949e-05	0.00081498	1.1789	0.29366	0.81318
4	Citrulline	27.838	7.5305e-05	0.0014873	0.079115	0.78211	0.93694
5	Cytosine	23.64	0.000173	0.0027334	0.31489	0.58247	0.81318
6	Taurochenodeoxycholic Acid	22.711	0.00021058	0.0027727	0.099986	0.75593	0.93694
7	Proline	21.233	0.00029094	0.0032835	0.37639	0.54816	0.81318
8	Hexanoylcarnitine	14.495	0.0015488	0.015294	2.4372	0.13805	0.81318
9	Decanoylcarnitine	12.697	0.0025931	0.020838	0.52946	0.47735	0.81318
10	Glycine	12.639	0.0026377	0.020838	0.14097	0.71225	0.92242
11	Creatine	11.475	0.0037579	0.026988	1.5408	0.2324	0.81318
12	Dimethylglycine	10.295	0.0054788	0.034996	0.67286	0.42411	0.81318
13	Kynurenine	10.143	0.0057588	0.034996	0.97885	0.33721	0.81318

3 Appendix: R Command History

```
 [1] "mSet<-InitDataObjects(\"conc\", \"ts\", FALSE)"
 [2] "mSet<-SetDesignType(mSet, \"g2\")"
 [3] "mSet<-Read.TextData(mSet, \"Replacing_with_your_file_path\", \"colts\", \"disc\");"
 [4] "mSet<-SanityCheckData(mSet)"
 [5] "mSet<-RemoveMissingPercent(mSet, percent=0.5)"
 [6] "mSet<-ImputeVar(mSet, method=\"knn\")"
 [7] "mSet<-PreparePrenormData(mSet)"
 [8] "mSet<-Normalization(mSet, \"NULL\", \"NULL\", \"NULL\", ratio=FALSE, ratioNum=20)"
 [9] "mSet<-PlotNormSummary(mSet, \"norm_0_\", \"png\", 72, width=NA)"
[10] "mSet<-PlotSampleNormSummary(mSet, \"snorm_0_\", \"png\", 72, width=NA)"
[11] "mSet<-ANOVA2.Anal(mSet, 0.05, \"fdr\", \"g2\", 1, 1)"
[12] "mSet<-PlotANOVA2(mSet, \"aov2_0_\", \"png\", 72, width=NA)"
[13] "mSet<-PlotANOVA2(mSet, \"aov2_0_\", \"png\", 300, width=NA)"
[14] "mSet<-PlotHeatMap2(mSet, \"heatmap2_0_\", \"png\", 72, width=NA, \"euclidean\",\"ward.D\",\"bw
[15] "mSet<-PlotHeatMap2(mSet, \"heatmap2_1_\", \"png\", 72, width=NA, \"euclidean\",\"ward.D\",\"bw
[16] "mSet<-PlotHeatMap2(mSet, \"heatmap2_1_\", \"png\", 300, width=NA, \"euclidean\",\"ward.D\",\"b
[17] "mSet<-SaveTransformedData(mSet)"
[18] "mSet<-PreparePDFReport(mSet, \"guest17738328336337633612\")\n"
```

The report was generated on Mon Apr 20 04:09:55 2020 with R version 3.6.3 (2020-02-29).
