# Supplementary material for: Targeted Metabolomics With Ultraperformance Liquid Chromatography–Mass Spectrometry (UPLC-MS) Highlights Metabolic Differences in Healthy and Atopic Staffordshire Bull Terriers Fed Two Different Diets, A Pilot Study
Source: Front Vet Sci. 2020 Oct 27;7:554296. doi: 10.3389/fvets.2020.554296 (PMC7653775; doi:10.3389/fvets.2020.554296)
Supplement: Supplementary file 4 [file Data_Sheet_1.ZIP › Moore et al_Supplementary files_Analysis reports/Supplementary file 16- Analysis_Report(B1&B2_End_Serum_Cadesietc_OnlyRaw).pdf]

Metabolomic Data Analysis with MetaboAnalyst 4.0

Name: guest11599892600985088635

April 20, 2020

1 Data Processing and Normalization

1.1 Reading and Processing the Raw Data

MetaboAnalyst accepts a variety of data types generated in metabolomic studies, including compound concentration data, binned NMR/MS spectra data, NMR/MS peak list data, as well as MS spectra (NetCDF, mzXML, mzDATA). Users need to specify the data types when uploading their data in order for MetaboAnalyst to select the correct algorithm to process them. Table 1 summarizes the result of the data processing steps.

1.1.1 Reading Concentration Data

The concentration data should be uploaded in comma separated values (.csv) format. Samples can be in rows or columns, with class labels immediately following the sample IDs.

Samples are in columns and features in rows. The uploaded file is in comma separated values (.csv) format. The uploaded data file contains 11 (samples) by 82 (compounds) data matrix.

1.1.2 Data Integrity Check

Before data analysis, a data integrity check is performed to make sure that all the necessary information has been collected. The class labels must be present and contain only two classes. If samples are paired, the class label must be from -n/2 to -1 for one group, and 1 to n/2 for the other group (n is the sample number and must be an even number). Class labels with same absolute value are assumed to be pairs. Compound concentration or peak intensity values should all be non-negative numbers. By default, all missing values, zeros and negative values will be replaced by the half of the minimum positive value found within the data (see next section)

1.1.3 Missing value imputations

Too many zeroes or missing values will cause difficulties for downstream analysis. MetaboAnalyst offers several different methods for this purpose. The default method replaces all the missing and zero values with a small values (the half of the minimum positive values in the original data) assuming to be the detection limit. The assumption of this approach is that most missing values are caused by low abundance metabolites (i.e.below the detection limit). In addition, since zero values may cause problem for data normalization (i.e. log), they are also replaced with this small value. User can also specify other methods, such as replace by mean/median, or use K-Nearest Neighbours (KNN), Probabilistic PCA (PPCA), Bayesian PCA (BPCA) method, Singular Value Decomposition (SVD) method to impute the missing values [1]. Please choose the one that is the most appropriate for your data.

[1] Stacklies W, Redestig H, Scholz M, Walther D, Selbig J. *pcaMethods: a bioconductor package, providing PCA methods for incomplete data.*, Bioinformatics 2007 23(9):1164-1167

0 variables were removed for threshold 50 percent. Missing variables were imputated using KNN

1.1.4 Data Filtering

The purpose of the data filtering is to identify and remove variables that are unlikely to be of use when modeling the data. No phenotype information are used in the filtering process, so the result can be used with any downstream analysis. This step can usually improves the results. Data filter is strongly recommended for datasets with large number of variables (> 250) datasets contain much noise (i.e.chemometrics data). Filtering can usually improve your results[2].

For data with number of variables < 250, this step will reduce 5% of variables; For variable number between 250 and 500, 10% of variables will be removed; For variable number bwteen 500 and 1000, 25% of variables will be removed; And 40% of variabled will be removed for data with over 1000 variables. The None option is only for less than 5000 features. Over that, if you choose None, the IQR filter will still be applied. In addition, the maximum allowed number of variables is ***10000***

No data filtering was performed.

Table 1: Summary of data processing results

	Features (positive)	Missing/Zero	Features (processed)
R1_23	49	33	82
R2_26	50	32	82
R3_43	50	32	82
R4_45	49	33	82
R6_40	49	33	82
R7_59	49	33	82
R10_65	45	37	82
R11_35	46	36	82
R5_03	49	33	82
R8_60	50	32	82
R9_61	48	34	82

[2]Hackstadt AJ, Hess AM.*Filtering for increased power for microarray data analysis*, BMC Bioinformatics. 2009; 10: 11.

1.2 Data Normalization

The data is stored as a table with one sample per row and one variable (bin/peak/metabolite) per column. The normalization procedures implemented below are grouped into four categories. Sample specific normalization allows users to manually adjust concentrations based on biological inputs (i.e. volume, mass); row-wise normalization allows general-purpose adjustment for differences among samples; data transformation and scaling are two different approaches to make features more comparable. You can use one or combine both to achieve better results.

The normalization consists of the following options:

1. Row-wise procedures:
   - Sample specific normalization (i.e. normalize by dry weight, volume)
   - Normalization by the sum
   - Normalization by the sample median
   - Normalization by a reference sample (probabilistic quotient normalization)[3]
   - Normalization by a pooled or average sample from a particular group
   - Normalization by a reference feature (i.e. creatinine, internal control)
   - Quantile normalization
2. Data transformation :
   - Generalized log transformation (glog 2)
   - Cube root transformation
3. Data scaling:
   - Mean centering (mean-centered only)
   - Auto scaling (mean-centered and divided by standard deviation of each variable)
   - Pareto scaling (mean-centered and divided by the square root of standard deviation of each variable)
   - Range scaling (mean-centered and divided by the value range of each variable)

Figure 1 shows the effects before and after normalization.

[3]Dieterle F, Ross A, Schlotterbeck G, Senn H. *Probabilistic quotient normalization as robust method to account for dilution of complex biological mixtures. Application in 1H NMR metabonomics*, 2006, Anal Chem 78 (13);4281 - 4290

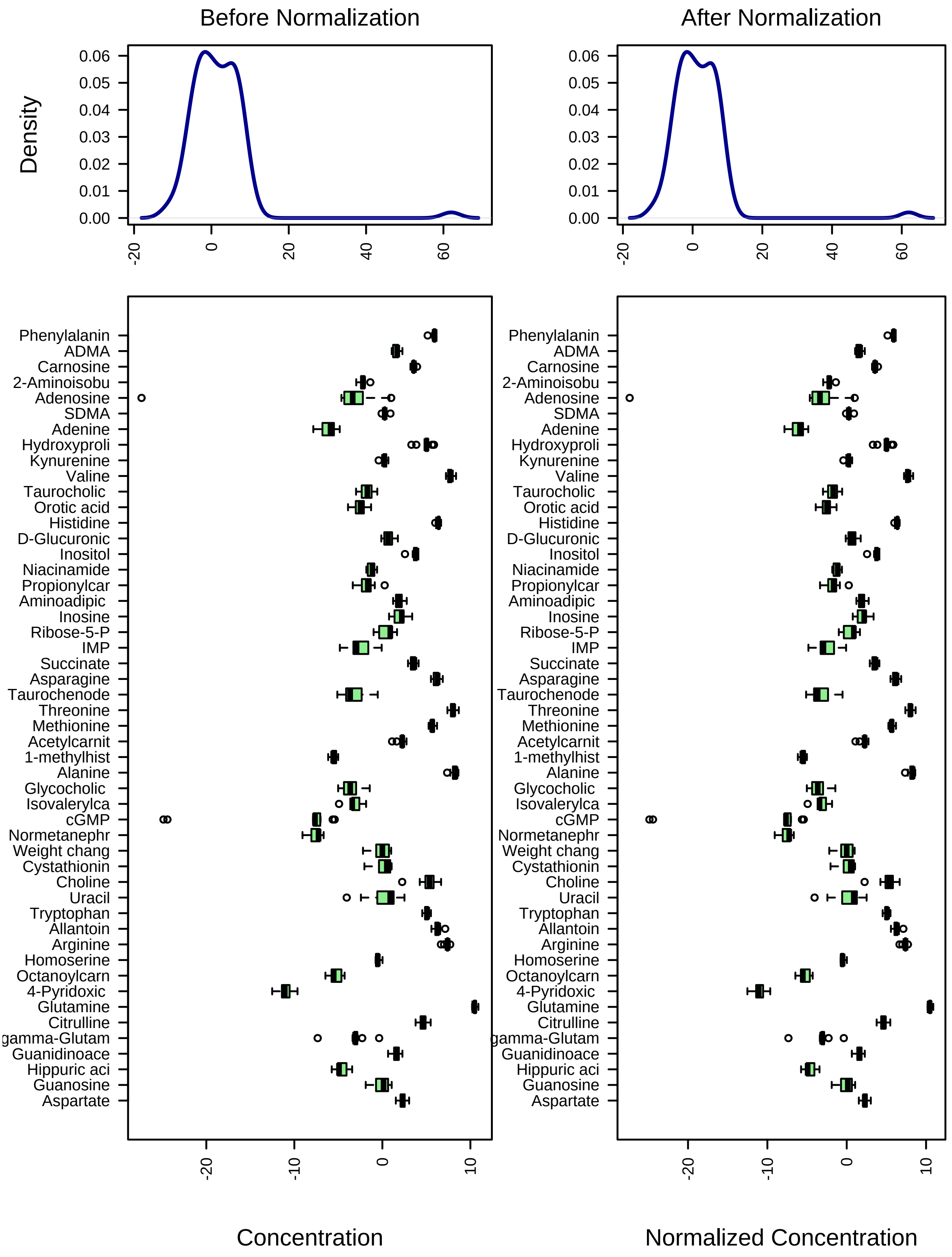

Figure 1: Box plots and kernel density plots before and after normalization. The boxplots show at most 50 features due to space limit. The density plots are based on all samples. Selected methods : Row-wise normalization: N/A; Data transformation: N/A; Data scaling: N/A.

2 Statistical and Machine Learning Data Analysis

MetaboAnalyst offers a variety of methods commonly used in metabolomic data analyses. They include:

1. Univariate analysis methods:
    - Fold Change Analysis
    - T-tests
    - Volcano Plot
    - One-way ANOVA and post-hoc analysis
    - Correlation analysis
2. Multivariate analysis methods:
    - Principal Component Analysis (PCA)
    - Partial Least Squares - Discriminant Analysis (PLS-DA)
3. Robust Feature Selection Methods in microarray studies
    - Significance Analysis of Microarray (SAM)
    - Empirical Bayesian Analysis of Microarray (EBAM)
4. Clustering Analysis
    - Hierarchical Clustering
        - Dendrogram
        - Heatmap
    - Partitional Clustering
        - K-means Clustering
        - Self-Organizing Map (SOM)
5. Supervised Classification and Feature Selection methods
    - Random Forest
    - Support Vector Machine (SVM)

`Please note:  some advanced methods are available only for two-group sample analyais.`

2.1 Univariate Analysis

Univariate analysis methods are the most common methods used for exploratory data analysis. For two-group data, MetaboAnalyst provides Fold Change (FC) analysis, t-tests, and volcano plot which is a combination of the first two methods. All three these methods support both unpaired and paired analyses. For multi-group analysis, MetaboAnalyst provides two types of analysis - one-way analysis of variance (ANOVA) with associated post-hoc analyses, and correlation analysis to identify signficant compounds that follow a given pattern. The univariate analyses provide a preliminary overview about features that are potentially significant in discriminating the conditions under study.

For paired fold change analysis, the algorithm first counts the total number of pairs with fold changes that are consistently above/below the specified FC threshold for each variable. A variable will be reported as significant if this number is above a given count threshold (default $>$ 75% of pairs/variable)

Please note, the purpose of fold change is to compare absolute value changes between two group means. Therefore, the data before column normalization will be used instead. Also note, the result is plotted in log2 scale, so that same fold change (up/down regulated) will have the same distance to the zero baseline.

3 Appendix: R Command History

```
 [1] "mSet<-InitDataObjects(\"conc\", \"stat\", FALSE)"
 [2] "mSet<-Read.TextData(mSet, \"Replacing_with_your_file_path\", \"colu\", \"disc\");"
 [3] "mSet<-SanityCheckData(mSet)"
 [4] "mSet<-RemoveMissingPercent(mSet, percent=0.5)"
 [5] "mSet<-ImputeVar(mSet, method=\"knn\")"
 [6] "mSet<-PreparePrenormData(mSet)"
 [7] "mSet<-Normalization(mSet, \"NULL\", \"NULL\", \"NULL\", ratio=FALSE, ratioNum=20)"
 [8] "mSet<-PlotNormSummary(mSet, \"norm_0_\", \"png\", 72, width=NA)"
 [9] "mSet<-PlotSampleNormSummary(mSet, \"snorm_0_\", \"png\", 72, width=NA)"
[10] "mSet<-Ttests.Anal(mSet, F, 0.05, FALSE, TRUE, FALSE)"
[11] "mSet<-PlotTT(mSet, \"tt_0_\", \"png\", 72, width=NA)"
[12] "mSet<-SaveTransformedData(mSet)"
[13] "mSet<-PreparePDFReport(mSet, \"guest11599892600985088635\")\n"
```

The report was generated on Mon Apr 20 12:12:29 2020 with R version 3.6.3 (2020-02-29).
