# Supplementary material for: Targeted Metabolomics With Ultraperformance Liquid Chromatography–Mass Spectrometry (UPLC-MS) Highlights Metabolic Differences in Healthy and Atopic Staffordshire Bull Terriers Fed Two Different Diets, A Pilot Study
Source: Front Vet Sci. 2020 Oct 27;7:554296. doi: 10.3389/fvets.2020.554296 (PMC7653775; doi:10.3389/fvets.2020.554296)
Supplement: Supplementary file 4 [file Data_Sheet_1.ZIP › Moore et al_Supplementary files_Analysis reports/Supplementary file 19- Analysis report (B1, B2, and B1&B2 Combined datasets_June2019_fixedhealthstatus_(KNN,glog,pcabatchtrans) - B1&B2 end_4groups).pdf]

Metabolomic Data Analysis with MetaboAnalyst 4.0

Name: guest6208315232383390952

August 7, 2020

1 Data Processing and Normalization

1.1 Reading and Processing the Raw Data

MetaboAnalyst accepts a variety of data types generated in metabolomic studies, including compound concentration data, binned NMR/MS spectra data, NMR/MS peak list data, as well as MS spectra (NetCDF, mzXML, mzDATA). Users need to specify the data types when uploading their data in order for MetaboAnalyst to select the correct algorithm to process them. Table 1 summarizes the result of the data processing steps.

1.1.1 Reading Concentration Data

The concentration data should be uploaded in comma separated values (.csv) format. Samples can be in rows or columns, with class labels immediately following the sample IDs.

Samples are in columns and features in rows. The uploaded file is in comma separated values (.csv) format. The uploaded data file contains 20 (samples) by 79 (compounds) data matrix.

1.1.2 Data Integrity Check

Before data analysis, a data integrity check is performed to make sure that all the necessary information has been collected. The class labels must be present and contain only two classes. If samples are paired, the class label must be from -n/2 to -1 for one group, and 1 to n/2 for the other group (n is the sample number and must be an even number). Class labels with same absolute value are assumed to be pairs. Compound concentration or peak intensity values should all be non-negative numbers. By default, all missing values, zeros and negative values will be replaced by the half of the minimum positive value found within the data (see next section)

1.1.3 Missing value imputations

Too many zeroes or missing values will cause difficulties for downstream analysis. MetaboAnalyst offers several different methods for this purpose. The default method replaces all the missing and zero values with a small values (the half of the minimum positive values in the original data) assuming to be the detection limit. The assumption of this approach is that most missing values are caused by low abundance metabolites (i.e.below the detection limit). In addition, since zero values may cause problem for data normalization (i.e. log), they are also replaced with this small value. User can also specify other methods, such as replace by mean/median, or use K-Nearest Neighbours (KNN), Probabilistic PCA (PPCA), Bayesian PCA (BPCA) method, Singular Value Decomposition (SVD) method to impute the missing values [1]. Please choose the one that is the most appropriate for your data.

[1] Stacklies W, Redestig H, Scholz M, Walther D, Selbig J. *pcaMethods: a bioconductor package, providing PCA methods for incomplete data.*, Bioinformatics 2007 23(9):1164-1167

Zero or missing values were replaced by 1/5 of the min positive value for each variable.

1.1.4 Data Filtering

The purpose of the data filtering is to identify and remove variables that are unlikely to be of use when modeling the data. No phenotype information are used in the filtering process, so the result can be used with any downstream analysis. This step can usually improves the results. Data filter is strongly recommended for datasets with large number of variables (> 250) datasets contain much noise (i.e.chemometrics data). Filtering can usually improve your results[2].

For data with number of variables < 250, this step will reduce 5% of variables; For variable number between 250 and 500, 10% of variables will be removed; For variable number bwteen 500 and 1000, 25% of variables will be removed; And 40% of variabled will be removed for data with over 1000 variables. The None option is only for less than 5000 features. Over that, if you choose None, the IQR filter will still be applied. In addition, the maximum allowed number of variables is ***10000***

No data filtering was performed.

Table 1: Summary of data processing results

	Features (positive)	Missing/Zero	Features (processed)
D1_06	78	1	79
D2_16	77	2	79
D3_30	77	2	79
D4_49	77	2	79
D5_11	79	0	79
D9_67	79	0	79
D6_21	79	0	79
D7_47	79	0	79
D8_55	79	0	79
R1_23	79	0	79
R2_26	79	0	79
R3_43	78	1	79
R4_45	77	2	79
R6_40	79	0	79
R7_59	79	0	79
R9_61	79	0	79
R11_35	78	1	79
R5_03	79	0	79
R8_60	79	0	79
R10_65	77	2	79

[2] Hackstadt AJ, Hess AM.*Filtering for increased power for microarray data analysis*, BMC Bioinformatics. 2009; 10: 11.

1.2 Data Normalization

The data is stored as a table with one sample per row and one variable (bin/peak/metabolite) per column. The normalization procedures implemented below are grouped into four categories. Sample specific normalization allows users to manually adjust concentrations based on biological inputs (i.e. volume, mass); row-wise normalization allows general-purpose adjustment for differences among samples; data transformation and scaling are two different approaches to make features more comparable. You can use one or combine both to achieve better results.

The normalization consists of the following options:

1. Row-wise procedures:
   - Sample specific normalization (i.e. normalize by dry weight, volume)
   - Normalization by the sum
   - Normalization by the sample median
   - Normalization by a reference sample (probabilistic quotient normalization)[3]
   - Normalization by a pooled or average sample from a particular group
   - Normalization by a reference feature (i.e. creatinine, internal control)
   - Quantile normalization
2. Data transformation :
   - Generalized log transformation (glog 2)
   - Cube root transformation
3. Data scaling:
   - Mean centering (mean-centered only)
   - Auto scaling (mean-centered and divided by standard deviation of each variable)
   - Pareto scaling (mean-centered and divided by the square root of standard deviation of each variable)
   - Range scaling (mean-centered and divided by the value range of each variable)

Figure 1 shows the effects before and after normalization.

[3]Dieterle F, Ross A, Schlotterbeck G, Senn H. *Probabilistic quotient normalization as robust method to account for dilution of complex biological mixtures. Application in 1H NMR metabonomics*, 2006, Anal Chem 78 (13);4281 - 4290

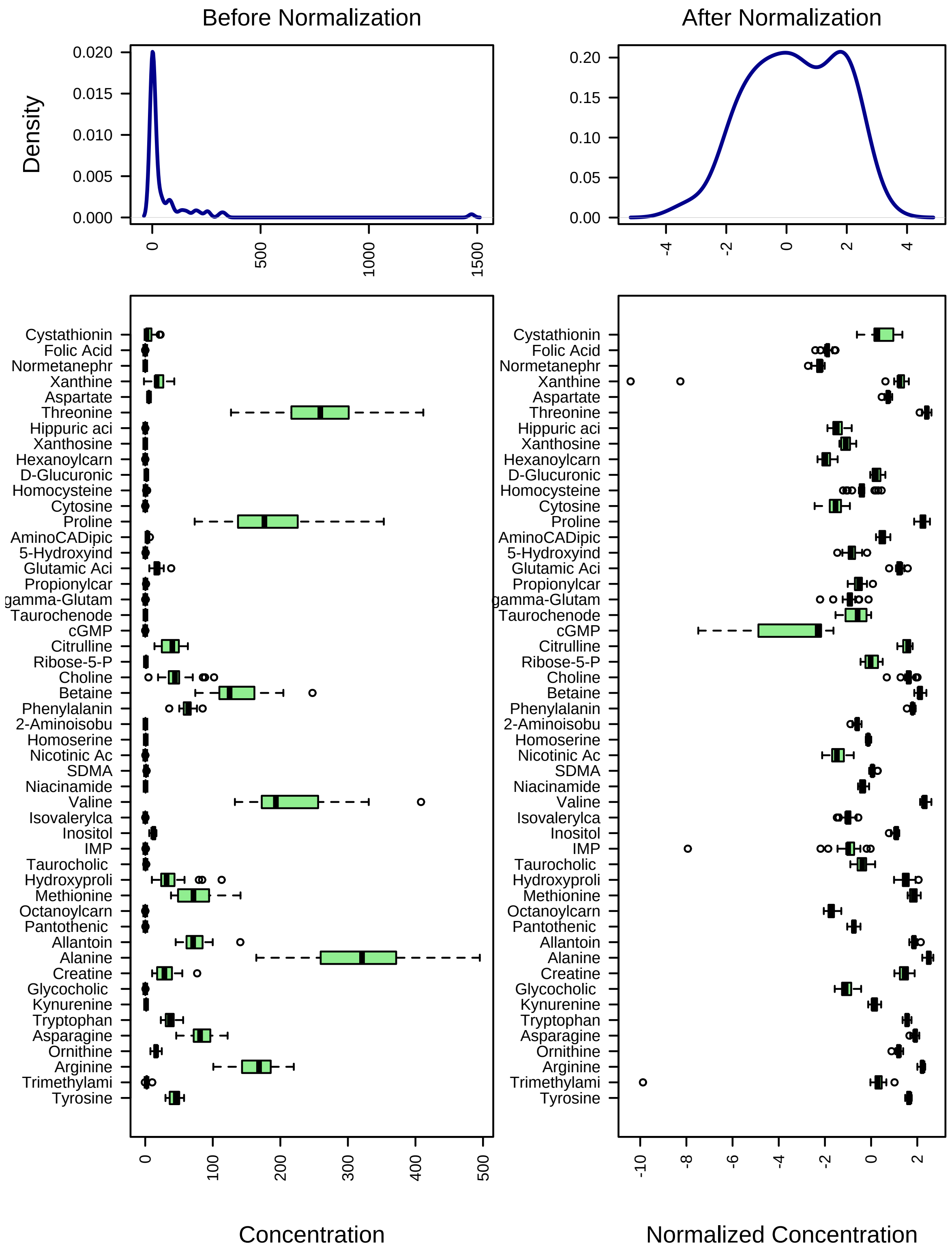

Figure 1: Box plots and kernel density plots before and after normalization. The boxplots show at most 50 features due to space limit. The density plots are based on all samples. Selected methods : Row-wise normalization: N/A; Data transformation: Log10 Normalization; Data scaling: N/A.

2 Statistical and Machine Learning Data Analysis

MetaboAnalyst offers a variety of methods commonly used in metabolomic data analyses. They include:

1. Univariate analysis methods:
   - Fold Change Analysis
   - T-tests
   - Volcano Plot
   - One-way ANOVA and post-hoc analysis
   - Correlation analysis
2. Multivariate analysis methods:
   - Principal Component Analysis (PCA)
   - Partial Least Squares - Discriminant Analysis (PLS-DA)
3. Robust Feature Selection Methods in microarray studies
   - Significance Analysis of Microarray (SAM)
   - Empirical Bayesian Analysis of Microarray (EBAM)
4. Clustering Analysis
   - Hierarchical Clustering
     - Dendrogram
     - Heatmap
   - Partitional Clustering
     - K-means Clustering
     - Self-Organizing Map (SOM)
5. Supervised Classification and Feature Selection methods
   - Random Forest
   - Support Vector Machine (SVM)

`Please note:  some advanced methods are available only for two-group sample analyais.`

2.1 Partial Least Squares - Discriminant Analysis (PLS-DA)

PLS is a supervised method that uses multivariate regression techniques to extract via linear combination of original variables (X) the information that can predict the class membership (Y). The PLS regression is performed using the `plsr` function provided by R `pls` package[4]. The classification and cross-validation are performed using the corresponding wrapper function offered by the `caret` package[5].

To assess the significance of class discrimination, a permutation test was performed. In each permutation, a PLS-DA model was built between the data (X) and the permuted class labels (Y) using the optimal number of components determined by cross validation for the model based on the original class assignment. MetaboAnalyst supports two types of test statistics for measuring the class discrimination. The first one is based on prediction accuracy during training. The second one is separation distance based on the ratio of the between group sum of the squares and the within group sum of squares (B/W-ratio). If the observed test statistic is part of the distribution based on the permuted class assignments, the class discrimination cannot be considered significant from a statistical point of view.[6].

There are two variable importance measures in PLS-DA. The first, Variable Importance in Projection (VIP) is a weighted sum of squares of the PLS loadings taking into account the amount of explained Y-variation in each dimension. Please note, VIP scores are calculated for each components. When more than components are used to calculate the feature importance, the average of the VIP scores are used. The other importance measure is based on the weighted sum of PLS-regression. The weights are a function of the reduction of the sums of squares across the number of PLS components. Please note, for multiple-group (more than two) analysis, the same number of predictors will be built for each group. Therefore, the coefficient of each feature will be different depending on which group you want to predict. The average of the feature coefficients are used to indicate the overall coefficient-based importance.

Figure 2 shows the overview of scores plots; Figure 3 shows the 2-D scores plot between selected components; Figure 4 shows the 3-D scores plot between selected components; Figure 5 shows the loading plot between the selected components;Figure 6 shows the classification performance with different number of components; Figure 7 shows the results of permutation test for model validation; Figure 8 shows important features identified by PLS-DA.

[4]Ron Wehrens and Bjorn-Helge Mevik.*pls: Partial Least Squares Regression (PLSR) and Principal Component Regression (PCR)*, 2007, R package version 2.1-0

[5]Max Kuhn. Contributions from Jed Wing and Steve Weston and Andre Williams.*caret: Classification and Regression Training*, 2008, R package version 3.45

[6]Bijlsma et al.*Large-Scale Human Metabolomics Studies: A Strategy for Data (Pre-) Processing and Validation*, Anal Chem. 2006, 78 567 - 574

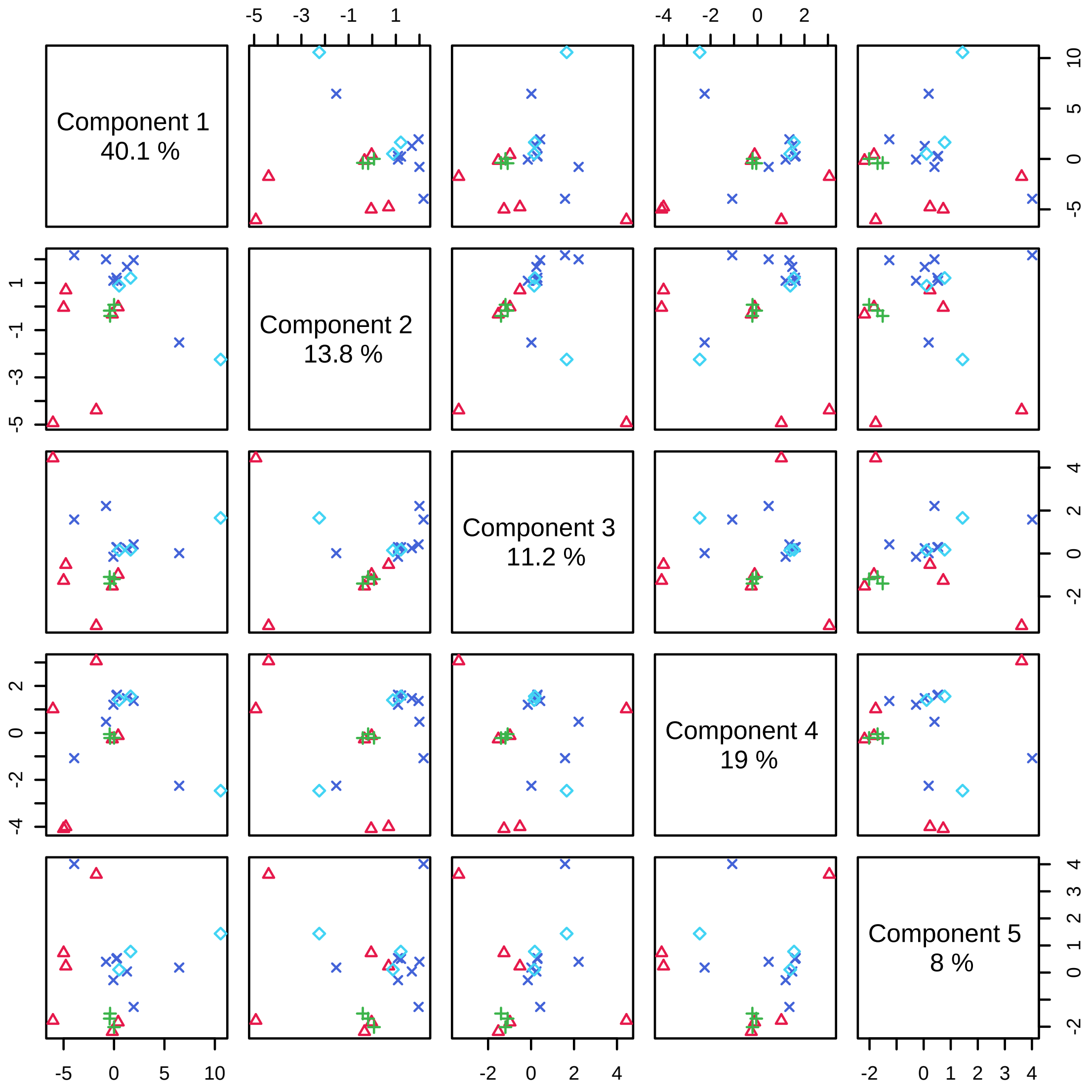

Figure 2: Pairwise scores plots between the selected components. The explained variance of each component is shown in the corresponding diagonal cell.

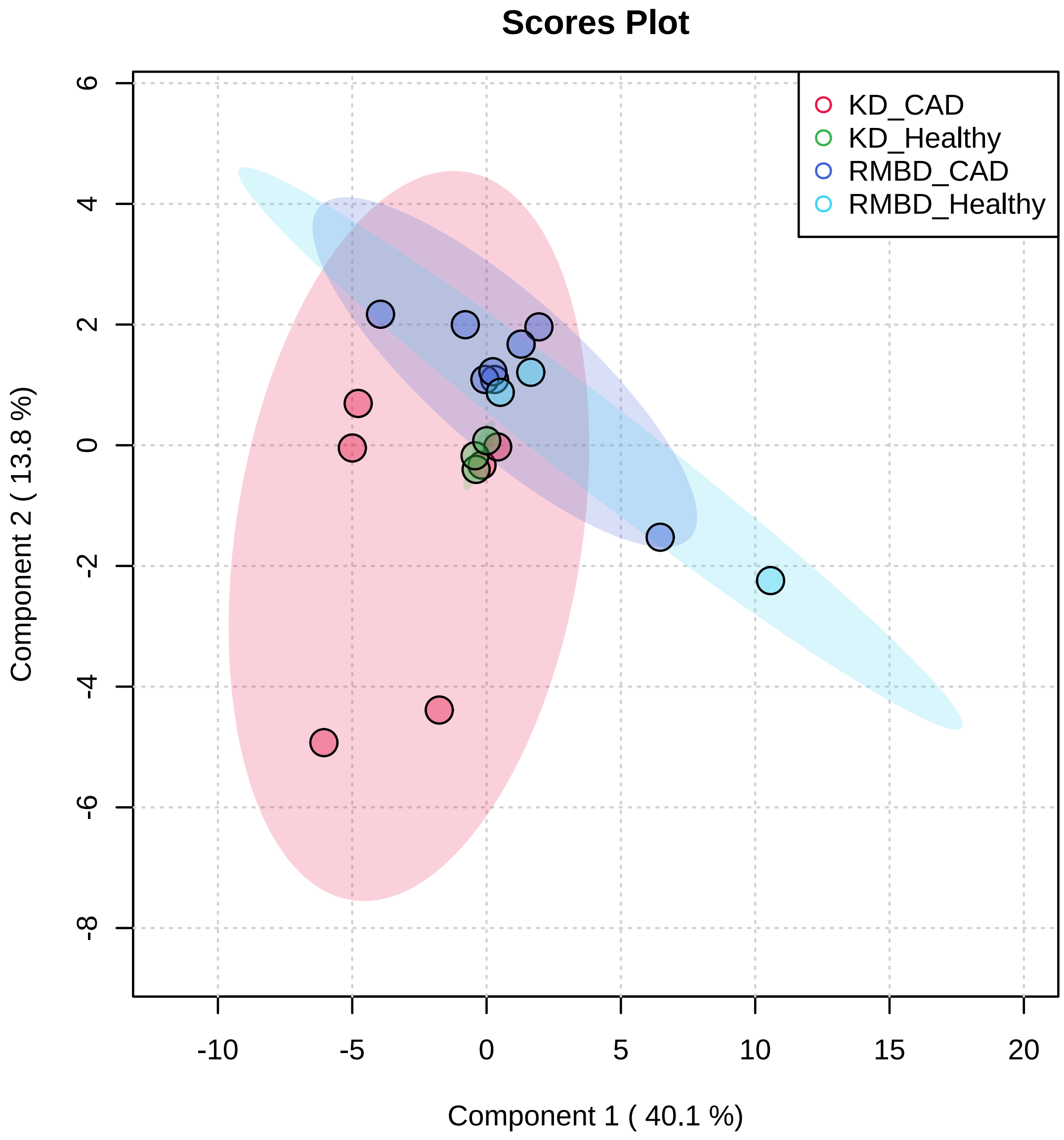

Figure 3: Scores plot between the selected PCs. The explained variances are shown in brackets.

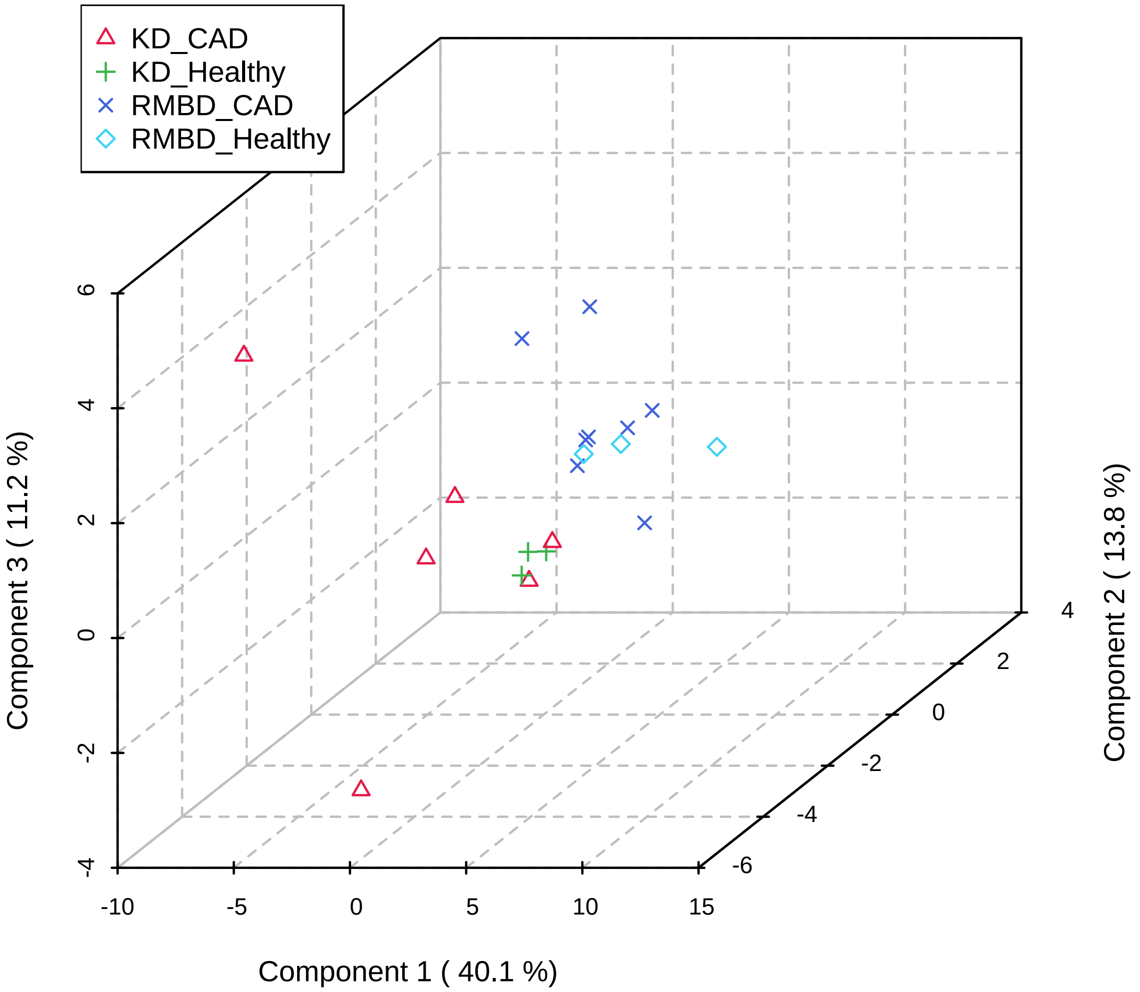

Figure 4: 3D scores plot between the selected PCs. The explained variances are shown in brackets.

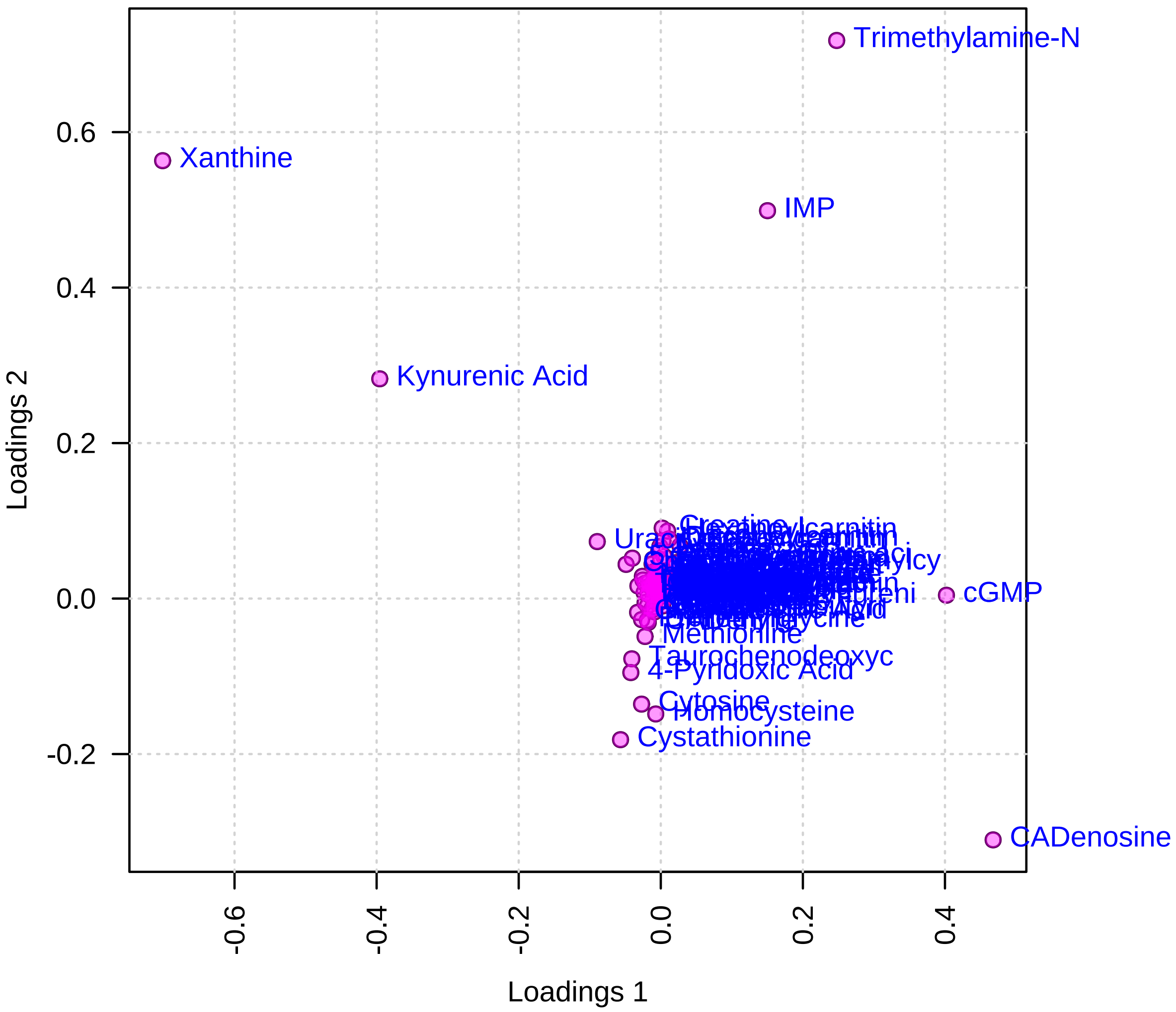

Figure 5: Loadings plot between the selected PCs.

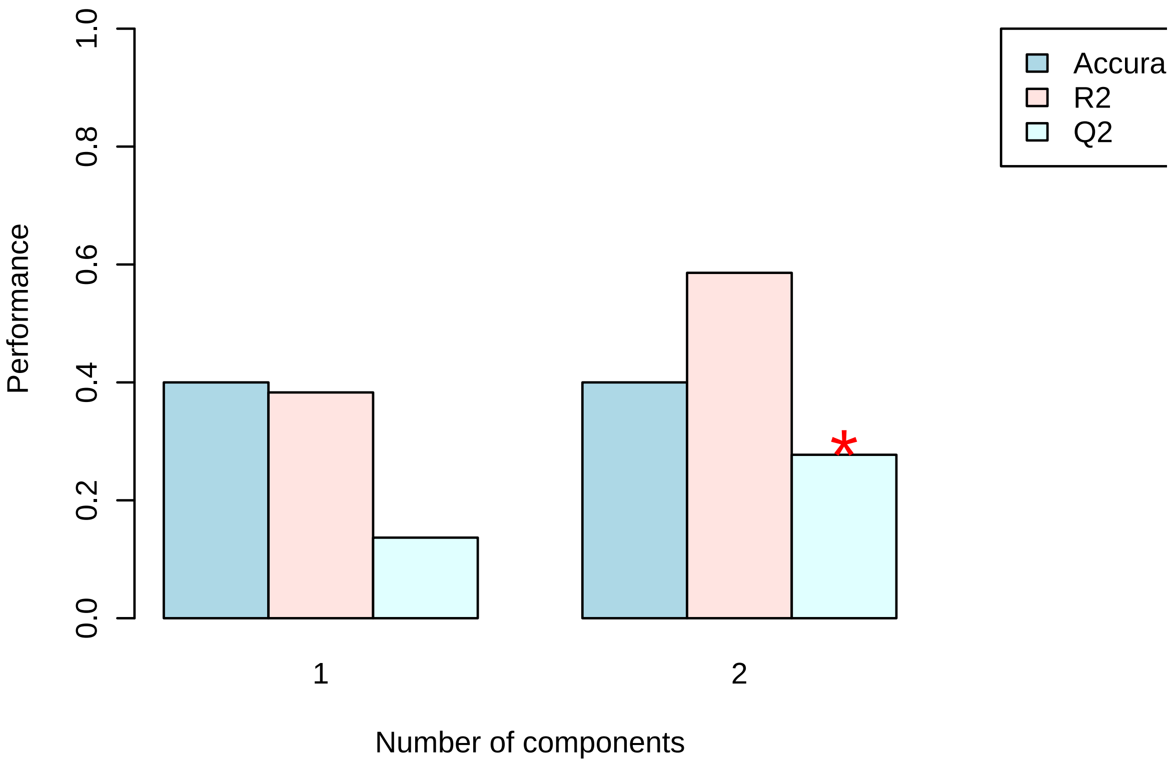

Figure 6: PLS-DA classification using different number of components. The red star indicates the best classifier.

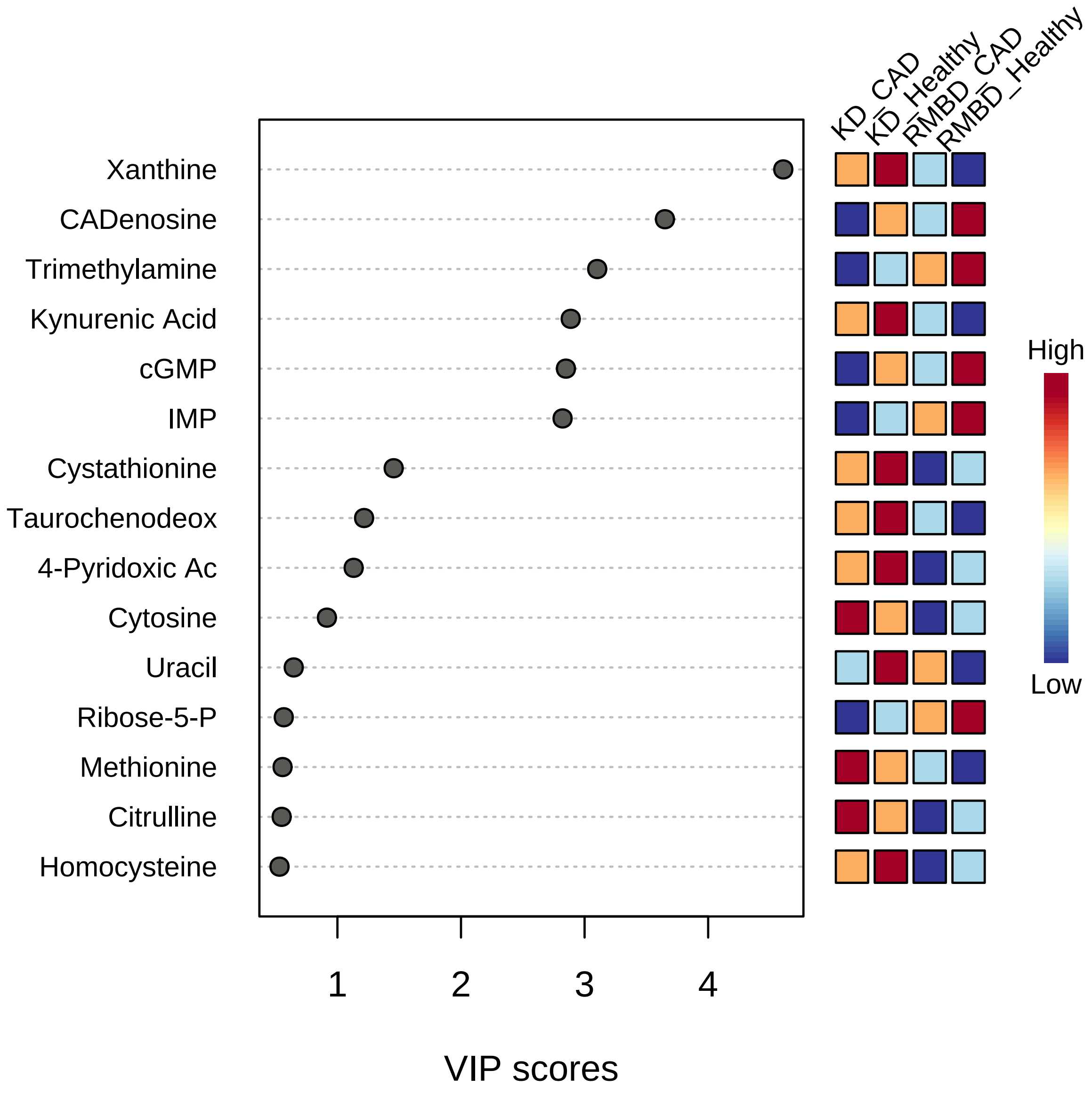

Figure 7: Important features identified by PLS-DA. The colored boxes on the right indicate the relative concentrations of the corresponding metabolite in each group under study.

3 Appendix: R Command History

```
 [1] "mSet<-InitDataObjects(\"conc\", \"stat\", FALSE)"
 [2] "mSet<-Read.TextData(mSet, \"Replacing_with_your_file_path\", \"colu\", \"disc\");"
 [3] "mSet<-SanityCheckData(mSet)"
 [4] "mSet<-ReplaceMin(mSet);"
 [5] "mSet<-PreparePrenormData(mSet)"
 [6] "mSet<-Normalization(mSet, \"NULL\", \"NULL\", \"NULL\", ratio=FALSE, ratioNum=20)"
 [7] "mSet<-PlotNormSummary(mSet, \"norm_0_\", \"png\", 72, width=NA)"
 [8] "mSet<-PlotSampleNormSummary(mSet, \"snorm_0_\", \"png\", 72, width=NA)"
 [9] "mSet<-Normalization(mSet, \"NULL\", \"LogNorm\", \"NULL\", ratio=FALSE, ratioNum=20)"
[10] "mSet<-PlotNormSummary(mSet, \"norm_1_\", \"png\", 72, width=NA)"
[11] "mSet<-PlotSampleNormSummary(mSet, \"snorm_1_\", \"png\", 72, width=NA)"
[12] "mSet<-PLSR.Anal(mSet, reg=TRUE)"
[13] "mSet<-PlotPLSPairSummary(mSet, \"pls_pair_0_\", \"png\", 72, width=NA, 5)"
[14] "mSet<-PlotPLS2DScore(mSet, \"pls_score2d_0_\", \"png\", 72, width=NA, 1,2,0.95,0,0)"
[15] "mSet<-PlotPLS3DScoreImg(mSet, \"pls_score3d_0_\", \"png\", 72, width=NA, 1,2,3, 40)"
[16] "mSet<-PlotPLSLoading(mSet, \"pls_loading_0_\", \"png\", 72, width=NA, 1, 2);"
[17] "mSet<-PlotPLS3DLoading(mSet, \"pls_loading3d_0_\", \"json\", 1,2,3)"
[18] "mSet<-PLSDA.CV(mSet, \"L\",3, \"Q2\")"
[19] "mSet<-PlotPLS.Classification(mSet, \"pls_cv_0_\", \"png\", 72, width=NA)"
[20] "mSet<-PlotPLS.Imp(mSet, \"pls_imp_0_\", \"png\", 72, width=NA, \"vip\", \"Comp. 1\", 15,FALSE)
[21] "mSet<-PLSDA.CV(mSet, \"L\",2, \"Q2\")"
[22] "mSet<-PlotPLS.Classification(mSet, \"pls_cv_1_\", \"png\", 72, width=NA)"
[23] "mSet<-SaveTransformedData(mSet)"
[24] "mSet<-PreparePDFReport(mSet, \"guest6208315232383390952\")\n"
```

The report was generated on Fri Aug 7 06:47:51 2020 with R version 3.6.3 (2020-02-29).
